# Tandyukisins K–P, New Decalin Polyketides from *Trichoderma longicollum* 17007 with Antitubercular Activity Against *Mycobacterium tuberculosis* H37Ra

**DOI:** 10.3390/antibiotics15080789

**Published:** 2026-08-14

**Authors:** Junjie Yu, Shangqian Ning, Yuchang Di, Hongbo Feng, Xiaoying Li, Kun Wang, Rongxuan Wang, Menghan Ma, Sicheng Pan, Chenglin Jiang, Yi Jiang, Tom Hsiang, Jiazhen Chen, Wenhong Zhang, Lixin Zhang, Xueting Liu, Xuelian Zhang, Guoliang Zhu

**Affiliations:** 1 State Key Laboratory of Bioreactor Engineering, East China University of Science and Technology, Shanghai 200237, China; y10220055@mail.ecust.edu.cn (J.Y.); ningshangqian1@163.com (S.N.); 22012406@mail.ecust.edu.cn (H.F.); y12242096@mail.ecust.edu.cn (X.L.); 17356531025@163.com (K.W.); 22012220@mail.ecust.edu.cn (R.W.); y30240611@mail.ecust.edu.cn (M.M.); 24012406@mail.ecust.edu.cn (S.P.); lxzhang@ecust.edu.cn (L.Z.); liuxueting@ecust.edu.cn (X.L.); 2 State Key Laboratory of Genetic Engineering, School of Life Sciences, Fudan University, Shanghai 200438, China; diyc21@m.fudan.edu.cn (Y.D.); 6482@huashan.org.cn (J.C.); zhangwenhong@fudan.edu.cn (W.Z.); 3 Department of Infectious Diseases, Shanghai Key Laboratory of Infectious Diseases and Biosafety Emergency Response, National Medical Center for Infectious Diseases, Huashan Hospital, Fudan University, Shanghai 200040, China; 4 The Key Lab for Research & Development of Actinomycete Resources, Yunnan Institute of Microbiology, Yunnan University, Chenggong District, Kunming 650500, China; lihxu@ynu.edu.cn (C.J.); jiangyi@ynu.edu.cn (Y.J.); 5 School of Environmental Sciences, University of Guelph, Guelph, ON N1G 2W1, Canada; thsian@uoguelph.ca

**Keywords:** fungal natural products, decalin polyketides, *Trichoderma longicollum*, feature-based molecular networking, antimycobacterial activity

## Abstract

**Background:** Endophytic fungi are important sources of structurally diverse antimicrobial natural products, but their potential to yield antitubercular scaffolds remains insufficiently explored. This study aimed to uncover new decalin polyketides from the endophytic fungus, *Trichoderma longicollum* 17007, and to evaluate their biological activity. **Methods:** Feature-based molecular networking (FBMN) analysis of the ethyl acetate extract from rice-based cultures was performed using trichoharzin as a seed compound to guide targeted isolation. Structures were established by HRMS, 1D and 2D NMR spectroscopy, NOESY analysis, and quantum chemical ECD calculations. Cytotoxicity and activity against the avirulent *Mycobacterium tuberculosis* H37Ra strain were evaluated, and genome mining was performed to identify a candidate biosynthetic gene cluster. **Results:** Six new decalin polyketides, tandyukisins K–P (**1**–**6**), and two known analogues, trichoharzin (**7**) and tandyukisin J (**8**), were isolated. Compounds **1**–**6** are C1-*O*-acetylated analogues bearing a 3-methylpentenedioate-type acyl side chain. Comparative ^13^C NMR analysis revealed empirical features useful for assigning side-chain attachment site, double-bond position and geometry. Tandyukisins K (**1**) and P (**6**) showed moderate activity against the avirulent *M. tuberculosis* H37Ra strain, with a MIC value of 12.5 μg/mL. **Conclusions:** These findings expand the structural diversity of decalin polyketides and provide the first evidence of antitubercular activity by this compound family.

## 1. Introduction

Tuberculosis (TB), caused by *Mycobacterium tuberculosis*, remains a major global infectious disease, with an estimated 10.7 million new cases and 1.23 million deaths worldwide in 2024 [1]. The persistent burden of drug-resistant TB underscores the need for new antimycobacterial scaffolds with structural features distinct from existing drugs. Fungal secondary metabolites have historically provided important antimicrobial natural products and continue to represent a valuable source of chemically diverse anti-infective scaffolds [2].

Endophytic fungi inhabit the internal tissues of apparently healthy plants without causing visible disease symptoms, and are an important reservoir of structurally diverse secondary metabolites [3,4]. These metabolites include terpenoids, alkaloids, polyketides, and nonribosomal peptides, many of which exhibit antibacterial, antifungal, cytotoxic, anti-inflammatory, or plant-growth-promoting activities [5,6]. Among metabolite-producing fungi, species of the genus *Trichoderma* are widely recognized as plant-associated and biocontrol fungi, particularly through root colonization, antagonism against phytopathogens, and plant-growth promotion. In addition to these agricultural functions, *Trichoderma* species are prolific producers of secondary metabolites, with more than 400 structurally characterized compounds reported to date [7,8].

Polyketides constitute one of the major secondary-metabolite classes reported from *Trichoderma* species, and have attracted considerable attention because of their structural diversity and biological activities [9]. For example, two polyketides from *Trichoderma* sp. JWM29-10-1 exhibited potent activity against *Helicobacter pylori*, including multidrug-resistant strains, with MIC values of 2–8 μg/mL [10]. Decalin derivatives from *Trichoderma lentiforme* ML-P8-2 also showed antifungal activity against *Penicillium italicum*, with the most active compounds displaying MIC values of 6.25 μM [11]. These findings highlight *Trichoderma*-derived polyketides as valuable sources of structurally diverse and biologically active natural products. Despite extensive studies on *Trichoderma* species as biocontrol and secondary-metabolite-producing fungi, the chemical diversity of many individual species and strains remains incompletely characterized [9,12]. *Trichoderma longicollum* was described as a new species in 2023 and has so far received limited chemical investigation [13,14]. Therefore, investigation of *T. longicollum* may provide access to previously uncharacterized fungal polyketides with potential antimycobacterial activity.

Advances in genome-mining platforms, including antiSMASH, ClusterFinder, SMURF, and PRISM, have greatly facilitated the detection and annotation of biosynthetic gene clusters [15,16,17,18]. In parallel, LC–MS/MS-based tools such as Global Natural Products Social Molecular Networking (GNPS) and the Mass Spectrometry Search Tool (MASST) have improved metabolite dereplication, spectral annotation, and the prioritization of candidate natural products [19,20]. The integration of genomic and metabolomic data can further link biosynthetic potential to metabolite families and guide the targeted isolation of novel compounds. For example, combined antiSMASH analysis and LC–MS/MS molecular networking of extracts from *Pseudoalteromonas* bacteria facilitated the discovery of dibromoalterochromides D/D′ [21]. Building on classical MS/MS-based molecular networking, complementary workflows including feature-based molecular networking (FBMN), ion identity molecular networking (IIMN), building-block-based molecular networking (BBMN), and bioactivity-based molecular networking (BMN) have further improved feature-level resolution, grouped multiple ion species derived from the same compound, prioritized metabolites containing characteristic structural motifs, and correlated molecular features with biological activity, respectively [22,23,24,25]. Collectively, these approaches have accelerated the dereplication and targeted discovery of structurally novel and biologically active natural products.

In this study, we explored the chemical diversity of *T. longicollum* 17007 grown on rice medium. By combining antiSMASH-based biosynthetic gene cluster (BGC) annotation with FBMN analysis of the ethyl acetate (EtOAc) extract, we prioritized a series of putative tandyukisin analogues for targeted isolation. Structure elucidation using UV spectroscopy, HR-ESI-MS, NMR spectroscopy, and quantum-chemical calculations led to the characterization of six new decalin derivatives, tandyukisins K–P (**1**–**6**), together with two known analogues, trichoharzin (**7**) [26] and tandyukisin J (**8**) [11]. Compounds **1**–**8** were further evaluated for activity against the avirulent *M. tuberculosis* H37Ra strain and for cytotoxicity against selected human cell lines.

## 2. Results

### 2.1. Characterization and Identification of Fungal Strain

Strain 17007 formed a circular colony with pale aerial mycelia and green sporulation after cultivation on PDA at 28 °C for 10 days (Figure S1A). In the ITS-based phylogenetic tree, strain 17007 clustered with *Trichoderma longicollum* BCRC 19F0006 with 99% bootstrap support (Figure S1B). Based on its morphological characteristics and phylogenetic placement, strain 17007 was identified as *T. longicollum*. The strain was deposited in the China General Microbiological Culture Collection Center under accession number CGMCC 40622.

### 2.2. FBMN-Guided Discovery of New Decalin Polyketides from T. longicollum 17007

**Figure 1:**
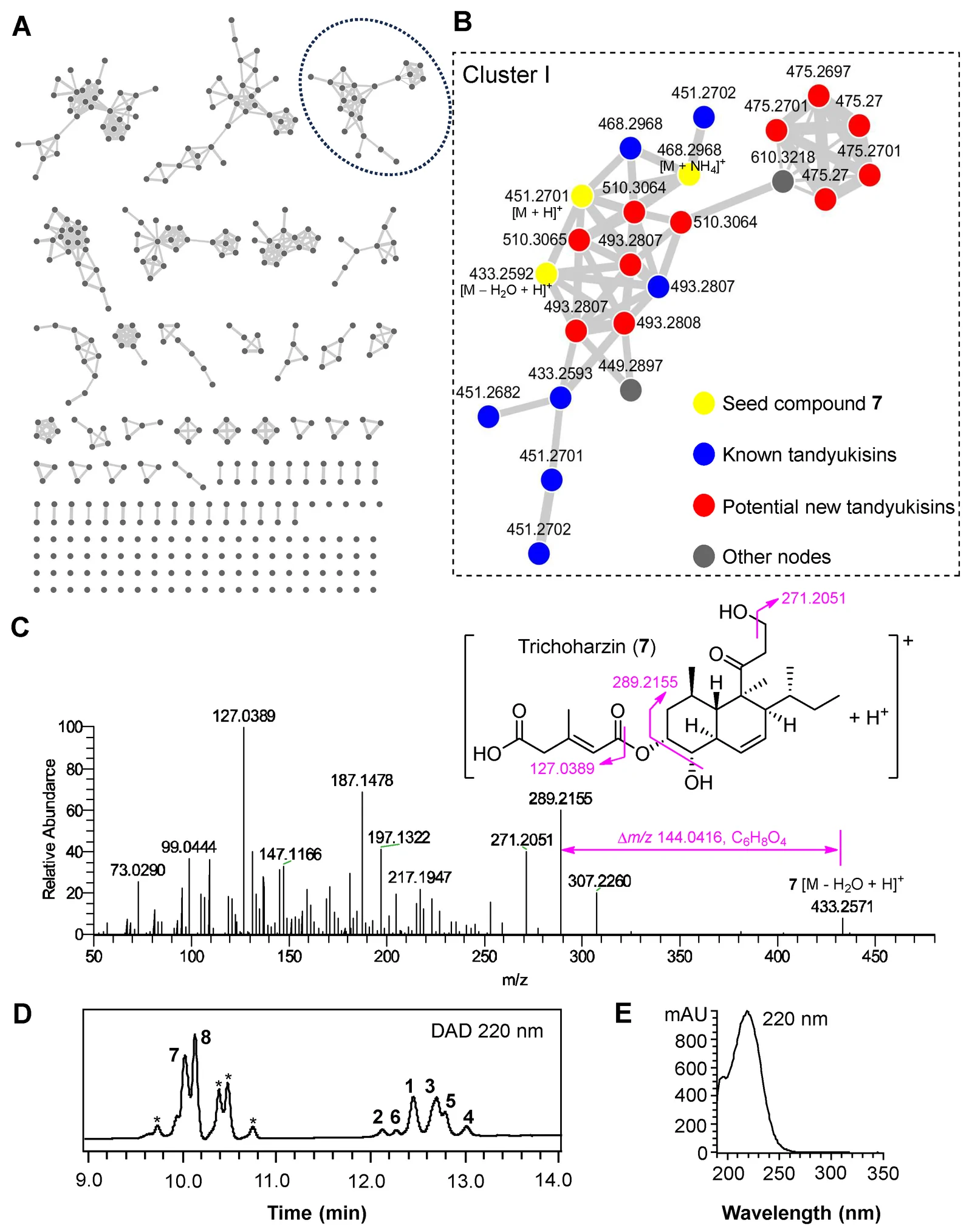
FBMN-guided characterization of new decalins from *T. longicollum* 17007. (**A**) Overview of the molecular networking. The dotted circle indicates Cluster I selected for further analysis. (**B**) Expanded view of Cluster I containing decalin products. Trichoharzin (**7**) was used as seed compound (yellow circle) in FBMN analysis. (**C**) MS/MS analysis of decalins revealed a common fragment ion at *m*/*z* 289.2155 under NCE mode. (**D**) HPLC-UV chromatogram of decalin fractions E2G_1–3_ examined at 220 nm. Numbers **1**–**8** indicate the corresponding compounds. Asterisks (*) indicate uncharacterized peaks tentatively assigned as isomeric analogues of compound **7** based on their similar UV and HRMS profiles (Figure S2). (**E**) UV spectra of tandyukisins.

The fungal strain *T. longicollum* 17007 was fermented with rice medium and previously reported decalin trichoharzin (**7**) were characterized from the crude extract based on the HR-MS data and UV spectrum (Figure 1) [26]. For detection of new decalin polyketides from the fermentation products, FBMN analysis was conducted using trichoharzin (**7**) as the seed compound, and the related nodes were found within Cluster I (Figure 1A,B, Table S1) consisting of 23 nodes with similar MS/MS patterns. Further analysis of LC-MS/MS data suggested several nodes with unreported *m*/*z* values (493.2807, 510.3064, and 475.2701) shared a fragment ion of *m*/*z* 289.2155 (C_19_H_29_O_2_) (Figure 1C and Table S1), generated from the decalin core structure. These *m*/*z* values were used to locate and guide the purification of new decalin derivatives from subfraction E2G_1–3_. Six new decalin derivatives, namely tandyukisins K–P (**1**–**6**), along with two known compounds trichoharzin (**7**) and tandyukisin J (**8**), were isolated from *T. longicollum* 17007. Through comprehensive spectroscopic analysis and quantum chemical calculations, the chemical structures of these compounds were elucidated.

### 2.3. Structure Elucidation of New Compounds

Previously reported tandyukisin-type polyketides share a highly oxygenated and alkylated decalin core but differ mainly in whether the unsaturated acyl side chain is esterified at C8 or C9, the position and geometry of the side-chain double bond, the degree of terminal esterification, and the substitution pattern at C1 (Figure S3) [10,11,26,27,28,29,30]. These recurring structural variations were accompanied by characteristic differences in their ^13^C NMR data, providing useful comparative features for structural assignment.

**Figure 2:**
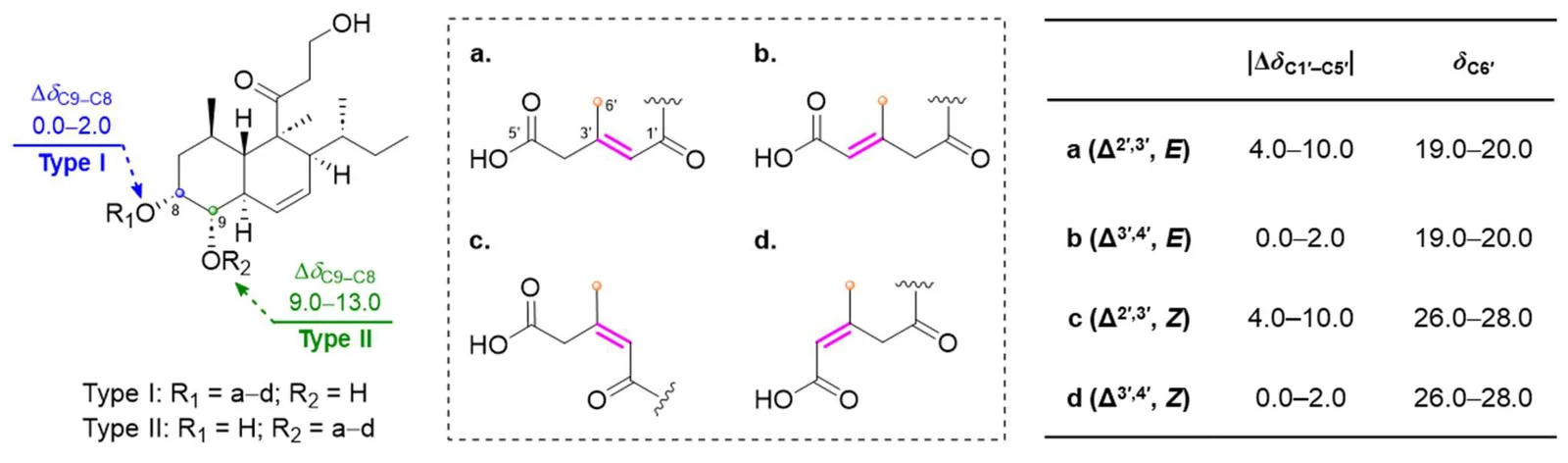
Schematic summary of empirical ^13^C NMR trends for side-chain assignment in tandyukisin-type decalin polyketides. The C8/C9, C1′/C5′, and C6′ chemical shifts support assignment of the side-chain attachment site, double-bond position, and *E/Z* geometry, respectively. The dashed box encloses the four representative side-chain variants (**a**–**d**). Δ*δ*_C8–C9_ represents the difference between C8 and C9 chemical shifts. |Δ*δ*_C1′–C5′_| represents the absolute difference between C1′ and C5′ chemical shifts. All chemical shifts are expressed in ppm.

Comparison of the ^13^C NMR data of twelve reported tandyukisin-type decalin polyketides revealed several empirical trends for side-chain assignment (Figure 2 and Table S2). As summarized in Figure 2, C8-*O*-acylated analogues (Type I) generally show small C8/C9 chemical-shift differences of 0.0–2.0 ppm, whereas C9-*O*-acylated analogues (Type II) display larger differences of 9.0–13.0 ppm. The side-chain variants (**a**–**d**) in Figure 2 summarize the combinations of double-bond position and geometry. The C6′ methyl resonance supports double-bond geometry, appearing at *δ*_C_ 19.0–20.0 ppm for *E* isomers and *δ*_C_ 26.0–28.0 ppm for *Z* isomers. In addition, Δ^2′,3′^ side chains generally show more shielded C1′ carbonyl resonances and larger C1′/C5′ chemical-shift differences of 4.0–10.0 ppm when C5′ is observed, whereas Δ^3′,4′^ side chains show a more downfield C1′ resonance and a smaller difference of 0.0–2.0 ppm. These chemical-shift patterns were used as qualitative supporting evidence, while definitive assignments were based primarily on HSQC, COSY, HMBC, and NOESY correlations.

**Figure 3:**
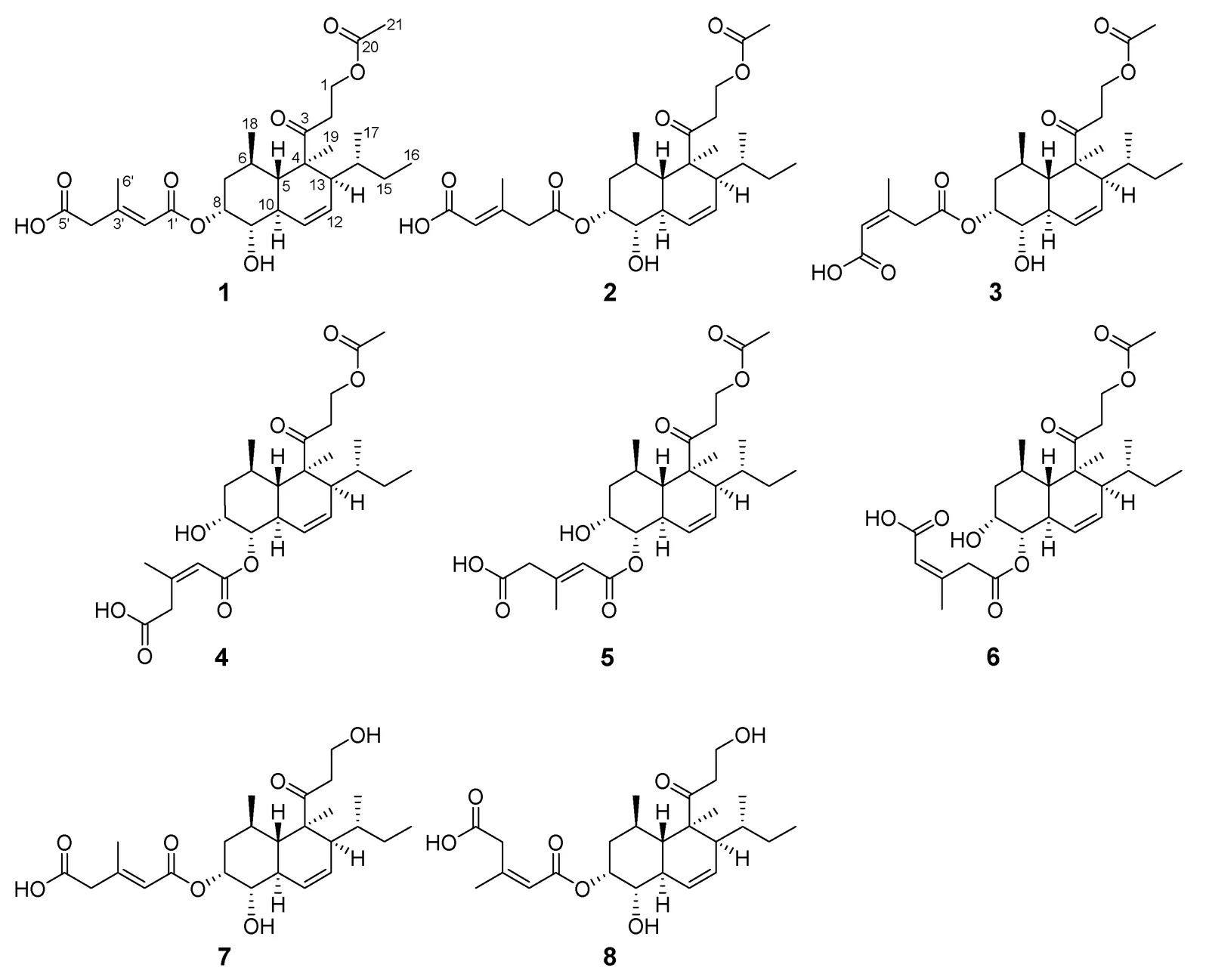
Compounds **1**–**8** isolated from fungus *T. longicollum* 17007.

**Figure 4:**
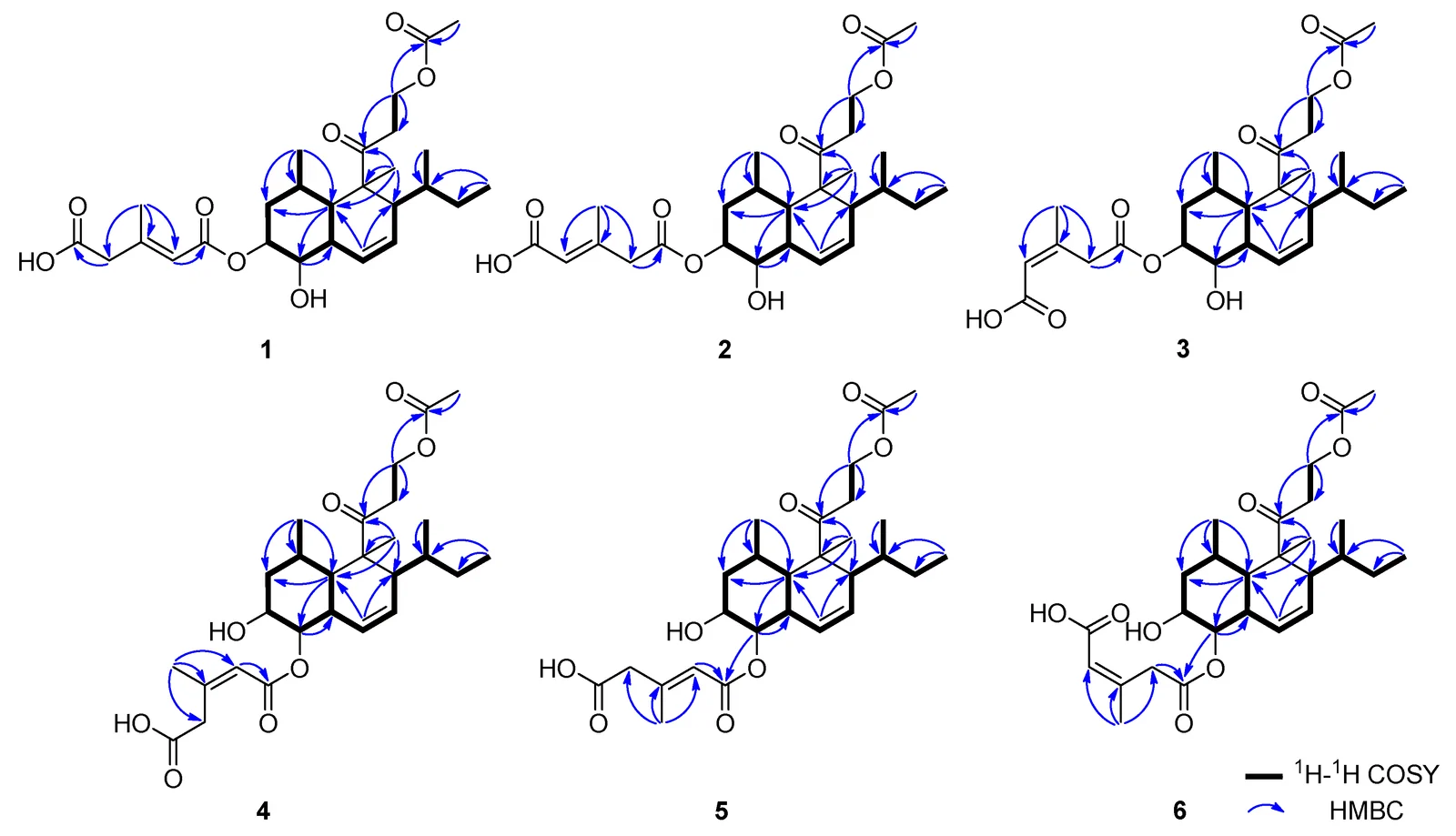
^1^H-^1^H COSY and HMBC correlations of new compounds **1**–**6**.

**Figure 5:**
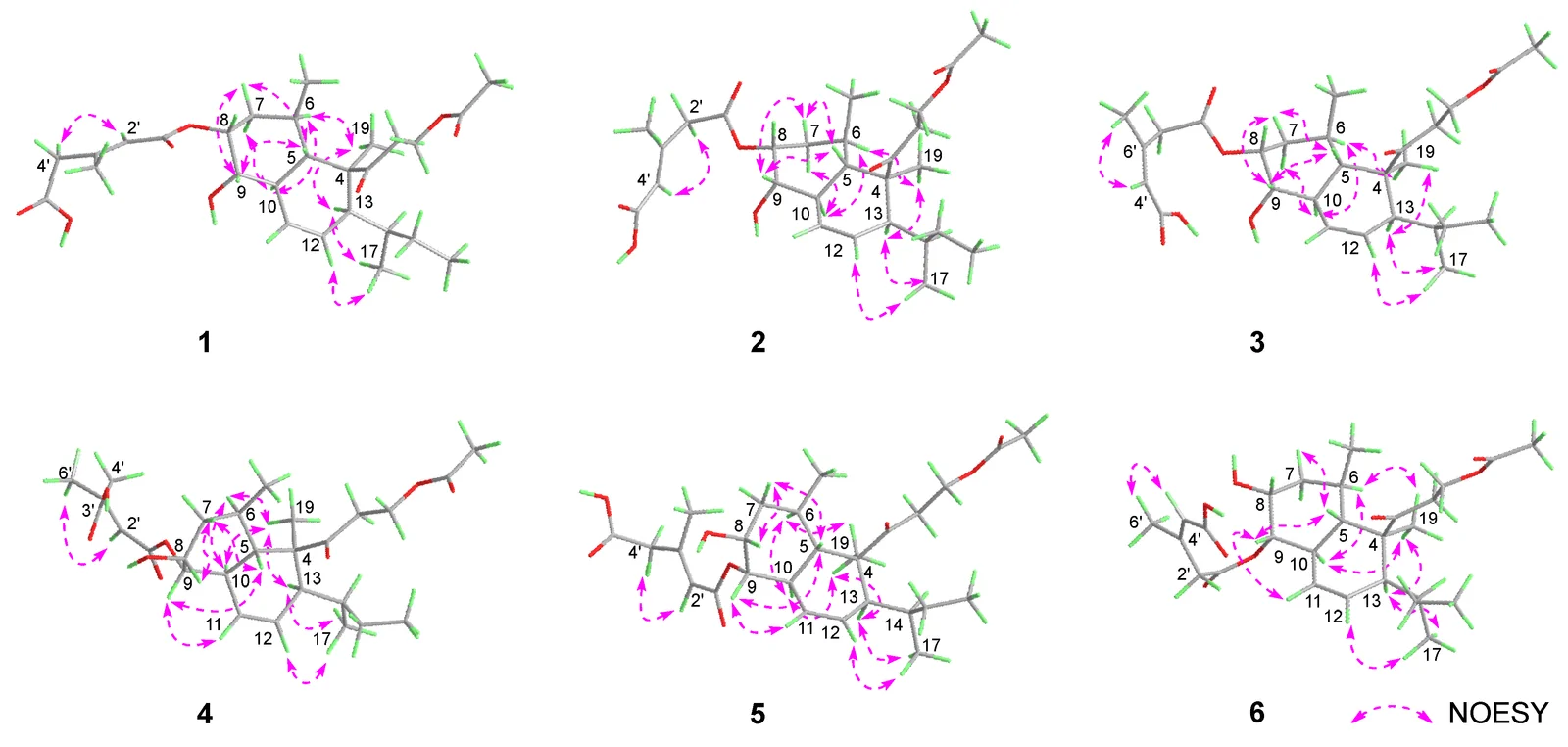
Key NOESY correlations of new compounds **1**–**6**. Carbon, oxygen, and hydrogen atoms are shown in gray, red, and green, respectively.

**Figure 6:**
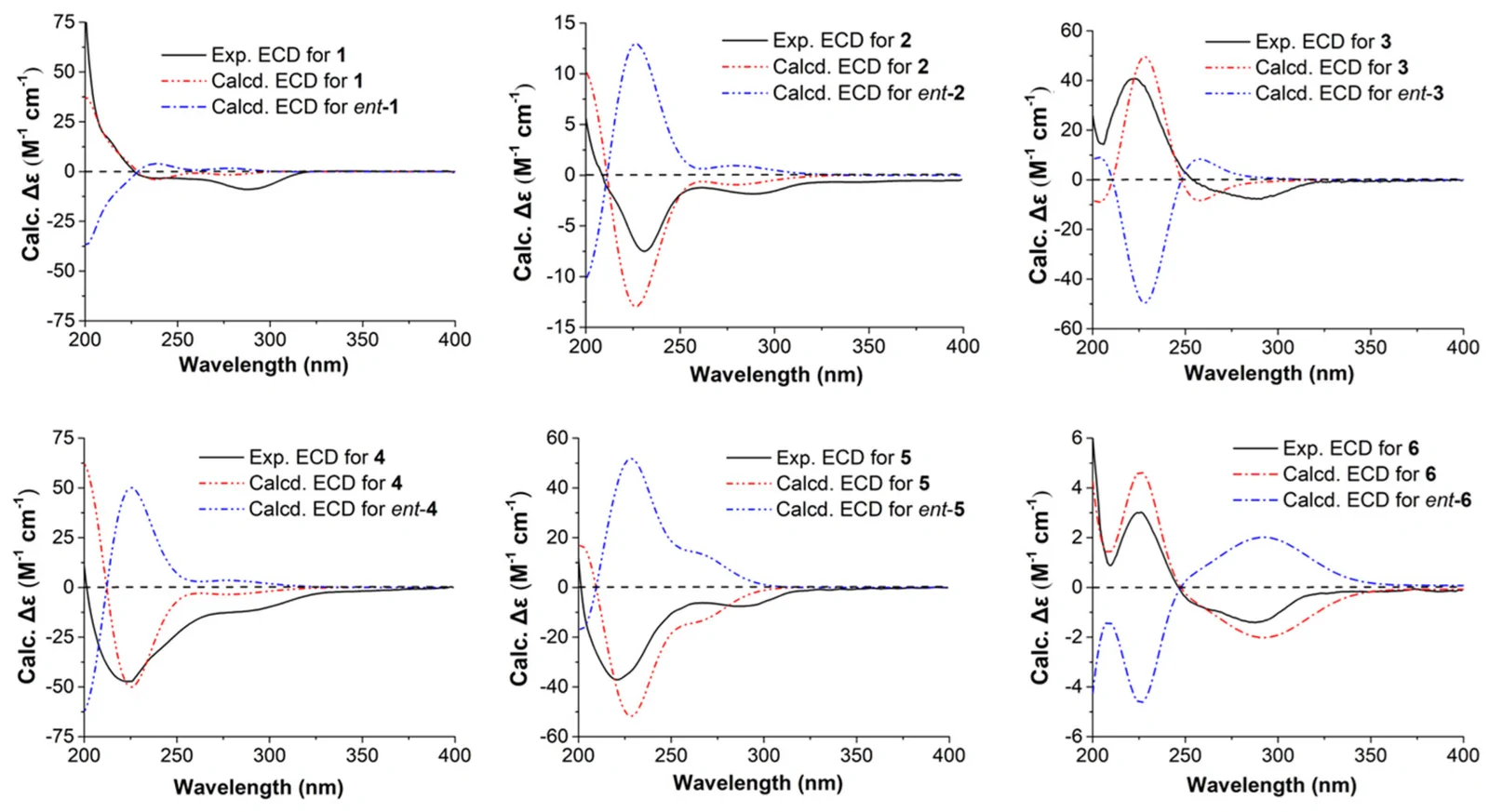
Experimental CD and computed ECD of compounds **1**–**6**.

Compound **1** was obtained as a colorless oil, and its molecular formula was revealed to be C_27_H_40_O_8_, as established from the [M + H]^+^ peak at *m*/*z* 493.2793 (*calcd*. for C_27_H_41_O_8_, 493.2796, Δ_error_ = 0.5 ppm) through HRESIMS (Figure S4a), implying eight degrees of unsaturation. The ^1^H NMR, ^13^C NMR, and HSQC spectra data of compound **1** revealed the following, respectively: four carbonyl carbons including one ketone (*δ*_C_ 213.1), two ester carbonyls (*δ*_C_ 167.7 and 172.8), and one carboxyl (*δ*_C_ 175.9); four olefinic carbons including three methines (*δ*_C_/*δ*_H_ 127.2/6.04, 124.6/5.72, 120.0/5.86) and one *sp^2^* quaternary carbon (*δ*_C_ 154.8); and two *sp^3^* quaternary carbons (*δ*_C_ 53.6 and 32.6), six saturated methines (*δ*_C_/*δ*_H_ 75.1/3.45, 73.7/5.21, 44.8/1.98, 41.1/2.21, 53.3/2.04, 38.0/1.21), five methylenes including one *O*-methene (*δ*_C_/*δ*_H_ 60.3/4.35 & 4.28, 39.2/2.98, 40.4/1.81 & 1.52, 25.8/1.52 & 0.75, 48.2/3.14), and six methyls (*δ*_C_/*δ*_H_ 22.7/0.57, 19.5/1.30, 19.5/0.96, 20.8/2.00, 19.4/2.23, 12.9/0.77) (Table 1 and Figure S4b–d). The NMR data of compound **1** closely resembled those of trichoharzin (**7**) (Figure 3, Table 1 and Table S3) [26], indicating that they share an identical decalin core structure, which was supported by further analysis of ^1^H-^1^H COSY and HMBC spectra (Figure 4 and Figure S4e–g). Compound **1** was distinguished by an additional acetyl group, as indicated by the NMR signals at *δ*_C_/*δ*_H_ 20.8/2.00, and *δ*_C_ 172.8. The HMBC correlations from H1 (*δ*_H_ 4.35 and 4.28) to C20 (*δ*_C_ 172.8) and H21 (*δ*_H_ 2.00) to C20 (*δ*_C_ 172.8) confirmed that the acetyl was connected at 1-OH. The similar ^13^C NMR chemical shifts in C8 (*δ*_C_ 73.7) and C9 (*δ*_C_ 75.1), corresponding to a difference of 1.4 ppm, supported attachment of the side chain at *O*-8, consistent with the empirical pattern observed for reported *O*-8-acylated tandyukisins (Figure 2 and Table S2). The presence of an olefinic methine at C2′ and a methylene at C4′, together with the relatively upfield C1′ carbonyl resonance (*δ*_C_ 167.7) compared with C5′ (*δ*_C_ 175.9), established a Δ^2′,3′^ double bond. The C1′/C5′ chemical-shift difference of 8.2 ppm was consistent with the 4.0–10.0 ppm range characteristic of reported Δ^2′,3′^ side chains (Figure 2 and Table S2). The *E* geometry was assigned from the NOESY correlation between H2′ and H4′ (Figure 5) and was further supported by the relatively upfield C6′ methyl resonance at *δ*_C_ 19.4, which falls within the characteristic range of 19.0–20.0 ppm for *E*-configured analogues (Figure 2 and Table S2). The relative stereochemistry of **1** was established by NOESY experiment (Figure 5). Correlations among H7*β*, H5, and H9 placed these protons on the same face of ring A, and supported a chair conformation with *β*-oriented H5 and H9. Together with the small vicinal coupling constant between H8 and H9 (*J*_8,9_ = 3.0 Hz), these data supported a *cis* relationship between the *O*-8 acyl substituent and 9-OH, with the *O*-8 substituent adopting an axial orientation [11,27]. Additional NOESY correlations involving H7*α*, H10, H6, and H19 placed these protons on the opposite α-orientation (Figure 5). Correlations of H6/H19, H19/H13, H13/H17, and H12/H17 further supported a half-chair conformation for ring B and established H13, 17-CH_3_, and 19-CH_3_ as *α*-oriented, with the sec-butyl substituent trans to 19-CH_3_. The relative configuration thus defined, together with the agreement between the experimental and TD-DFT-based ECD calculation (Figure 6), established the absolute configuration of **1** as 4*S*,5*S*,6*R*,8*R*,9*S*,10*R*,13*S*,14*R*. Comparison with previously reported tandyukisin-type polyketides indicated that *O*-acetylation at C1 had not been described previously (Figure S2) [10,11,26,27,28,29]. Compound **1** was therefore identified as a previously unreported C1-*O*-acetylated analogue of trichoharzin (**7**) [26], and named tandyukisin K (Figure 3).

Compound **2** was established to possess the molecular formula C_27_H_40_O_8_ on the basis of HR-ESI-MS data (*m*/*z* 515.2612 [M + Na]^+^, *calcd.* for C_27_H_40_O_8_Na^+^, 515.2615, Δ_error_ = 0.7 ppm), indicating eight degrees of unsaturation (Figure S5a). The molecular formula, ^1^H NMR, and ^13^C NMR data closely resembled those of compound **1** (Table 1, Figure S5b,c), except for minor differences in the side chain 3-methylpent-2-enedioate unit (C1′–C6′). HSQC and HMBC analyses identified C2′ as a methylene and C4′ as an olefinic methine, thereby establishing a Δ^3′,4′^ double bond (Figure 4 and Figure S5d–f). Moreover, downfield-shifted C1′ carbonyl resonance at *δ*_C_ 171.6, relative to that of **1** (*δ*_C_ 167.7), was consistent with reported Δ^3′,4′^ analogues, and this further supported this assignment (Table 1 and Table S2). The *E* geometry of the double bond was assigned from the NOESY correlation between H2′ and H4′ (Figure 5 and Figure S5g), and was further supported by the relatively upfield chemical shift of 6′-CH_3_ (*δ*_C_ 19.1), consistent with the empirical range of 19.0–20.0 ppm observed for *E*-configured analogues (Figure 2 and Table S2). The nearly identical C8 (*δ*_C_ 74.8) and C9 (*δ*_C_ 74.9) resonances supported attachment of the side chain at *O*-8. The absolute C8/C9 chemical-shift difference of 0.1 ppm was consistent with the empirical pattern observed for reported *O*-8-acylated tandyukisins (Figure 2 and Table S2). Further NOESY analysis indicated that the relative configuration of compound **2** was identical to that of **1** (Figure 5 and Figure S5g). The absolute configuration of **2** was determined based on ECD calculation results (Figure 6). Comparison with the reported tandyukisin G indicated that compound **2** possessed the same decalin scaffold and side-chain connectivity but contained an additional acetyl group at C1 [10]. Accordingly, compound **2** was identified as a previously unreported C1-*O*-acetylated analogue of tandyukisin G, and named tandyukisin L (Figure 3).

Compound **3** was assigned the molecular formula C_27_H_40_O_8_ on the basis of HR-ESI-MS data (*m*/*z* 515.2610 [M + Na]^+^, *calcd.* for C_27_H_40_O_8_Na^+^, 515.2615, Δ_error_ = 1.1 ppm), indicating eight degrees of unsaturation (Figure S6a). Compound **3** shared the molecular formula of **1** and displayed closely related ^1^H and ^13^C NMR profiles (Table 1, Figure S6b,c). The major difference was observed in the six-carbon unsaturated dicarboxylic acyl side chain. HSQC and HMBC analyses identified C2′ as a methylene and C4′ as an olefinic methine, thereby establishing a Δ^3′,4′^ double bond (Figure 4 and Figure S6d–f). The relatively downfield C1′ (*δ*_C_ 171.9) carbonyl resonance was consistent with reported Δ^3′,4′^ analogues and further supported this assignment (Figure 2 and Table S2). Its *Z* configuration was assigned based on the NOESY correlation between H4′/H6′ (Figure 5 and Figure S6g), and was further supported by the relatively downfield-shifted chemical shift of 6′-CH_3_ (*δ*_C_ 26.0), which falls within the 26.0–28.0 ppm range observed for reported *Z*-configured tandyukisins (Figure 2 and Table S2). The closely spaced ^13^C NMR resonances of C8 (*δ*_C_ 74.7) and C9 (*δ*_C_ 75.0) supported attachment of the side chain at *O*-8. The absolute C8/C9 chemical-shift difference of 0.3 ppm was consistent with the empirical pattern observed for reported *O*-8-acylated tandyukisins (Figure 2 and Table S2). NOESY analysis indicated that the relative configuration of compound **3** was identical to that of **1** (Figure 5 and Figure S6g). Further ECD computation confirmed the absolute configuration was 4*S*,5*S*,6*R*,8*R*,9*S*,10*R*,13*S*,14*R* (Figure 6). Comparison with the reported tandyukisin D indicated that compound **3** shared the same decalin scaffold, side-chain connectivity, and stereochemical configuration but contained an additional acetyl group at C1 [28]. Compound **3** was therefore identified as a previously unreported C1-*O*-acetylated analogue of tandyukisin D, and named tandyukisin M (Figure 3).

Compound **4** was established to possess the molecular formula C_27_H_40_O_8_ on the basis of HR-ESI-MS data (*m*/*z* 515.2612 [M + Na]^+^, *calcd.* C_27_H_40_O_8_Na^+^ for 515.2615, Δ_error_ = 0.7 ppm), indicating eight degrees of unsaturation (Figure S7a). The molecular formula, ^1^H NMR and ^13^C NMR are almost same as those of compound **1** (Table 2, Figure S7b,c), except for minor differences in the six-carbon unsaturated dicarboxylic acyl side chain. HSQC and HMBC analyses identified C2′ as an olefinic methine and C4′ as a methylene, thereby establishing a Δ^2′,3′^ double bond (Figure 4 and Figure S7d–f). The relatively shielded C1′ carbonyl resonance at *δ*_C_ 167.2 was consistent with reported Δ^2′,3′^ analogues and further supported this assignment (Figure 2 and Table S2). The *Z* configuration was assigned from the NOESY correlation between H2′/H6′ (Figure 5 and Figure S7g), and was further supported by the relatively downfield-shifted chemical shift of 6′-CH_3_ (*δ*_C_ 26.0), consistent with the empirical range of 26.0–28.0 ppm observed for *Z*-configured analogues (Figure 2 and Table S2). The pronounced difference between the C8 (*δ*_C_ 68.1) and C9 (*δ*_C_ 78.9), corresponding to a difference of 10.8 ppm, supported attachment of the side chain at *O*-9. This value falls within the empirical range 9.0–13.0 ppm range observed for reported C9-*O*-acylated tandyukisins (Figure 2 and Table S2). Further NOESY analysis indicated that the relative configuration of compound **4** was identical to that of **1** (Figure 5 and Figure S7g). The absolute configuration of **4** was determined based on ECD calculation results (Figure 6). Comparison with the C9-*O*-acylated tandyukisin J reported by Li et al. indicated that **4** otherwise shared the same decalin scaffold, side-chain connectivity, double-bond geometry, and stereochemical configuration [30], while bearing an additional acetyl group at C1. Compound **4** was therefore identified as a previously unreported C1-*O*-acetylated analogue of tandyukisin J, and named tandyukisin N (Figure 3).

Compound **5** was assigned the molecular formula C_27_H_40_O_8_ on the basis of HR-ESI-MS data (*m*/*z* 515.2612 [M + Na]^+^, *calcd.* for C_27_H_40_O_8_Na^+^, 515.2615, Δ_error_ = 0.6 ppm), indicating eight degrees of unsaturation (Figure S8a). Compound **5** had the same molecular formula as **1**, and its ^1^H and ^13^C NMR data closely resembled those of **1** (Table 2, Figure S8b,c). The principal difference was observed in the 3-methylpent-2-enedioate side-chain moiety (Figure 4 and Figure S8d–f). The presence of an olefinic methine at C2′ and a methylene at C4′, together with the relatively shielded C1′ carbonyl resonance at *δ*_C_ 167.2, established a Δ^2′,3′^ double bond. The absolute C1′/C5′ chemical-shift difference of 7.1 ppm was consistent with the larger differences observed for reported Δ^2′,3′^ analogues, further supporting this assignment (Figure 2 and Table S2). The *E* geometry was assigned based on the NOESY correlation between H2′/H4′ (Figure 5 and Figure S8g), and further supported by the relatively shielded chemical shift of 6′-CH_3_ (*δ*_C_ 19.3), consistent with the empirical range of 19.0–20.0 ppm observed for *E*-configured analogues (Figure 2 and Table S2). The attachment of the side chain at *O*-9 was confirmed by the key HMBC correlation from H9 to C1′ (Figure 4), and further supported by the pronounced difference between C8 (*δ*_C_ 68.1) and C9 (*δ*_C_ 78.8). The absolute C8/C9 chemical-shift difference of 10.7 ppm fell within the empirical range of 9.0–13.0 ppm observed for reported *O*-9-acylated tandyukisins (Figure 2 and Table S2). NOESY analysis indicated that the relative configuration of compound **5** was identical to that of **1** (Figure 5 and Figure S8g). Comparison of its experimental and ECD calculation spectra established the absolute configuration of **5** (Figure 6). Comparison with the reported tandyukisin I indicated that compound **5** shared the same decalin scaffold, *O*-9-linked side chain, double-bond geometry, and stereochemical configuration [10], but contained an additional acetyl group at C1. Compound **5** was therefore identified as a previously unreported C1-*O*-acetylated analogue of tandyukisin I, and named tandyukisin O (Figure 3).

Compound **6** was assigned the molecular formula C_27_H_40_O_8_ on the basis of HR-ESI-MS data (*m*/*z* 493.2802 [M + H]^+^, *calcd.* for C_27_H_41_O_8_^+^, 493.2796, Δ_error_ = 1.3 ppm), indicating eight degrees of unsaturation (Figure S9a). The molecular formula, as well as the ^1^H and ^13^C NMR spectra, were nearly identical to those of compound **1** (Table 2, Figure S9b,c), except for minor differences in the six-carbon unsaturated dicarboxylic acyl side chain. HSQC and HMBC analyses identified C2′ as a methylene and C4′ as an olefinic methine, thereby establishing a Δ^3′,4′^ double bond (Figure 4 and Figure S9d–f). The relatively downfield C1′ carbonyl resonance at *δ*_C_ 171.9 was consistent with reported Δ^3′,4′^ analogues and further supported this assignment (Figure 2 and Table S2). The *Z* geometry of the double bond was assigned from the NOESY correlation between H4′ and H6′ (Figure 5 and Figure S9g), together with the relatively downfield ^13^C chemical shift in the 6′-methyl group (*δ*_C_ 26.0), the empirical range of 26.0–28.0 ppm observed for *Z*-configured analogues (Figure 4 and Table S2). The side chain was attached at *O*-9 was established by the key HMBC correlation from H9 to C1′ and supported by the pronounced difference between C8 (*δ*_C_ 67.8) and C9 (*δ*_C_ 80.0) (Figure 4). The absolute C8/C9 chemical-shift difference of 12.2 ppm fell within the empirical range of 9.0–13.0 ppm observed for reported 9-*O*-acylated tandyukisins (Figure 2 and Table S2). Further NOESY analysis indicated that the relative configuration of compound **6** was identical to that of **1** (Figure 5 and Figure S9g). The absolute configuration of **6** was assigned by comparison of its experimental and ECD calculation spectra (Figure 6). Comparison with the reported tandyukisin C indicated that compound **6** shared the same decalin scaffold, side-chain connectivity, double-bond geometry, and stereochemical configuration [28], while bearing an additional acetyl group at C1. Compound **6** was therefore identified as a previously unreported C1-*O*-acetylated analogue of tandyukisin C, and named tandyukisin P (Figure 3).

Two known compounds, trichoharzin (**7**) and tandyukisin J (**8**), were also isolated from the fermentation products of *T. longicollum* 17007 and identified by comparison of their HR-MS, ^1^H NMR, and ^13^C NMR data with the reported values (Figures S10a–c and S11a–c, Table S3) [11,26]. It should be noted that two structurally different compounds have been reported under the name tandyukisin J. Compound **8** corresponds to the 8-*O*-acylated tandyukisin J reported by Yin et al. [11], whereas the compound reported under the same name by Li et al. is acylated at the C-9 hydroxy group [30].

### 2.4. Bioactivity Evaluation

Compounds **1**–**8** were evaluated for cytotoxicity against 11 human cell lines, including A549, MKN-45, HCT 116, HeLa, K-562, 786-O, TE-1, 5637, GBC-SD, L-02, and HL-60. All compounds showed IC_50_ values above 50 μM under the assay conditions (Table S4). In the antimycobacterial assay, tandyukisins K (**1**) and P (**6**) showed moderate activity against the avirulent *M. tuberculosis* H37Ra strain, both with a MIC of 12.5 μg/mL, whereas compounds **2**–**5**, **7**, and **8** showed MIC values above 50 μg/mL (Table S4).

### 2.5. Proposed Biosynthesis Pathway of Tandyukisins

**Figure 7:**
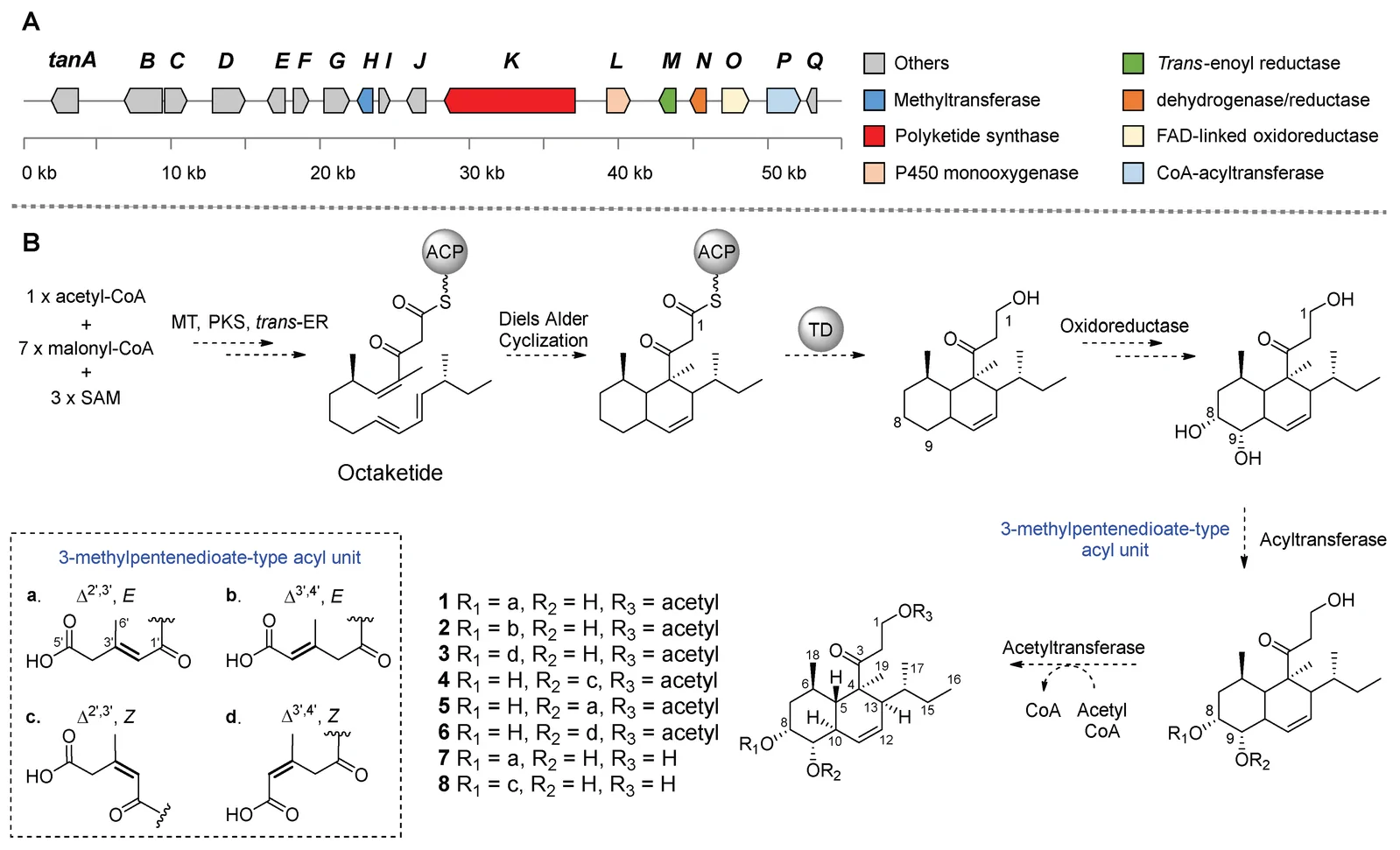
(**A**) Organization of the candidate *tan* biosynthetic gene cluster in *T. longicollum* 17007. (**B**) Proposed formation of compounds **1**–**8**. Dashed arrows indicate proposed biosynthetic transformations and putative enzyme classes that remain to be experimentally validated. MT, methyltransferase; PKS, polyketide synthase; *trans*-ER, trans-acting enoyl-reductase. The dashed box shows four representative side-chain variants (**a**–**d**) differing in double-bond position and geometry.

Based on the structural similarity of compounds **1**–**8** to fungal alkylated decalin polyketides, candidate biosynthetic gene clusters responsible for decalin formation were searched in *T. longicollum* 17007. CalA, a highly reducing polyketide synthase involved in calbistrin biosynthesis, and its trans-acting enoylreductase CalK were used as BLASTP queries [31,32]. Using a stringent cutoff of E-value < 1 × 10^−50^, region666.3 was identified as a candidate region containing both a CalA-like HR-PKS TanK and a CalK-like trans-ER TanM, which showed 61% and 64% similarity to CalA and CalK, respectively. This region was therefore designated as a putative tandyukisin biosynthetic gene cluster, namely the *tan* cluster (Figure 7A and Table S5). Based on the biosynthetic logic of fungal decalin polyketides, a tentative biosynthetic pathway was provided (Figure 7B). TanK and TanM, together with a methyltransferase, may participate in formation of the polyketide precursor and decalin scaffold [32,33], while neighboring oxidoreductases and acyltransferases may contribute to subsequent tailoring reactions [34,35]. The 3-methylpentenedioate-type acyl side chain may arise either from leucine catabolism or via an acetyl-CoA-derived route (Figure S12) [36,37,38]. However, the enzymes responsible for C1-acetylation remain unidentified. Further gene deletion, heterologous expression, isotope-labeled precursor feeding, and biochemical characterization will be required to validate the proposed pathway and the functions of the individual enzymes.

## 3. Discussion

This study highlights the value of FBMN-guided isolation for the targeted discovery of structurally related fungal secondary metabolites. Because structurally related metabolites often share similar MS/MS fragmentation patterns, FBMN is particularly useful for prioritizing minor or previously unreported analogues from complex extracts [19,22]. In this work, trichoharzin (**7**) was used as a seed compound to identify a decalin-related molecular-network cluster, which guided the isolation of six new tandyukisin-type polyketides, tandyukisins K–P (**1**–**6**), together with two known analogues.

Compounds **1**–**6** expand the structural diversity of tandyukisin-type decalin polyketides. Previously reported tandyukisins and trichoharzin share an oxygenated and alkylated decalin scaffold bearing a 3-methylpentenedioate-type acyl side chain [26,28]. The newly identified tandyukisins K–P retain this characteristic scaffold but are distinguished by C1-*O*-acetylation, a structural feature not previously reported for this compound family. Comparison with reported analogues showed that structural variation in this family is mainly associated with the *O*-8/*O*-9 esterification site of the 3-methylpentenedioate-type acyl side chain, the position and geometry of the side-chain double bond, terminal side-chain modification, and C1 substitution. In this study, we summarized several empirical ^13^C NMR trends associated with the side-chain variations. The C8/C9 chemical-shift difference helps distinguish *O*-8/*O*-9-acylated analogues, the C6′ methyl resonance supports *E*/*Z* assignment, and the C1′ carbonyl resonance together with the C1′/C5′ chemical-shift difference assists in distinguishing Δ^2′,3′^ and Δ^3′,4′^ side chains. These trends are consistent with changes in the local electronic and conformational environments around the oxygenated C8/C9 region and the unsaturated acyl side chain. The compounds obtained in this study follow these empirical trends, supporting their use as practical references for preliminary assignment of future tandyukisin-type analogues, although definitive structure determination should still rely on 2D NMR correlations.

Biological evaluation showed that tandyukisins K (**1**) and P (**6**) exhibited moderate activity against the attenuated *M*. *tuberculosis* H37Ra strain, with MIC values of 12.5 μg/mL, whereas compounds **2**–**5**, **7**, and **8** had MIC values of >50 μg/mL. All compounds showed cytotoxic IC_50_ values above 50 μM against the tested human cell lines. Regarding the antimycobacterial activity, no clear structure–activity relationship could be established from the current compound set. The two active compounds **1** and **6** differ in the attachment site, double-bond position, and geometry of the 3-methylpentenedioate-type side chain, while related structural features are also present among the inactive analogues. This suggests that the observed antimycobacterial activity may be influenced by the combined effects of these structural features rather than by a single structural variation. To the best of our knowledge, this is the first report of activity against *M. tuberculosis* H37Ra within the tandyukisin-type decalin polyketide family. Further evaluation against virulent *M. tuberculosis* H37Rv and drug-resistant clinical isolates will be necessary to assess the biological significance of these compounds.

Genome mining identified region666.3 as a candidate *tan* biosynthetic gene cluster containing homologues of the HR-PKS/trans-ER pair associated with fungal decalin biosynthesis [32]. Although this gene organization is consistent with a possible role in the formation of the polyketide precursor and decalin scaffold, the cluster–metabolite relationship and the functions of the individual enzymes have not been experimentally established. In particular, the enzyme responsible for C1-*O*-acetylation has not been identified and may be encoded outside the proposed cluster. Accordingly, the *tan* pathway remains a bioinformatics-guided hypothesis pending genetic and biochemical validation.

Overall, this study expands the structural diversity of tandyukisin-type decalin polyketides by identifying six new C1-acetylated analogues from *T. longicollum* 17007. More importantly, it reveals previously unrecognized antitubercular activity in this compound family. These findings support further FBMN-guided exploration of *Trichoderma* species as sources of new antimycobacterial natural products.

## 4. Materials and Methods

### 4.1. General Experimental Procedures

Spectroscopic data were gathered on a 600 MHz Agilent DD2 NMR system (Agilent Technologies, Santa Clara, CA, USA), designated for both ^1^H and ^13^C analyses with CD_3_OD and CDCl_3_ as the solution. LC–HRMS data were acquired in ESI mode using a Thermo Q Exactive Orbitrap mass spectrometer (Thermo Fisher Scientific, Bremen, Germany) coupled to a Shimadzu LC-30AD UPLC system (Shimadzu Corporation, Kyoto, Japan) equipped with a Waters ACQUITY BEH C18 column (Waters Corporation, Milford, MA, USA) (2.1 × 100 mm, 1.7 μm). Routine LC–MS analysis was performed on an Agilent 1100 Series LC system connected to an Agilent G1956B mass detector (Agilent Technologies, Santa Clara, CA, USA). Thin-layer chromatography (TLC) was performed on silica gel plates purchased from Merck (Merck KGaA, Darmstadt, Germany). TLC spots were visualized under a 254 nm and 365 nm UV light. Silica gels (200–300 mesh and 300–400 mesh) used for normal-phase column chromatography were obtained from Titan Scientific (Shanghai Titan Scientific Co., Ltd., Shanghai, China). Sephadex LH-20 (GE Healthcare Bio-Sciences AB, Uppsala, Sweden) were used for purification. Specific rotation measurements were conducted on a Perkin Elmer Model 343 polarimeter (PerkinElmer, Waltham, MA, USA) with samples in methanol solution. CD spectra were recorded in MeOH on a Chirascan circular dichroism spectrometer (Applied Photophysics Ltd., Leatherhead, Surrey, UK). HPLC was conducted utilizing ACE C18 column (Avantor, Radnor, PA, USA) (4.6 × 250 mm, 5 μm) and Cosmosil Cholester column (Nacalai Tesque, Inc., Kyoto, Japan) (4.6 × 250 mm, 5 μm). For semipreparative HPLC, ACE C18 column (10 × 250 mm, 5 μm) and Cosmosil Cholester column (10 × 250 mm, 5 μm) were employed. Biological reagents, chemicals, and media were purchased from standard commercial sources unless stated.

### 4.2. Microbial Strain Culture, Identification, Genome Sequencing, and Bioinformatic Analysis

Fungal strain *T. longicollum* 17007 was isolated from a wood sample collected in Wuhan, Hubei Province, China. This strain has been deposited in the China General Microbiological Culture Collection Centre (accession no. CGMCC No.40622) and stored at −80 °C. For morphological observation, the strain was cultured on potato dextrose agar (glucose 20 g/L, potato extract 4 g/L, and agar 15 g/L) at 28 °C for 10 days. Genomic DNA was extracted from the harvested mycelia using the cetyltrimethylammonium bromide (CTAB) method [39,40]. The internal transcribed spacer (ITS) region was amplified using the forward primer (F: 5′-GGAAGTAAAAGTCGTAACAAGG-3′) and the reverse primer (R: 5′-TCCTCCGCTTATTGATATGC-3′) [41]. The obtained ITS sequence was compared with reference sequences retrieved from the NCBI nucleotide database. A neighbor-joining phylogenetic tree was constructed using MEGA 11 [42,43], and branch support was assessed using 1000 bootstrap replicates. Whole-genome sequencing and assembly of *T. longicollum* 17007 were performed according to a previously described protocol [44,45]. Biosynthetic gene clusters were predicted using antiSMASH 8.0 [46]. Secondary-metabolism-related proteins were additionally surveyed using the 2ndFind web server (https://biosyn.nih.go.jp/2ndfind/, accessed on 5 June 2026). Putative protein functions were assigned using BLASTp (BLAST+ v2.17.0) searches against the NCBI nonredundant protein database and were manually curated based on characterized homologues [47].

### 4.3. LC-MS/MS Analysis and Molecular Network Analysis

The MeOH solution of *T. longicollum* 17007 extract was prepared (1 mg/mL) and analysis was conducted using a Full-MS/dd-MS^2^ acquisition method. LC–MS/MS analysis was performed on a Shimadzu LC-30AD UPLC system connected to a Thermo Scientific Q Exactive Orbitrap mass spectrometer equipped with a heated electrospray ionization (HESI) source operating in positive ion mode. Chromatographic separation was carried out on a Waters ACQUITY BEH C18 column (150 mm × 4.6 mm, 1.7 μm) using acidified water and acetonitrile as the mobile phases. The mobile phase consisted of solvent A, water containing 0.1% formic acid, and solvent B, acetonitrile containing 0.1% formic acid. The LC gradient was as follows: 0.00–14.00 min, 30–45% B; 14.00–17.00 min, 45–99% B; 17.10–20.00 min, 30% B for column re-equilibration. The flow rate was 0.35 mL/min, the injection volume was 10 μL, and the column temperature was maintained at 45 °C. PDA data were collected over a wavelength range of 190–400 nm.

The MS workflow involved a Full-MS scan at a resolution of 70,000 within the *m*/*z* range of 133.7 to 2000 Da, with an AGC target of 3 × 10^6^ and a maximum injection time of 100 ms. The dd-MS^2^ acquisition was performed at a resolution of 17,500, with an AGC target of 1 × 10^5^, a maximum injection time of 50 ms, and an isolation window of 1.0 *m*/*z*. The top five most intense precursor ions were selected for HCD fragmentation with a collision energy of 25 eV. The minimum AGC target and intensity threshold were 8 × 10^3^ and 1.6 × 10^5^, respectively. No charge exclusion was applied; isotope exclusion was enabled, and dynamic exclusion was set to 10.0 s. The HESI source parameters were as follows: spray voltage, 3.5 kV; sheath gas flow rate, 30 arbitrary units; auxiliary gas flow rate, 10 arbitrary units; capillary temperature, 320 °C; probe heater temperature, 210 °C; and S-lens RF level, 50%.

The LC-MS/MS data with *.raw format, natively for the Thermo instrument, was converted to GNPS-compatible *.mzML format using MSConvert (v3.0.4738) [48]. Analysis of the *.mzML format data was conducted with MZmine (v2.53) [49]. The mass detections were performed with noise levels at 1.0E6 for MS1 and 1.0E3 for MS2. The ADAP chromatogram building used a minimum time span of 0.1 min, *m*/*z* tolerance of 0.01 (or 10 ppm), and minimum height of 3.0E6. Deisotope was carried out utilizing isotopic peaks grouper algorithm, with a RT tolerance of 0.1 min and *m*/*z* tolerance of 0.01 (or 10 ppm). Duplicate peaks were filtered with filter mode of old average, *m*/*z* tolerance of 0.01 (or 10 ppm), and retention time (RT) range = 2 min. Then the *.mgf file and *.mzML file were subsequently uploaded to GNPS and processed with the Feature-based molecular networking (FBMN) workflow (version release_28.2) [19,22]. Cytoscape (version 3.7.2) was used to visualize the corresponding output FBMN data [50].

### 4.4. Strain Cultivation, Extraction and Compounds Isolation

*T*. *longicollum* 17007 was inoculated onto PDA and incubated at 28 °C for 5 days. Agar plugs were then transferred to 100 mL of potato dextrose broth (PDB) and cultured with shaking (28 °C, 220 rpm) for 5 days to prepare the seed culture. Solid-state fermentation was carried out using a rice-based medium, which was prepared in 400 cultivation bags, each containing rice (80 g) and distilled water (120 mL), and sterilized by autoclaving at 121 °C for 30 min. After inoculation with the seed culture, the bags were incubated at 28 °C for 35 days. A total of 35 kg of fermentation culture was extracted with ethyl acetate (EtOAc), and the pooled EtOAc extracts were evaporated under reduced pressure to afford a crude extract (130 g).

The crude extract was suspended in water and sequentially partitioned with petroleum ether, EtOAc, and water to obtain fractions E1 (40 g), E2 (75 g), and E3 (5 g). Fraction E2 (75 g) was purified on a silica gel column with a CH_2_Cl_2_/MeOH gradient (*v*/*v*, 100:0, 99:1, 98:2, 97:3, 96:4, 0:100) as the eluent to yield six fractions (E2G1−E2G6). Fractions E2G1−E2G3 were merged (E2G_1–3_, 37 g) and further separated by Sephadex LH-20 elution with 100% MeOH to obtain twenty-five fractions (E2G_1–3_N1−E2G_1–3_N25). Fraction E2G_1–3_N12 (302.4 mg) was further purified by semipreparative HPLC (ACE C18-AR, 10 × 250 mm, 5 μm; 4 mL/min, 60% ACN/H_2_O) to obtain compounds **2** (6.3 mg, *t*_R_ = 22.5 min), **3** (5.8 mg, *t*_R_ = 14.1 min), **4** (21.0 mg, *t*_R_ = 18.5 min), and **6** (2.5 mg, *t*_R_ = 15.1 min). Fraction E2G_1–3_N13 (108.9 mg) was purified by semipreparative HPLC (ACE C18-PFP, 10 × 250 mm, 5 μm; 4 mL/min, 55% ACN/H_2_O) to obtain compounds **1** (1.5 mg, *t*_R_ = 26.3 min), and **5** (14.7 mg, *t*_R_ = 22.3 min). Fraction E2G_1–3_N14 (387.6 mg) was purified by semipreparative HPLC (ACE C18, 10 × 250 mm, 5 μm; 4 mL/min, 50% MeOH/H_2_O) to obtain compounds **7** (3.4 mg, *t*_R_ = 8.2 min), and **8** (2.8 mg, *t*_R_ = 6.7 min).

Tandyukisin K (**1**): colorless oil; [*α*${]}_{D}^{30}$ +91.08 (*c* 0.10, MeOH); HR-ESI-MS: *m*/*z* 493.2793 [M + H]^+^ (*calcd*. for C_27_H_41_O_8_^+^, *m*/*z* 493.2796) (Figure S4a); ^1^H NMR and ^13^C-NMR data, see Table 1, and Figure S4b–g.

Tandyukisin L (**2**): colorless oil; [*α*${]}_{D}^{30}$ +64.70 (*c* 0.10, MeOH); HR-ESI-MS: *m*/*z* 515.2612 [M + Na]^+^ (*calcd*. for C_27_H_40_O_8_Na^+^, *m*/*z* 515.2615) (Figure S5a); ^1^H NMR and ^13^C-NMR data, see Table 1, and Figure S5b–g.

Tandyukisin M (**3**): colorless oil; [*α*${]}_{D}^{30}$ +59.50 (*c* 0.10, MeOH); HR-ESI-MS: *m*/*z* 515.2610 [M + Na]^+^ (*calcd*. for C_27_H_40_O_8_Na^+^, *m*/*z* 515.2615) (Figure S6a); ^1^H NMR and ^13^C-NMR data, see Table 1, and Figure S6b–g.

Tandyukisin N (**4**): colorless oil; [*α*${]}_{D}^{30}$ +109.12 (*c* 0.10, MeOH); HR-ESI-MS: *m*/*z* 515.2612 [M + Na]^+^ (*calcd*. for C_27_H_40_O_8_Na^+^, *m*/*z* 515.2615) (Figure S7a); ^1^H NMR and ^13^C-NMR data, see Table 2, and Figure S7b–g.

Tandyukisin O (**5**): colorless oil; [*α*${]}_{D}^{30}$ +60.39 (*c* 0.10, MeOH); HR-ESI-MS: *m*/*z* 515.2612 [M + Na]^+^ (*calcd*. for C_27_H_40_O_8_Na^+^, *m*/*z* 515.2615) (Figure S8a); ^1^H NMR and ^13^C-NMR data, see Table 2, and Figure S8b–g.

Tandyukisin P (**6**): colorless oil; [*α*${]}_{D}^{30}$ +81.60 (*c* 0.10, MeOH); HR-ESI-MS: *m*/*z* 493.2802 [M + H]^+^ (*calcd*. for C_27_H_41_O_8_^+^, *m*/*z* 493.2796) (Figure S9a); ^1^H NMR and ^13^C-NMR data, see Table 2, and Figure S9b–g.

Trichoharzin (**7**): colorless oil; HR-ESI-MS: *m*/*z* 473.2502 [M + Na]^+^ (*calcd*. for C_25_H_38_O_7_Na, *m*/*z* 473.2510) (Figure S10a); ^1^H NMR and ^13^C-NMR data, see Table S3, Figure S10b,c.

Tandyukisin J (**8**): colorless oil; HR-ESI-MS: *m*/*z* 473.2505 [M + Na]^+^ (*calcd*. for C_25_H_38_O_7_Na, *m*/*z* 473.2510) (Figure S11a); ^1^H NMR and ^13^C-NMR data, see Table S3, Figure S11b,c.

### 4.5. Density Functional Theory Calculations

Electronic circular dichroism (ECD) calculations were performed using the Gaussian 09 program package [51]. Conformational searches were performed using SYBYL-X 2.0, and the resulting low-energy conformers were optimized at the B3LYP/6-31G(d) level with the polarizable continuum model (PCM) for methanol [52,53,54]. Time-dependent density functional theory (TD-DFT) calculations were then performed at the B3LYP/6-31G(d,p) level with PCM for methanol to obtain the electronic excitation energies and rotational strengths [54,55,56]. The calculated ECD spectra were compared with the experimental spectra to assign the absolute configurations of compounds **1**–**6**.

### 4.6. Biological Assay

The antimycobacterial activity of compounds **1**–**8** was evaluated against *M. tuberculosis* H37Ra using a modified microdilution method in 96-well plates following a previously reported protocol [57]. Compounds were tested by two-fold serial dilution up to 50 μg/mL, with isoniazid as the positive control. Minimum inhibitory concentrations (MICs) were defined here as the lowest concentration that inhibited more than 90% of bacterial growth assessed by fluorescence value. The assays were performed in three independent experiments, with triplicate wells per concentration.

The cytotoxicity of compounds **1**–**8** was evaluated against A549, MKN-45, HCT 116, HeLa, K-562, 786-O, TE-1, 5637, GBC-SD, L-02, and HL-60 cells using the CCK-8 assay following a previously reported method [58,59]. Compounds were tested at concentrations up to 50 μM, with doxorubicin hydrochloride as the positive control. Cell viability was calculated relative to the DMSO-treated control. The assays were performed in three independent experiments, with triplicate wells per concentration.

## 5. Conclusions

FBMN-guided isolation led to the discovery of six new tandyukisin-type decalin polyketides, tandyukisins K–P (**1**–**6**), from *T. longicollum* 17007. These compounds are C1-*O*-acetylated analogues bearing a 3-methylpentenedioate-type acyl side chain, thereby expanding the structural diversity of this compound family. Comparative ^13^C NMR analysis provided practical empirical references for assigning side-chain attachment site, double-bond geometry, and double-bond position in tandyukisin-type analogues. Tandyukisins K (**1**) and P (**6**) showed moderate activity against the avirulent *M. tuberculosis* H37Ra both with a MIC value of 12.5 μg/mL. These results reveal previously unrecognized antitubercular activity in tandyukisin-type decalin polyketides and support further exploration of *Trichoderma* metabolites as potential antimycobacterial natural products.

## 6. Patents

The compounds and preparation methods described in this manuscript are the subject of a published Chinese patent application entitled “Decalin Derivatives Derived from a Plant Endophytic Fungus, and Preparation Methods and Uses Thereof” (application No. CN202311145602.1; publication No. CN117417255A).

## Figures and Tables

**Table 1:** ^1^H and ^13^C NMR Data of compounds **1**–**3** in methanol-*d*_4_.

Pos.	1 ^a^		2 ^a^		3 ^a^	
	*δ*_C_, Type	*δ*_H_, Mult (*J* in Hz)	*δ*_C_, Type	*δ*_H_, Mult (*J* in Hz)	*δ*_C_, Type	*δ*_H_, Mult (*J* in Hz)
1*α* 1*β*	60.3, CH_2_	4.35, m 4.28, m	60.3, CH_2_	4.36, m 4.30, m	60.3, CH_2_	4.35, m 4.30, m
2	39.2, CH_2_	2.98, m	39.2, CH_2_	2.98, m	39.2, CH_2_	2.98, m
3	213.1, C		213.0, C		213.0, C	
4	53.6, C		53.7, C		53.7, C	
5	44.8, CH	1.98, m	44.8, CH	1.99, m	44.8, CH	1.98, m
6	32.6, C	1.65, brs	32.6, C	1.65, brs	32.6, C	1.65, m
7*α* 7*β*	40.4, CH_2_	1.81, m 1.52, m	40.3, CH_2_	1.80, m 1.52, m	40.3, CH_2_	1.80, m 1.49, m
8	73.7, CH	5.21, m	74.8, CH	5.20, m	74.7, CH	5.19, m
9	75.1, CH	3.45, dd (10.9, 3.0)	74.9, CH	3.47, dd (10.9, 3.0)	75.0, CH	3.44, dd (10.9, 3.0)
10	41.1, CH	2.21, m	41.1, CH	2.22, m	41.2, CH	2.19, t (10.7)
11	127.2, CH	6.04, d (10.7)	127.2, CH	6.05, d (10.7)	127.3, CH	6.05, d (10.7)
12	124.6, CH	5.72, m	124.7, CH	5.74, m	124.7, CH	5.73, m
13	53.3, CH	2.04, m	53.4, CH	2.05, brs	53.3, CH	2.05, m
14	38.0, CH	1.21, m	38.0, CH	1.22, m	38.0, CH	1.21, m
15*α* 15*β*	25.8, CH_2_	1.52, m 0.75, m	25.8, CH_2_	1.53, m 0.73, m	25.8, CH_2_	1.53, m 0.74, m
16	12.9, CH_3_	0.77, t (6.8)	12.9, CH_3_	0.78, t (6.8)	12.9, CH_3_	0.77, t (6.8)
17	19.5, CH_3_	0.96, d (6.9)	19.4, CH_3_	0.98, d (6.8)	19.5, CH_3_	0.97, d (6.9)
18	22.7, CH_3_	0.57, d (6.9)	22.7, CH_3_	0.58, d (6.8)	22.7, CH_3_	0.57, d (6.9)
19	19.5, CH_3_	1.30, s	19.4, CH_3_	1.30, s	19.5, CH_3_	1.30, s
20	172.8, C		172.7, C		172.7, C	
21	20.8, CH_3_	2.00, s	20.8, CH_3_	2.01, s	20.8, CH_3_	2.01, s
1′	167.7, C		171.6, C		171.9, C	
2′	120.0, CH	5.86, s	46.7, CH_2_	3.25, m	39.8, CH_2_	3.76, d (15.5)
3′	154.8, C		151.0, C		151.9, C	
4′	48.2, CH_2_	3.14, s	121.8, CH	5.83, s	121.0, CH	5.88, brs
5′	175.9		ND ^b^		ND ^b^	
6′	19.4, CH_3_	2.23, s	19.1, CH_3_	2.20, s	26.0, CH_3_	2.01, s

^a^ Recorded in methanol-*d*_4_, ^1^H 600 MHz, ^13^C 150 MHz; ^b^ Not detected.

**Table 2:** ^1^H and ^13^C NMR Data of compounds **4**–**6** in methanol-*d*_4_.

Pos.	4 ^a^		5 ^a^		6 ^a^	
	*δ*_C_, Type	*δ*_H_, Mult (*J* in Hz)	*δ*_C_, Type	*δ*_H_, Mult (*J* in Hz)	*δ*_C_, Type	*δ*_H_, Mult (*J* in Hz)
1*α* 1*β*	60.3, CH_2_	4.35, m 4.29, m	60.3, CH_2_	4.35, m 4.29, m	60.3, CH_2_	4.36, m 4.30, m
2	39.2, CH_2_	2.97, m	39.2, CH_2_	2.97, m	39.2, CH_2_	2.99, m
3	212.8, C		212.8, C		212.8, C	
4	53.6, C		53.6, C		53.6, C	
5	45.2, CH	2.05, m	45.3, CH	2.05, m	45.2, CH	2.03, m
6	31.6, C	1.80, m	31.6, C	1.80, m	31.6, C	1.80, m
7*α* 7*β*	42.8, CH_2_	1.78, m 1.52, m	42.9, CH_2_	1.78, m 1.52, m	42.7, CH_2_	1.78, m 1.51, m
8	68.1, CH	4.09, m	68.1, CH	4.09, m	67.8, CH	4.13, m
9	78.9, CH	4.63, dd (10.9, 3.0)	78.8, CH	4.63, dd (10.9, 3.0)	80.0, CH	4.58, dd (10.9, 3.0)
10	37.5, CH	2.49, t (10.7)	37.5, CH	2.49, m	37.4, CH	2.49, t (10.7)
11	126.5, CH	5.65, d (10.7)	126.4, CH	5.65, m	126.4, CH	5.72, m
12	125.3, CH	5.70, m	125.4, CH	5.70, m	125.4, CH	5.72, m
13	53.3, CH	2.05, m	53.3, CH	2.04, m	53.3, CH	2.05, m
14	38.0, CH	1.21, m	38.0, CH	1.21, m	38.0, CH	1.21, m
15*α* 15*β*	25.7, CH_2_	1.53, m 0.75, m	25.7, CH_2_	1.53, m 0.75, m	25.7, CH_2_	1.52, m 0.75, m
16	12.9, CH_3_	0.78, t (6.8)	12.9, CH_3_	0.78, t (6.8)	12.8, CH_3_	0.78, t (6.8)
17	19.5, CH_3_	0.96, d (6.9)	19.4, CH_3_	0.95, d (7.0)	19.4, CH_3_	0.97, d (6.9)
18	22.8, CH_3_	0.60, d (6.9)	22.8, CH_3_	0.60, d (6.9)	22.8, CH_3_	0.60, d (6.9)
19	19.4, CH_3_	1.32, s	19.5, CH_3_	1.30, s	19.4, CH_3_	1.31, s
20	172.7, C		172.7, C		172.7, C	
21	20.8, CH_3_	2.01, s	20.8, CH_3_	1.99, s	20.8, CH_3_	2.01, s
1′	167.2, C		167.2, C		171.9, C	
2′	119.4, CH	5.96, s	120.0, CH	5.92, brs	39.4, CH_2_	3.80, m
3′	154.8, C		154.7, C		151.9, C	
4′	40.4, CH_2_	3.75, m	47.0, CH_2_	3.20, brs	121.2, CH	5.91, s
5′	ND ^b^		174.3, C		ND ^b^	
6′	26.0, CH_3_	2.02, d (1.0)	19.3, CH_3_	2.25, d (1.0)	26.1, CH_3_	2.02, s

^a^ Recorded in methanol-*d*_4_, ^1^H NMR 600 MHz, ^13^C NMR 150 MHz; ^b^ Not detected.

## Data Availability

The candidate *tan* biosynthetic gene cluster from *T. longicollum* 17007. has been deposited in GenBank under accession number PZ787232. The original contributions presented in this study are included in the article and Supplementary Materials. Further inquiries can be directed to the corresponding authors.

## Supplementary Materials

The following supporting information can be downloaded at https://www.mdpi.com/article/10.3390/antibiotics15080789/s1: *Trichoderma longicollum* 17007 ITS sequence; Figure S1: Identification of strain *Trichoderma* sp. 17007; Figure S2: LC-DAD-HRMS analysis of unidentified metabolites in the decalin fraction E2G_1–3_; Figure S3: The structure of known tandyukisin-type decalin polyketides; Figure S4a–g: HR-ESI-MS and NMR spectra of tandyukisin K (**1**); Figure S5a–g: HR-ESI-MS and NMR spectra of tandyukisin L (**2**); Figure S6a–g: HR-ESI-MS and NMR spectra of tandyukisin M (**3**); Figure S7a–g: HR-ESI-MS and NMR spectra of tandyukisin N (**4**); Figure S8a–g: HR-ESI-MS and NMR spectra of tandyukisin O (**5**); Figure S9a–g: HR-ESI-MS and NMR spectra of tandyukisin P (**6**); Figure S10a–c: HR-ESI-MS and 1D NMR spectra of trichoharzin (**7**); Figure S11a–c: HR-ESI-MS and 1D NMR spectra of tandyukisin J (**8**); Figure S12: Putative origins of the 3-methylpentenedioate-type acyl unit; Table S1: Annotations of the 23 nodes in the decalin cluster I from the *T. longicollum* 17007 feature-based molecular networking; Table S2: Comparative ^13^C NMR features and side-chain assignments of reported tandyukisin-type decalin polyketides; Table S3: ^1^H and ^13^C NMR Data of trichoharzin (**7**) and tandyukisin J (**8**) in CDCl_3_; Table S4: In vitro bioactivity evaluation of compounds **1**–**8**; Table S5: Deduced functions of ORFs in tandyukisins (*tan*) BGC from *T. longicollum* 17007 based on top BLASTP matches.
