# Supplementary material for: Tandyukisins K–P, New Decalin Polyketides from Trichoderma longicollum 17007 with Antitubercular Activity Against Mycobacterium tuberculosis H37Ra
Source: Antibiotics (Basel). 2026 Aug 14;15(8):789. doi: 10.3390/antibiotics15080789 (PMC13509314; doi:10.3390/antibiotics15080789)
Supplement: Supplementary file 1 [file antibiotics-15-00789-s001.zip › 2_Decalin Polyketides_SI.pdf]

Supplementary Information

Tandyukisins K−P, New Decalin Polyketides from *Trichoderma longicollum* 17007 with Antitubercular Activity against *Mycobacterium tuberculosis* H37Ra

Junjie Yu [1], Shangqian Ning [1], Yuchang Di [2], Hongbo Feng [1], Xiaoying Li [1], Kun Wang [1], Rongxuan Wang [1], Menghan Ma [1], Sicheng Pan [1], Chenglin Jiang [3], Yi Jiang [3], Tom Hsiang [4], Jiazhen Chen [2], Wenhong Zhang [2], Lixin Zhang [1], Xueting Liu [1], Xuelian Zhang [2,*] and Guoliang Zhu [1,*]

[1]State Key Laboratory of Bioreactor Engineering, East China University of Science and Technology, Shanghai 200237, China

[2]State Key Laboratory of Genetic Engineering, School of Life Sciences, Department of Infectious Diseases, Shanghai Key Laboratory of Infectious Diseases and Biosafety Emergency Response, National Medical Center for Infectious Diseases, Huashan Hospital, Fudan University, Shanghai, China

[3]The Key Lab for Research & Development of Actinomycete Resources, Yunnan Institute of Microbiology, Yunnan University, Chenggong District, Kunming 650500, China.

[4]School of Environmental Sciences, University of Guelph, Guelph, Ontario N1G 2W1, Canada

*Corresponding authors: Guoliang Zhu, Xuelian Zhang

E-mail: zhuguoliang@ecust.edu.cn, xuelianzhang@fudan.edu.cn

Phone (Fax): CN +86-021-64253020.

Tables of Contents

Supplementary Methods **5**

Trichoderma longicollum **17007 ITS sequence**.. 5

Supplementary Figures **6**

Figure S1. Identification of strain *Trichoderma* sp. 17007. 6

Figure S2. LC-DAD-HRMS analysis of unidentified metabolites in the decalin fraction E2G1–3... 7

Figure S3. The structure of known tandyukisin-type decalin polyketides.. 8

Figure S4a. HR-ESI (Pos)-MS spectrum of tandyukisin K (**1**) 9

Figure S4b. ^{1}H NMR (600 MHz, Methanol-d_4) spectrum of tandyukisin K (**1**) 9

Figure S4c. ^{13}C NMR (150 MHz, Methanol-d_4) spectrum of tandyukisin K (**1**).. 10

Figure S4d. HSQC spectrum of tandyukisin K (**1**).. 10

Figure S4e. ^{1}H-^{1}H COSY spectrum of tandyukisin K (**1**).. 11

Figure S4f. HMBC spectrum of tandyukisin K (**1**).. 11

Figure S4g. NOESY spectrum of tandyukisin K (**1**).. 12

Figure S5a. HR-ESI (Pos)-MS spectrum of tandyukisin L (**2**).. 13

Figure S5b. ^{1}H NMR (600 MHz, Methanol-d_4) spectrum of tandyukisin L (**2**).. 13

Figure S5c. ^{13}C NMR (150 MHz, Methanol-d_4) spectrum of tandyukisin L (**2**) 14

Figure S5d. HSQC spectrum of tandyukisin L (**2**).. 14

Figure S5e. ^{1}H-^{1}H COSY spectrum of tandyukisin L (**2**) 15

Figure S5f. HMBC spectrum of tandyukisin L (**2**) 15

Figure S5g. NOESY spectrum of tandyukisin L (**2**) 16

Figure S6a. HR-ESI (Pos)-MS spectrum of tandyukisin M (**3**).. 17

Figure S6b. ^{1}H NMR (600 MHz, Methanol-d_4) spectrum of tandyukisin M (**3**).. 17

Figure S6c. ^{13}C NMR (150 MHz, Methanol-d_4) spectrum of tandyukisin M (**3**) 18

Figure S6d. HSQC spectrum of tandyukisin M (**3**) 18

Figure S6e. ^{1}H-^{1}H COSY spectrum of tandyukisin M (**3**) 19

Figure S6f. HMBC spectrum of tandyukisin M (**3**) 19

Figure S6g. NOESY spectrum of tandyukisin M (**3**)......20
Figure S7a. HR-ESI (Pos)-MS spectrum of tandyukisin N (**4**)......21
Figure S7b. ^{1}H NMR (600 MHz, Methanol-d_4) spectrum of tandyukisin N (**4**)......21
Figure S7c. ^{13}C NMR (150 MHz, Methanol-d_4) spectrum of tandyukisin N (**4**)......22
Figure S7d. HSQC spectrum of tandyukisin N (**4**)......22
Figure S7e. ^{1}H-^{1}H COSY spectrum of tandyukisin N (**4**)......23
Figure S7f. HMBC spectrum of tandyukisin N (**4**)......23
Figure S7g. NOESY spectrum of tandyukisin N (**4**)......24
Figure S8a. HR-ESI (Pos)-MS spectrum of tandyukisin O (**5**)......25
Figure S8b. ^{1}H NMR (600 MHz, Methanol-d_4) spectrum of tandyukisin O (**5**)......25
Figure S8c. ^{13}C NMR (150 MHz, Methanol-d_4) spectrum of tandyukisin O (**5**)......26
Figure S8d. HSQC spectrum of tandyukisin O (**5**)......26
Figure S8e. ^{1}H-^{1}H COSY spectrum of tandyukisin O (**5**)......27
Figure S8f. HMBC spectrum of tandyukisin O (**5**)......27
Figure S8g. NOESY spectrum of tandyukisin O (**5**)......28
Figure S9a. HR-ESI (Pos)-MS spectrum of tandyukisin P (**6**)......29
Figure S9b. ^{1}H NMR (600 MHz, Methanol-d_4) spectrum of tandyukisin P (**6**)......29
Figure S9c. ^{13}C NMR (150 MHz, Methanol-d_4) spectrum of tandyukisin P (**6**)......30
Figure S9d. HSQC spectrum of tandyukisin P (**6**)......30
Figure S9e. ^{1}H-^{1}H COSY spectrum of tandyukisin P (**6**)......31
Figure S9f. HMBC spectrum of tandyukisin P (**6**)......31
Figure S9g. NOESY spectrum of tandyukisin P (**6**)......32
Figure S10a. HR-ESI (Pos)-MS spectrum of trichoharzin (**7**)......33
Figure S10b. ^{1}H NMR (600 MHz, $CDCl_3$) spectrum of trichoharzin (**7**)......33
Figure S10c. ^{13}C NMR (150 MHz, $CDCl_3$) spectrum of trichoharzin (**7**)......34
Figure S11a. HR-ESI (Pos)-MS spectrum of tandyukisin J (**8**)......35
Figure S11b. ^{1}H NMR (600 MHz, $CDCl_3$) spectrum of tandyukisin J (**8**)......35
Figure S11c. ^{13}C NMR (150 MHz, $CDCl_3$) spectrum of tandyukisin J (**8**)......36

Figure S12. Putative origins of the 3-methylpentenedioate-type acyl unit. 36

Supplementary Tables **37**

Table S1. Annotations of the 23 nodes in the decalin cluster I from the *T. longicollum* 17007 featured-based molecular networking 37

Table S2. Comparative ^{13}C NMR features and side-chain assignments of reported tandyukisin-type decalin polyketides 38

Table S3. ^{1}H and ^{13}C NMR Data of trichoharzin (**7**) and tandyukisin J (**8**) in $CDCl_3$ 39

Table S4. *In vitro* bioactivity evaluation of compounds **1**–**8** 40

Table S5. Deduced functions of ORFs in tandyukisins (*tan*) BGC from *T. longicollum* 17007 based on top BLASTP matches 41

Supplementary Methods

***Trichoderma longicollum* 17007 ITS sequence**

TTCCTTACCATGATCCGAGGTCACATTTCAGAAGTTGGGTGTTTAACGGCTGTGGACGCGCCGCGCTCCCGATGCGAGTGTGCAAACTACTGCGCAGGAGAGGCTGCGGCGAGACCGCCACTGTATTTCGGAGACGGCCACCGCCAAGAGGCAGGGCCGATCCCCAACGCCGACCCCCCGGAGGGGTTCGAGGGTTGAAATGACGCTCGGACAGGCATGCCCGCCAGAATACTGGCGGGCGCAATGTGCGTTCAAAGATTCGATGATTCACTGAATTCTGCAATTCACATTACTTATCGCATTTCGCTGCGTTCTTCATCGATGCCAGAACCAAGAGATCCGTTGTTGAAAGTTTTGATTCATTTTCGAAACGCCTACGAGAGGCGCCGAGAAGGCTCAGATTATAAAAAAAACCCGCGAGGGGGTATACAAAAAGAGTTTTGGTTGGTCCTCCGGCGGGCGCCTTGGTCCGGGGCTGCGACGCACCCGGGGCAGAGATCCCGCCGAGGCAACAGTTTGGTAACGTTCACATTGGGTTTGGGAGTTGTAAACTCGGTAATGATCCCTCCGCTGGTTCACCAACGGAGACCTTGTTACGATTTTTACTTCCA

Supplementary Figures

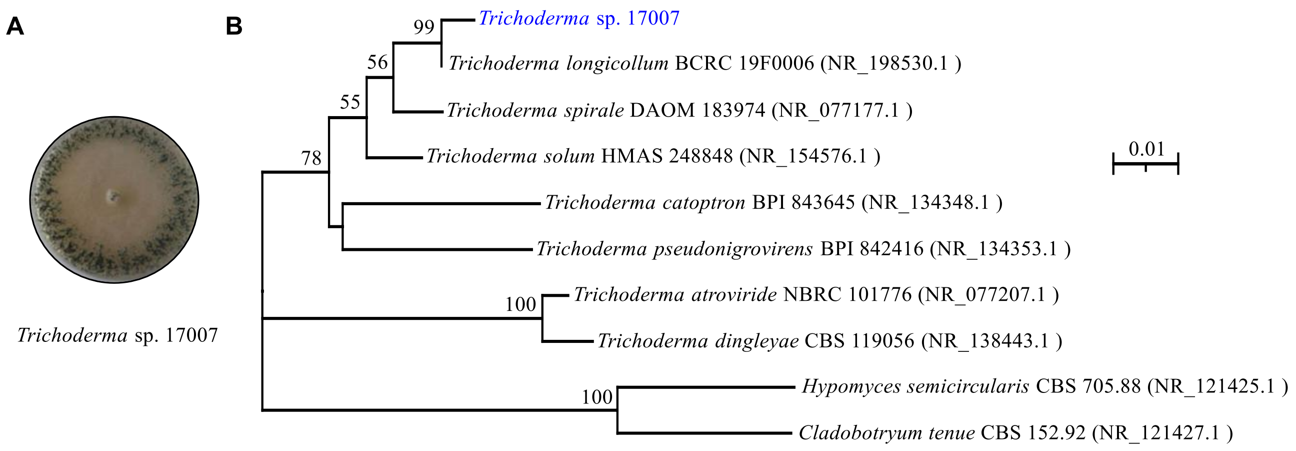

Figure S1. Identification of strain *Trichoderma* sp. 17007. (**A**) Colony morphology of strain *Trichoderma* sp. 17007 cultured at 28°C on a PDA medium plate for 10 days. (**B**) Neighbor-joining phylogenetic tree based on ITS sequences. Bootstrap values were calculated from 1000 replicates, and only values greater than 50% are shown. NCBI accession numbers are provided in parentheses. The scale bar represents 0.01 substitutions per site.

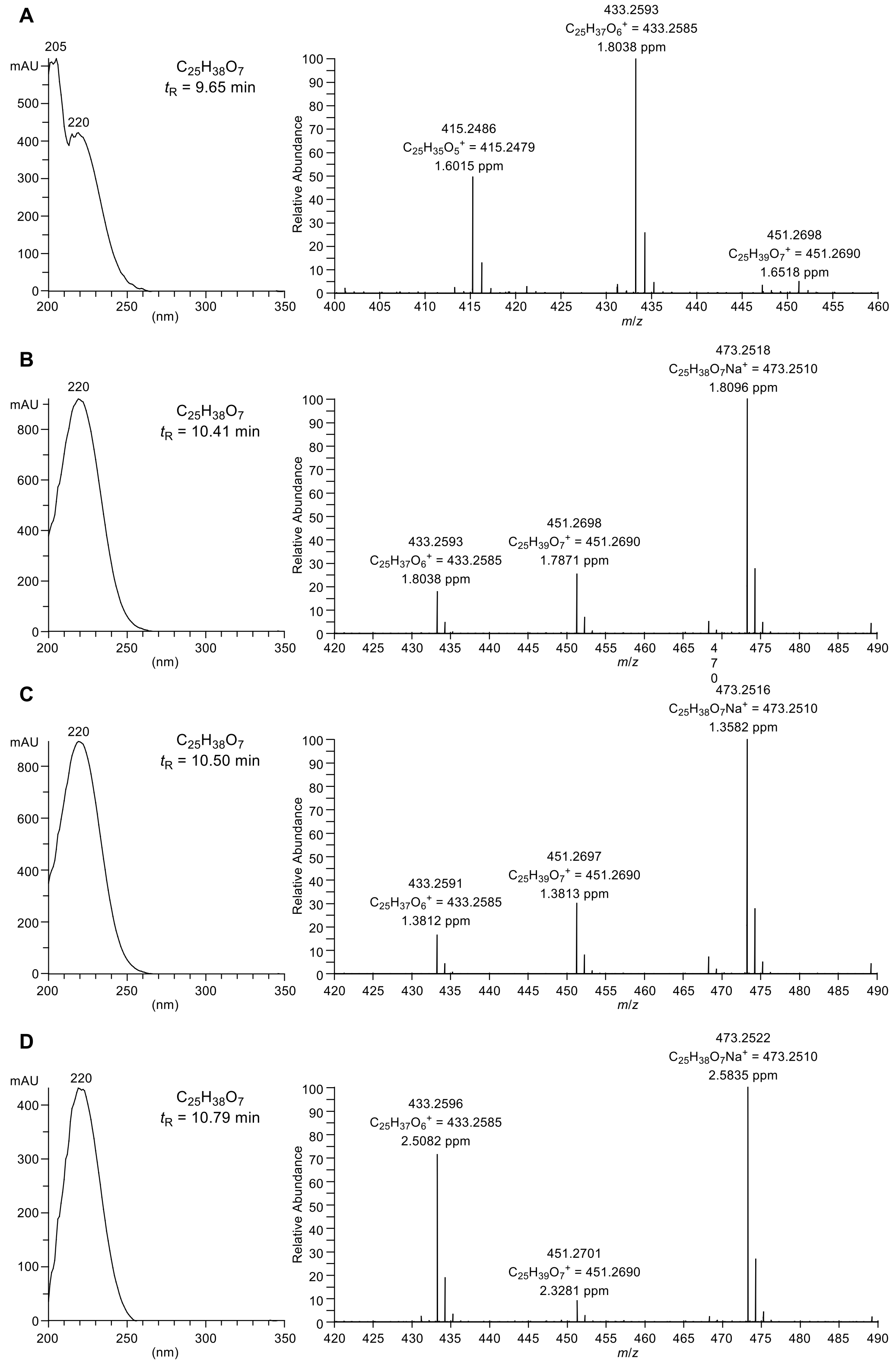

Figure S2. LC-DAD-HRMS analysis of unidentified metabolites in the decalin fraction E2G$_{1–3}$.

A–D. UV absorption and HR-ESI-MS spectra of the metabolites eluting at t_R = 9.65, 10.41, 10.50, and 10.79 min, respectively.

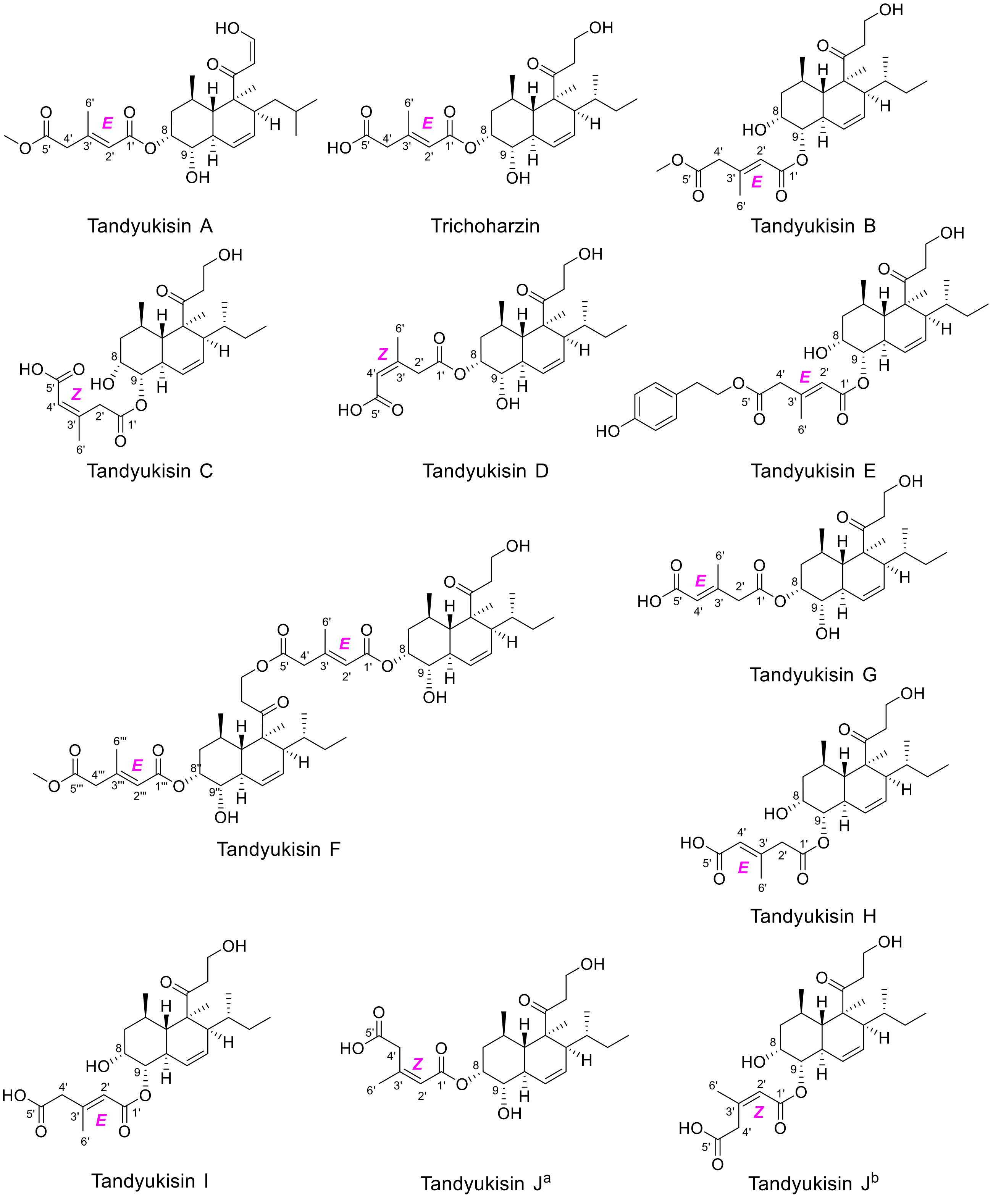

Figure S3. The structure of known tandyukisin-type decalin polyketides. ([a]reported on *Mar. Drugs* 2023, *21*(11), 566; [b]reported on *Nat. Prod. Res* 2026, *40*(14), 4256-4263.)

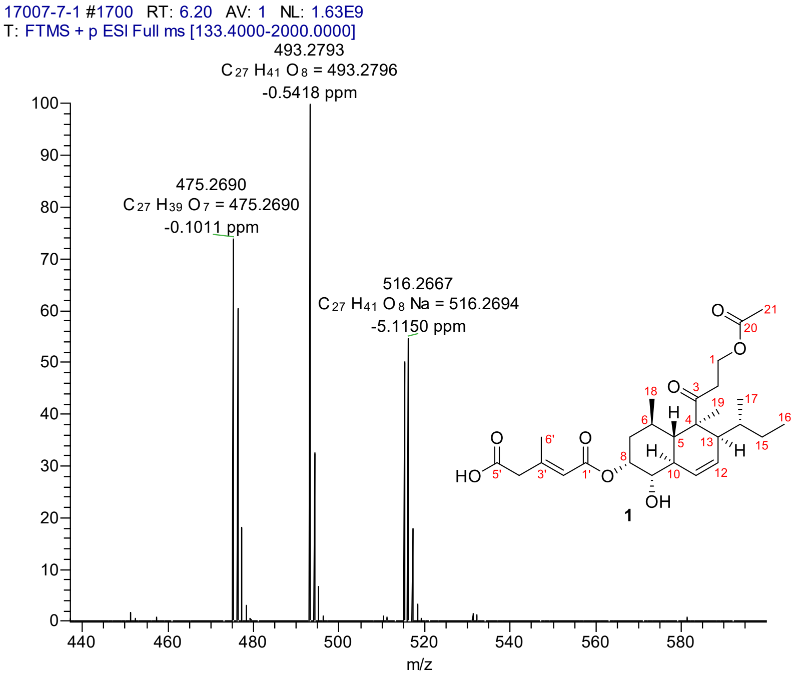

Figure S4a. HR-ESI (Pos)-MS spectrum of tandyukisin K (**1**)

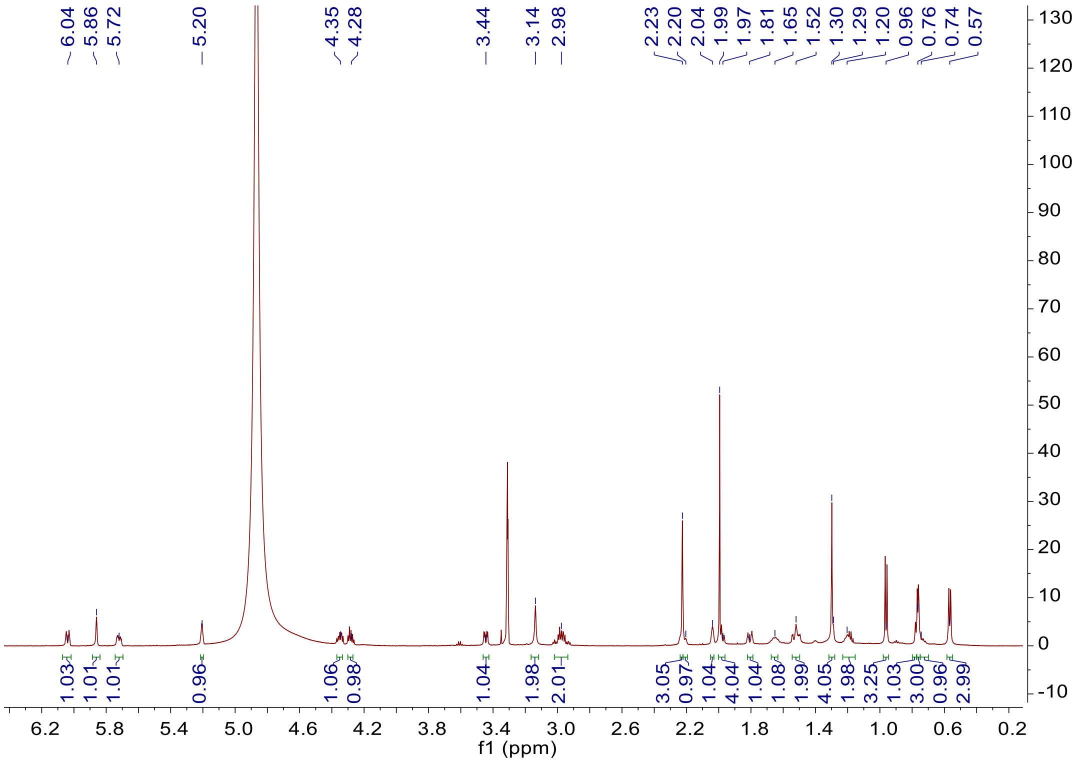

Figure S4b. ^{1}H NMR (600 MHz, Methanol-*d*4) spectrum of tandyukisin K (**1**)

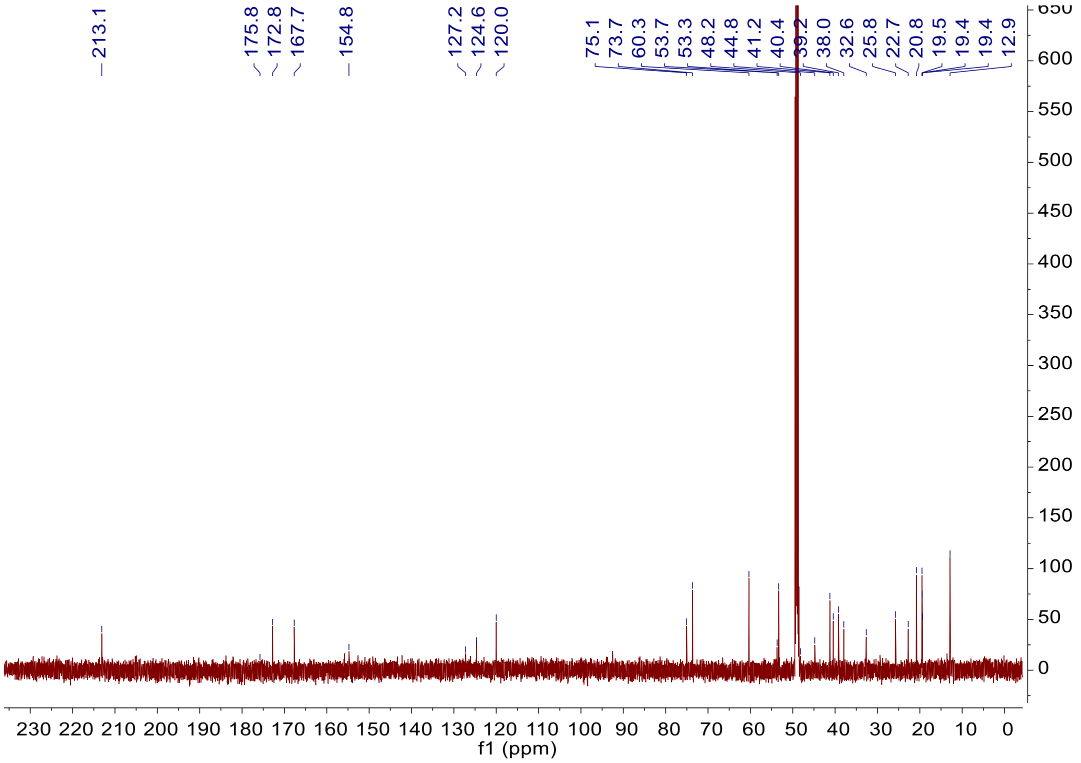

Figure S4c. ^{13}C NMR (150 MHz, Methanol-d_4) spectrum of tandyukisin K (**1**)

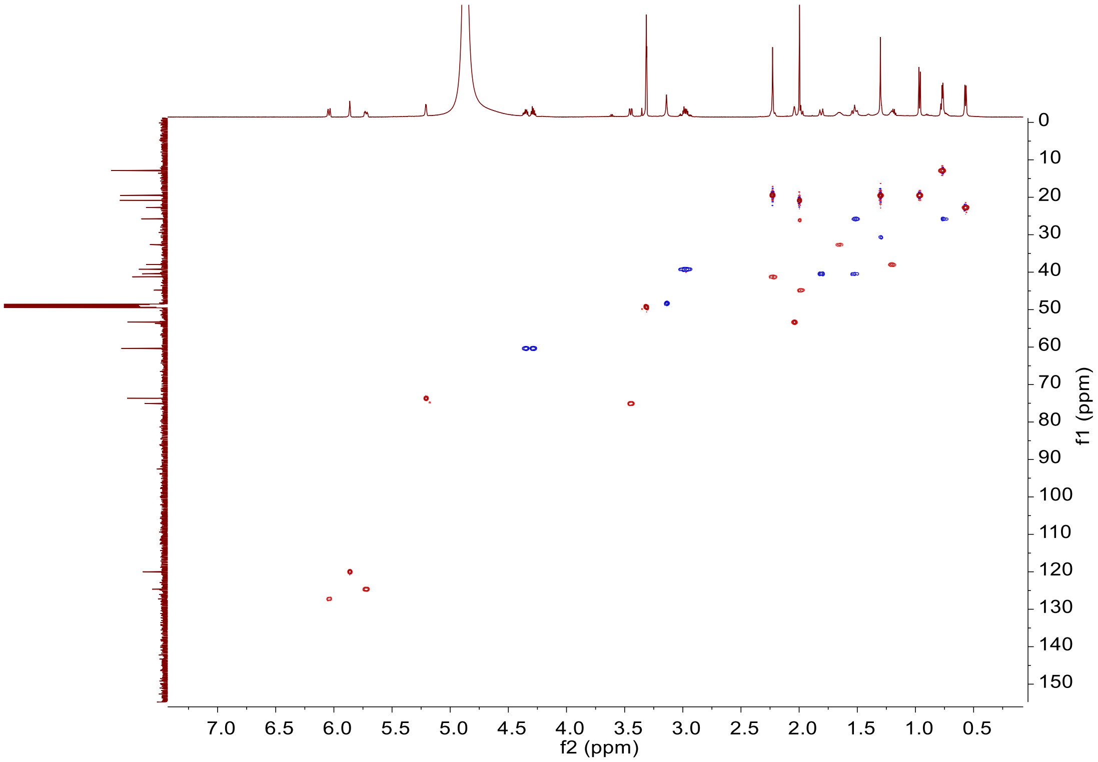

Figure S4d. HSQC spectrum of tandyukisin K (**1**)

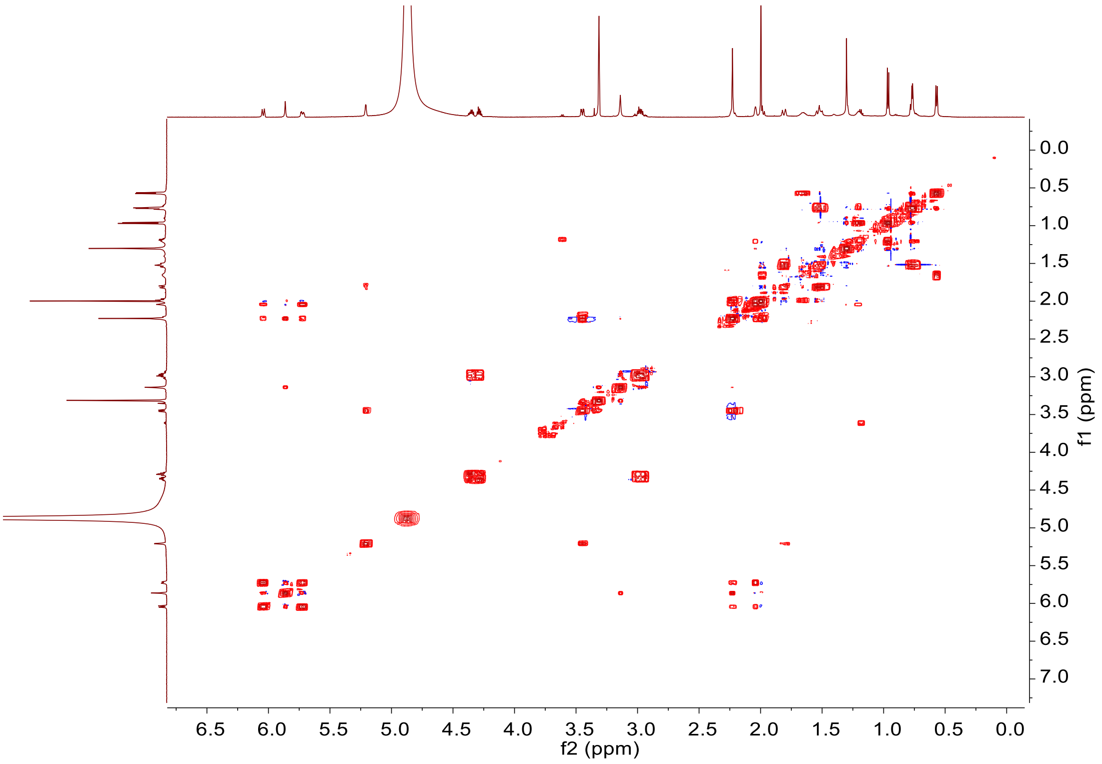

Figure S4e. ^{1}H-^{1}H COSY spectrum of tandyukisin K (**1**)

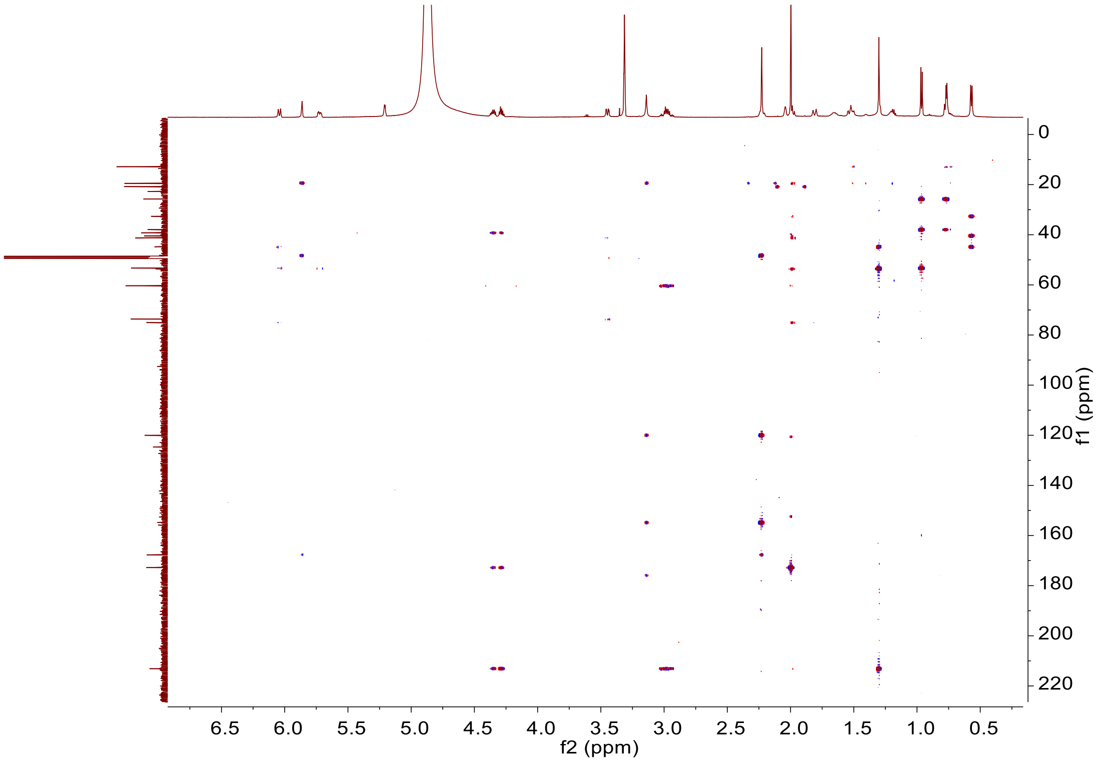

Figure S4f. HMBC spectrum of tandyukisin K (**1**)

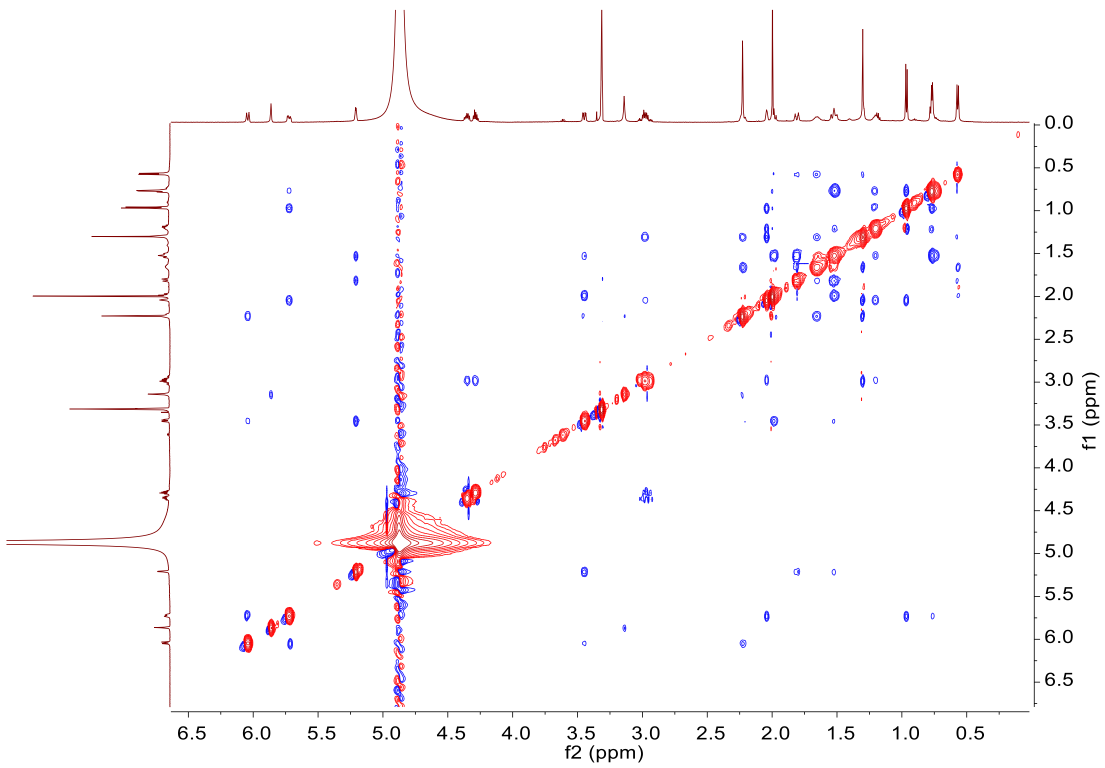

Figure S4g. NOESY spectrum of tandyukisin K (**1**)

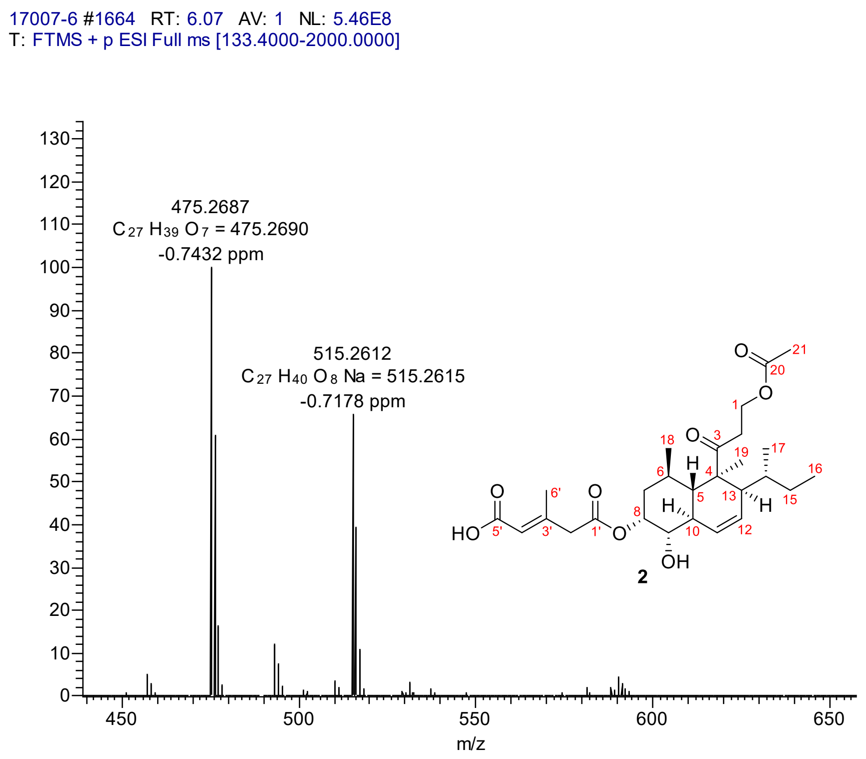

Figure S5a. HR-ESI (Pos)-MS spectrum of tandyukisin L (**2**)

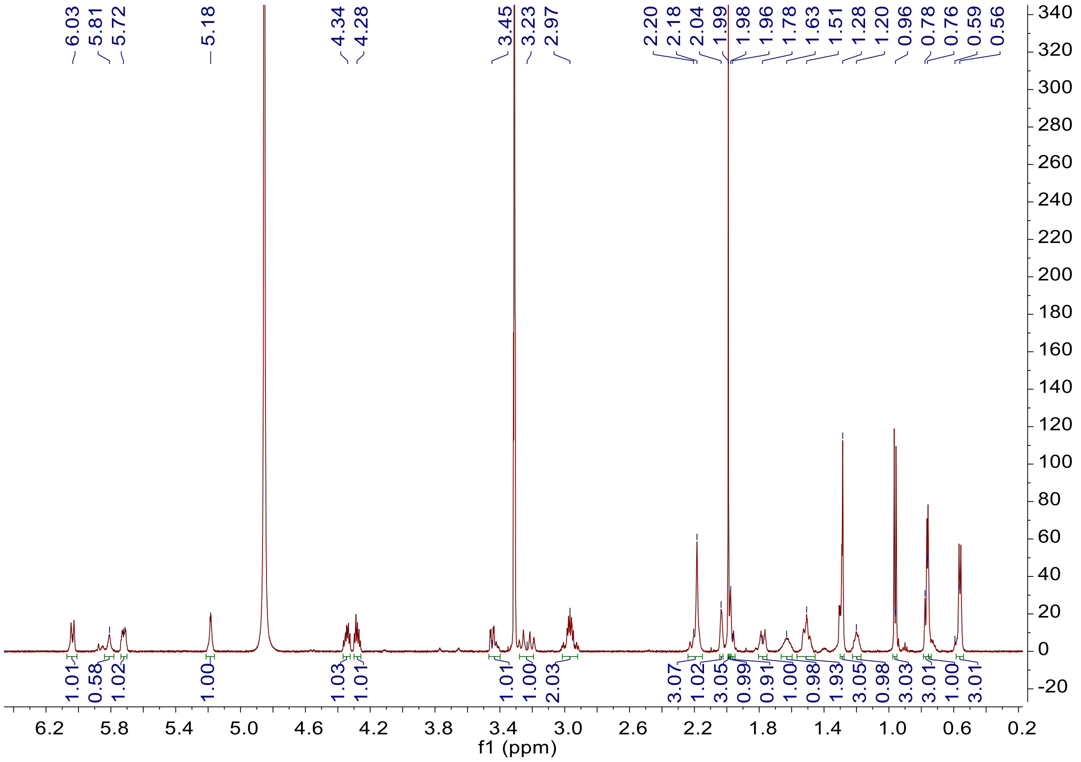

Figure S5b. ^{1}H NMR (600 MHz, Methanol-d_4) spectrum of tandyukisin L (**2**)

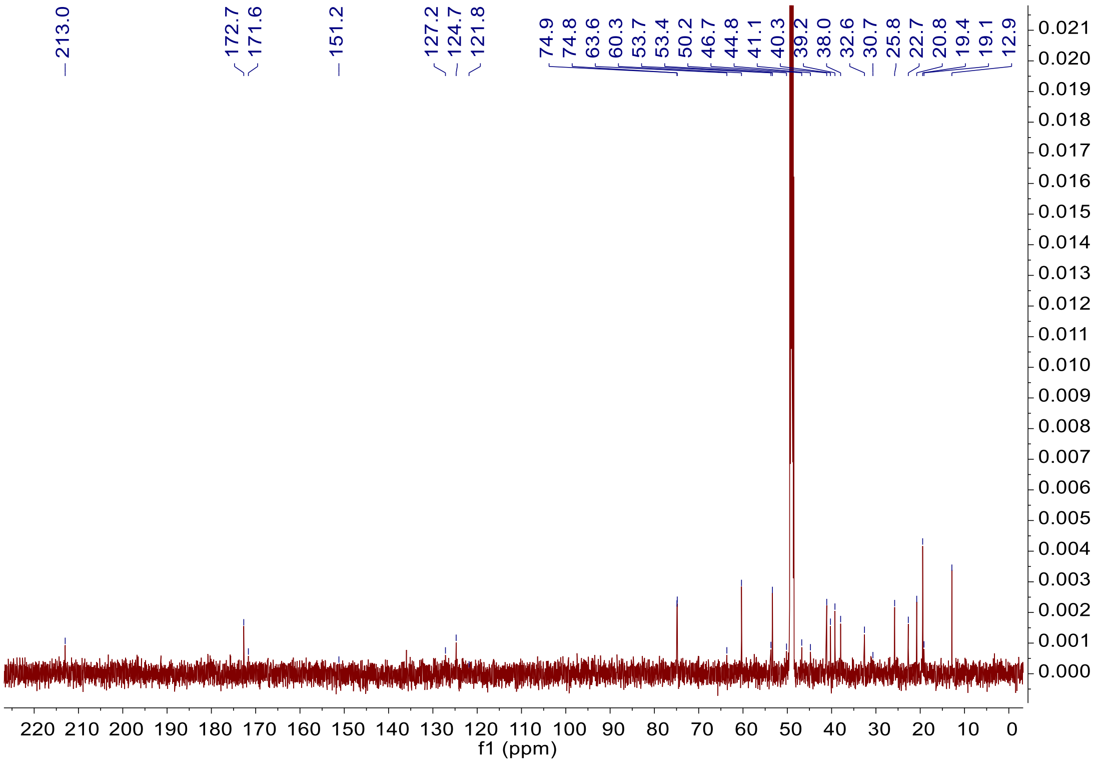

Figure S5c. ^{13}C NMR (150 MHz, Methanol-d_4) spectrum of tandyukisin L (**2**)

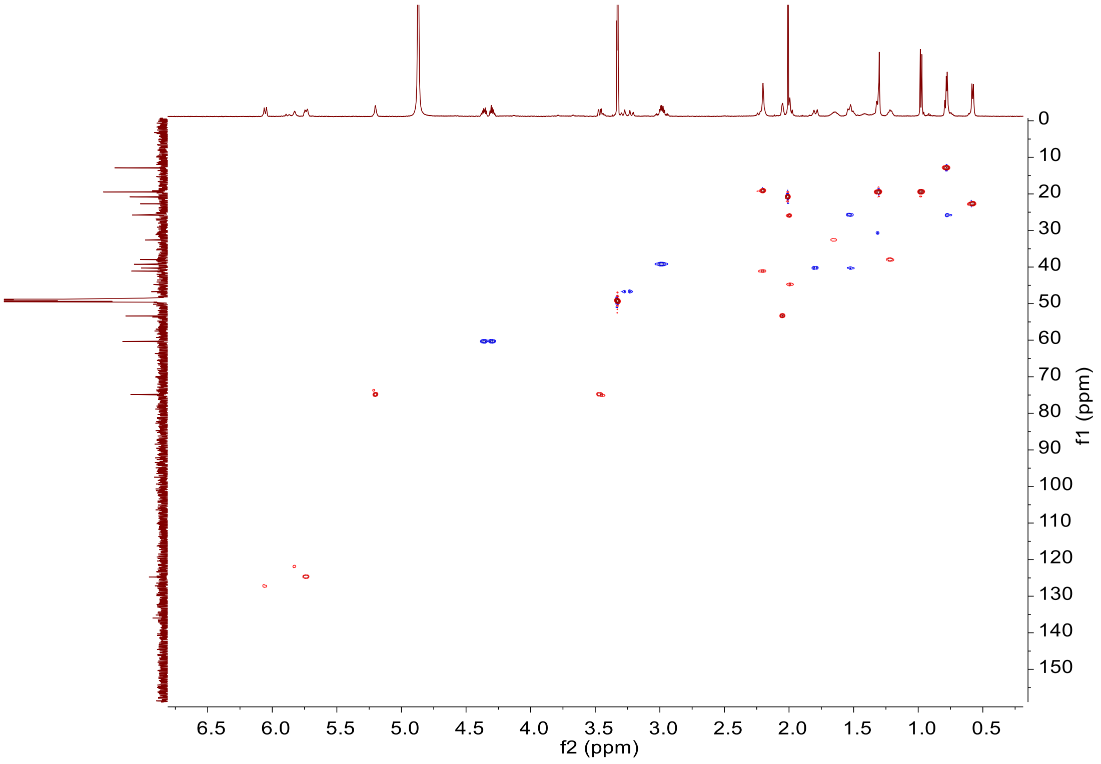

Figure S5d. HSQC spectrum of tandyukisin L (**2**)

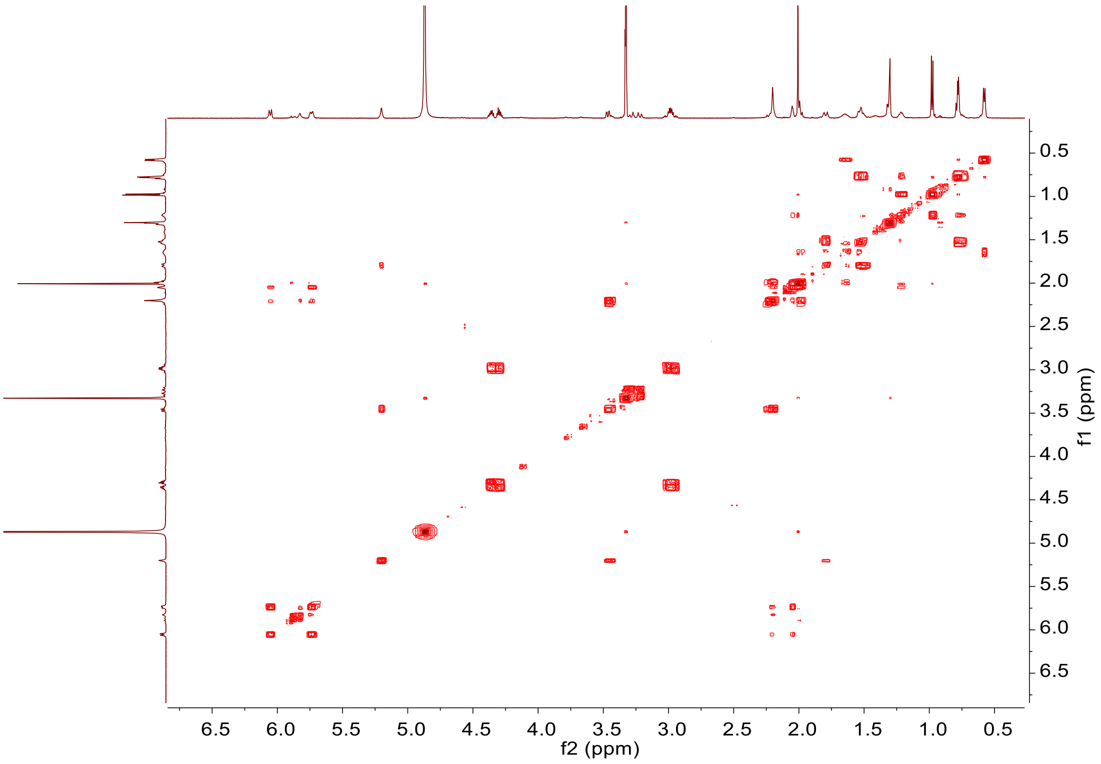

Figure S5e. ^1H-^1H COSY spectrum of tandyukisin L (**2**)

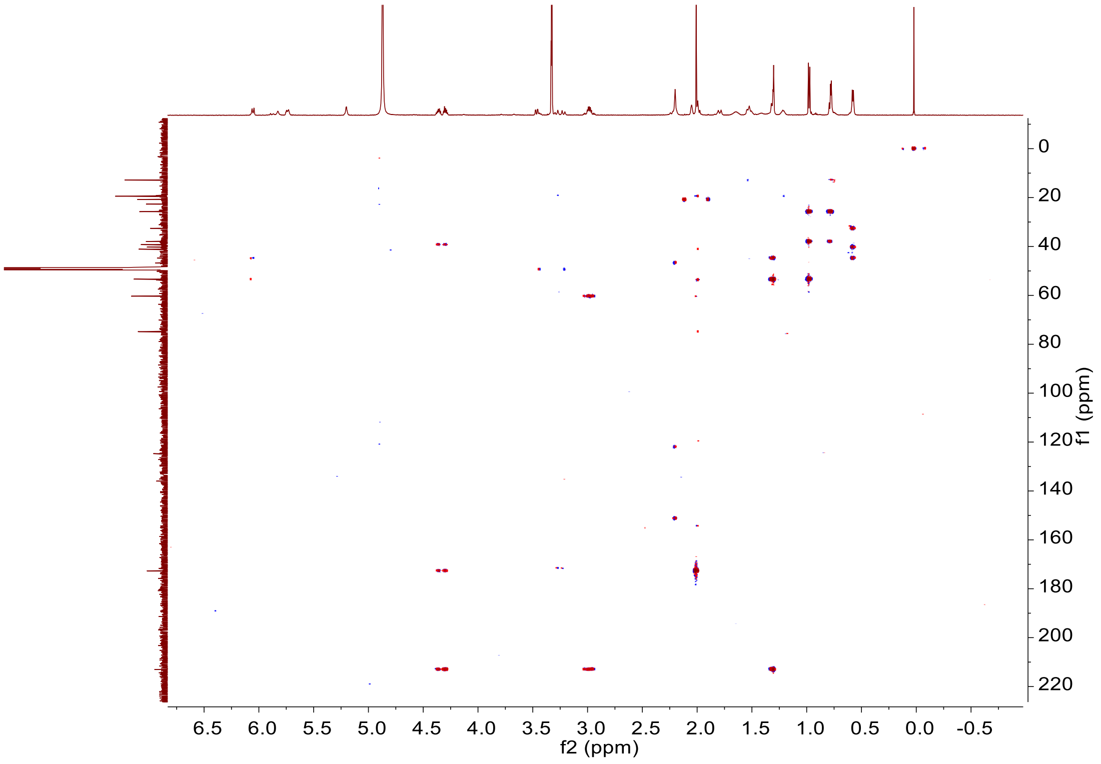

Figure S5f. HMBC spectrum of tandyukisin L (**2**)

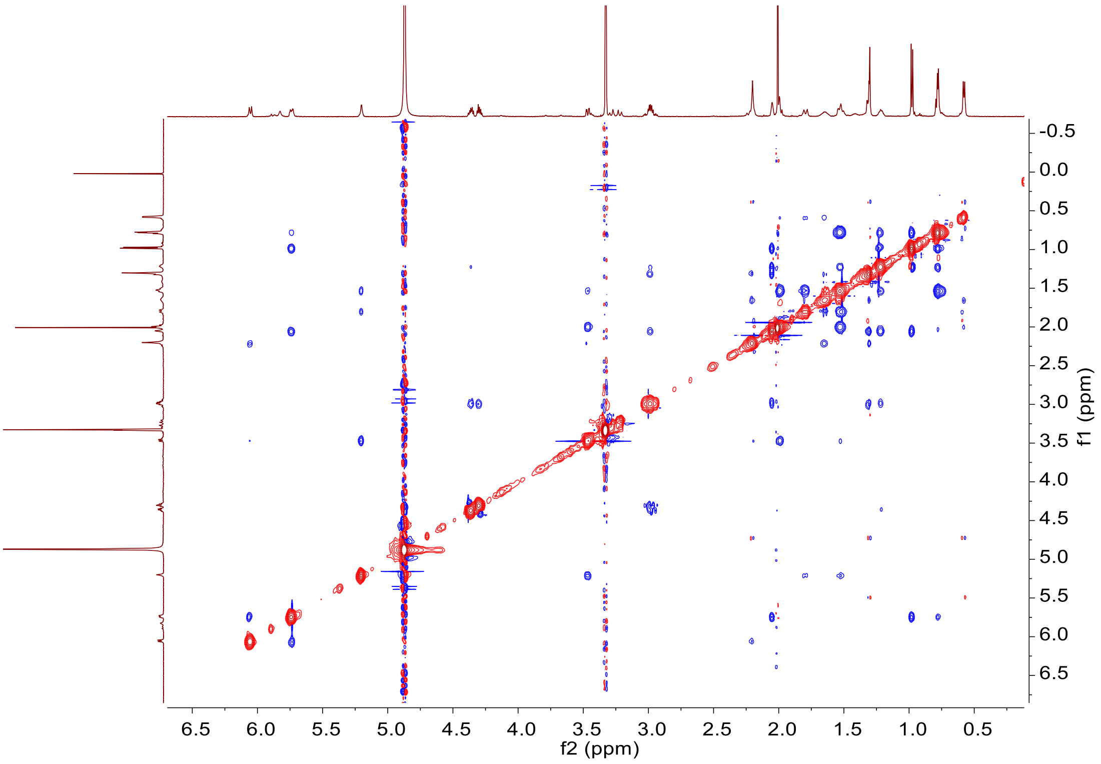

Figure S5g. NOESY spectrum of tandyukisin L (**2**)

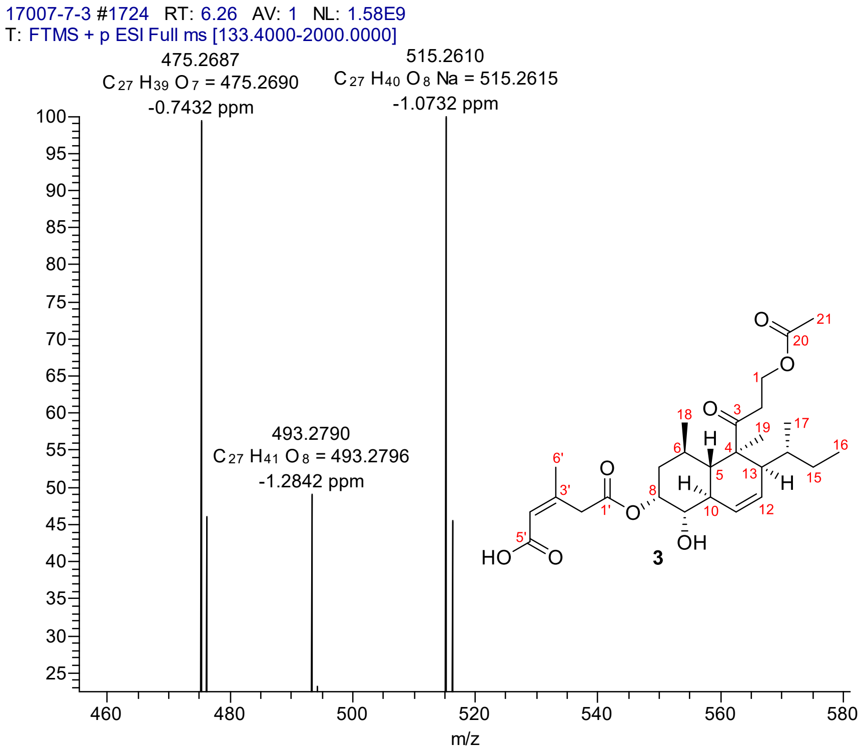

Figure S6a. HR-ESI (Pos)-MS spectrum of tandyukisin M (**3**)

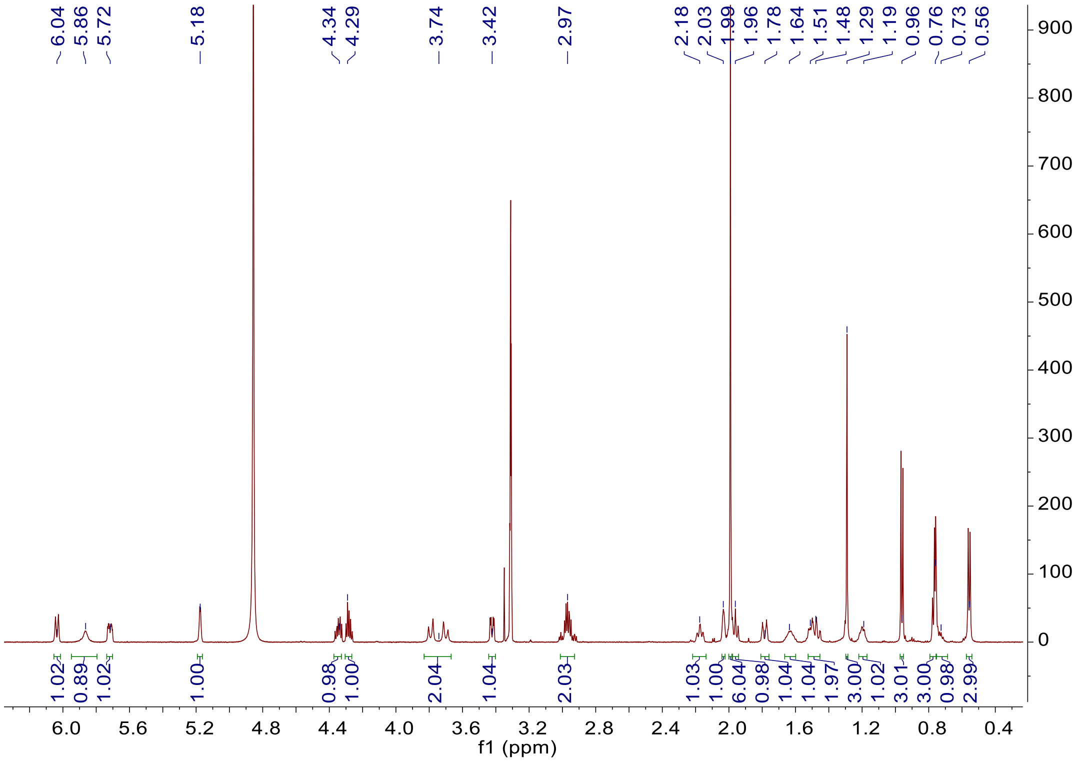

Figure S6b. ^{1}H NMR (600 MHz, Methanol-d_4) spectrum of tandyukisin M (**3**)

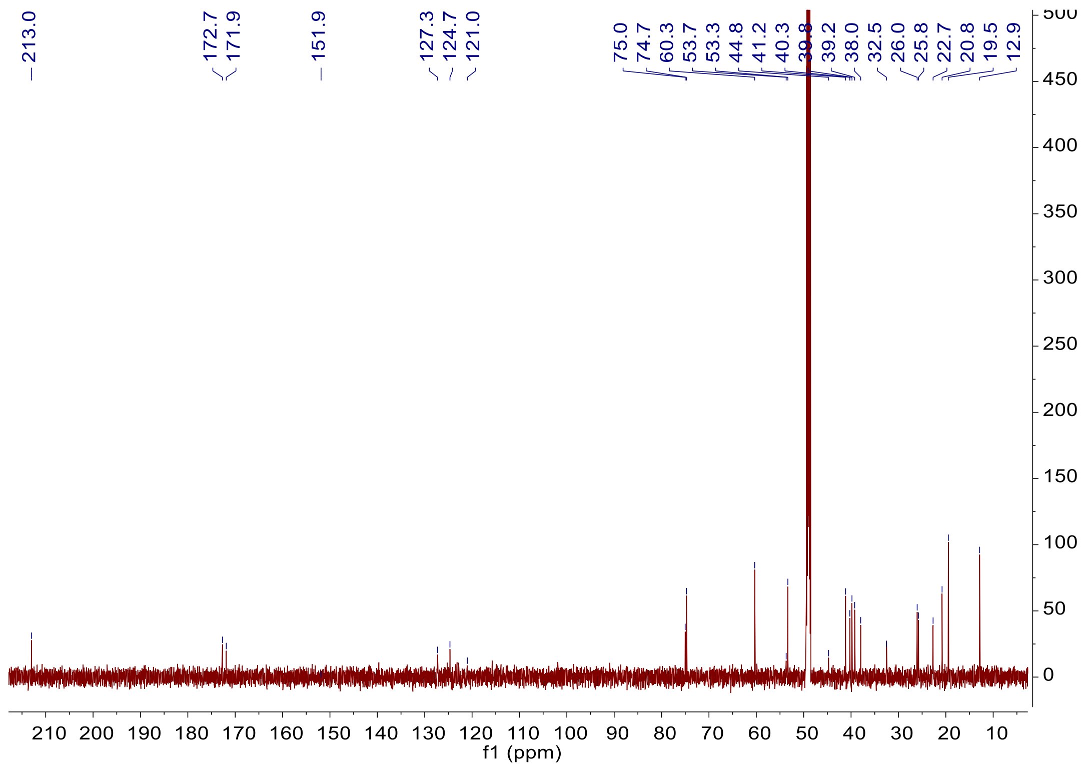

Figure S6c. ^{13}C NMR (150 MHz, Methanol-d_4) spectrum of tandyukisin M (**3**)

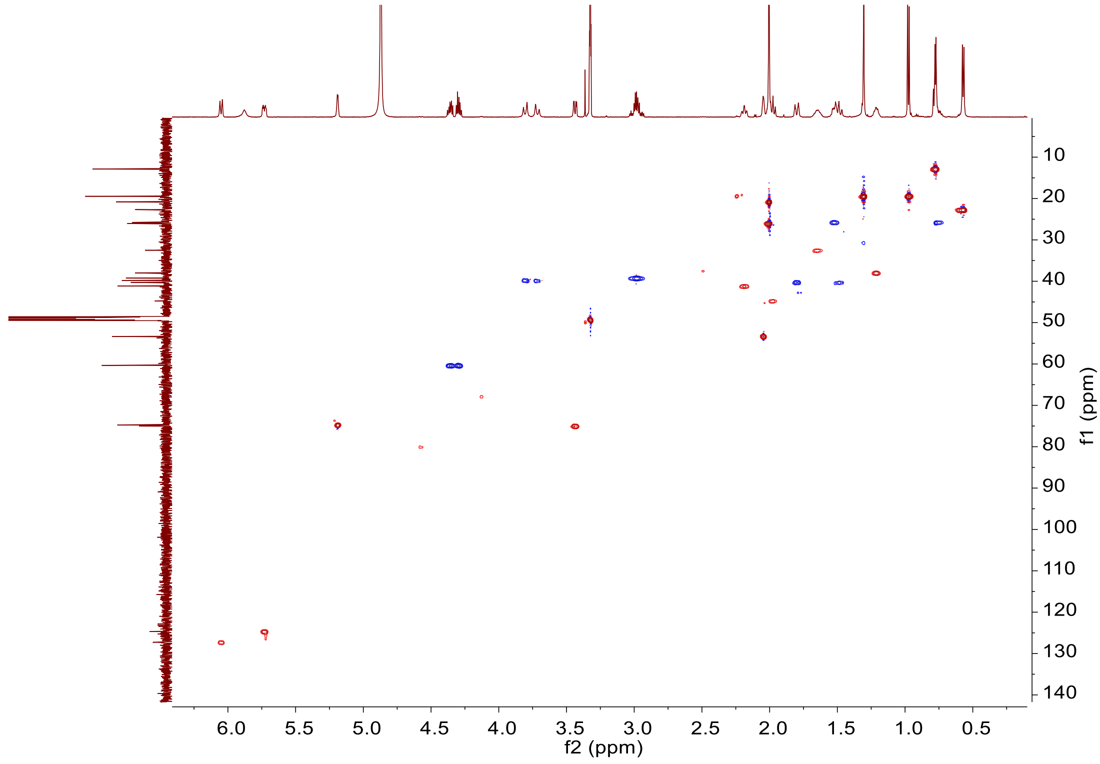

Figure S6d. HSQC spectrum of tandyukisin M (**3**)

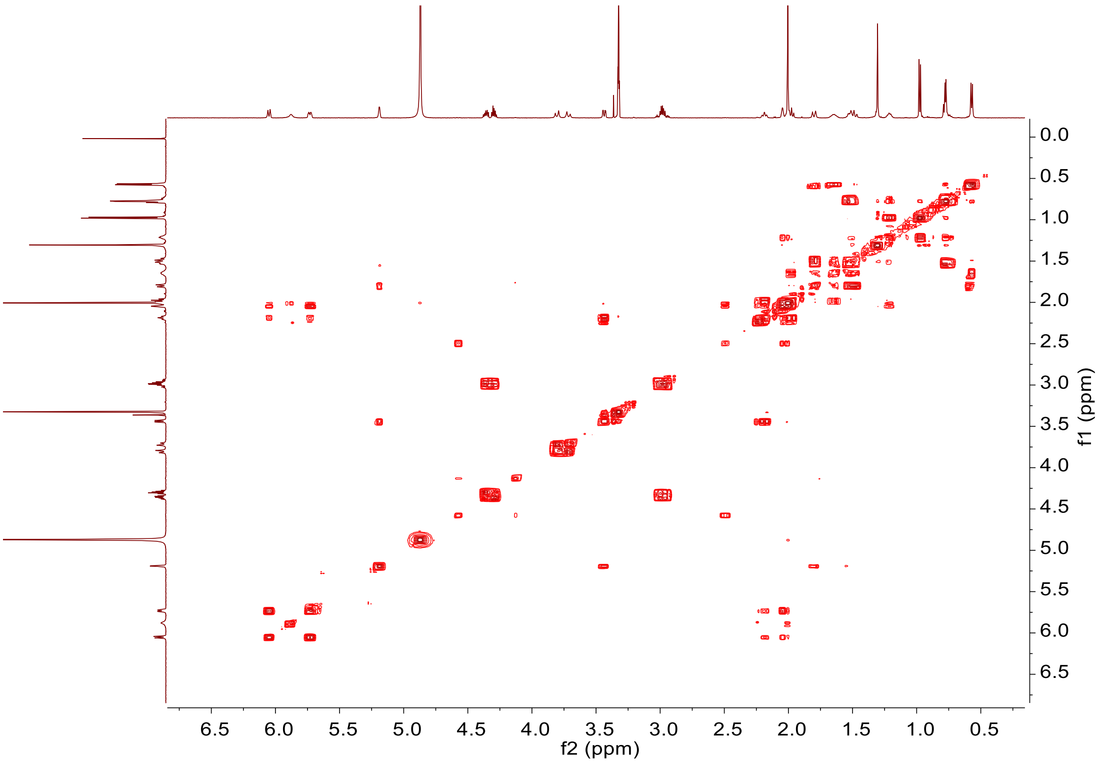

Figure S6e. ^{1}H-^{1}H COSY spectrum of tandyukisin M (**3**)

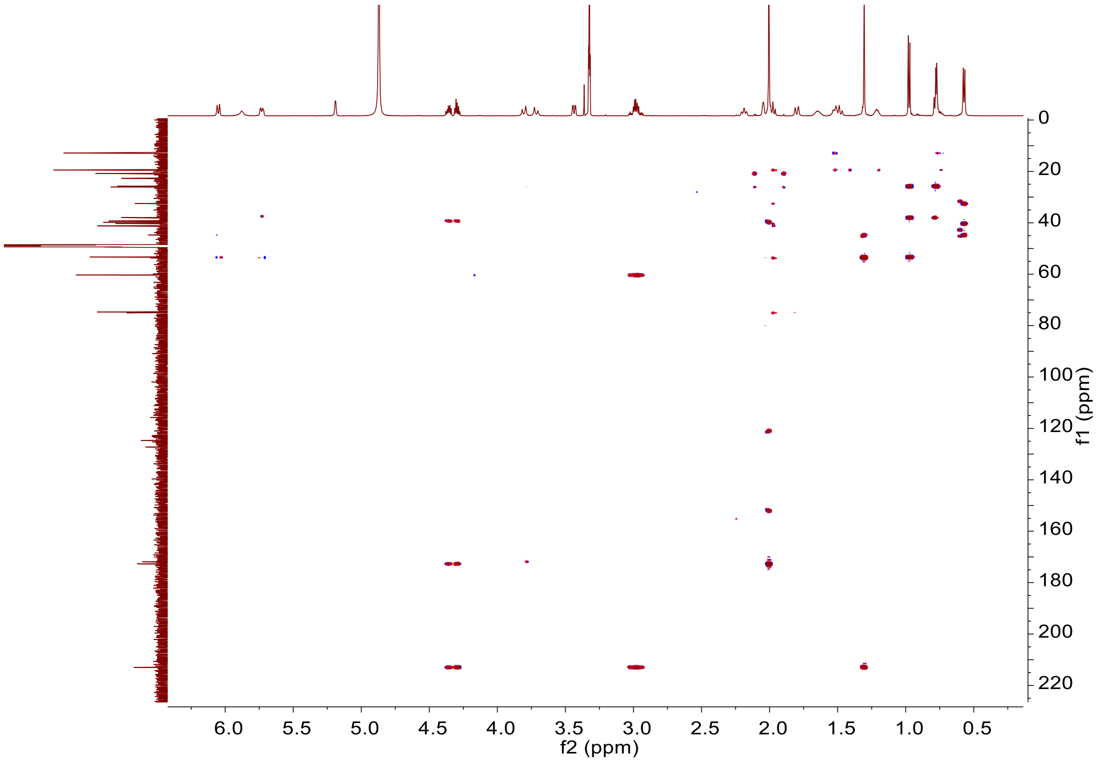

Figure S6f. HMBC spectrum of tandyukisin M (**3**)

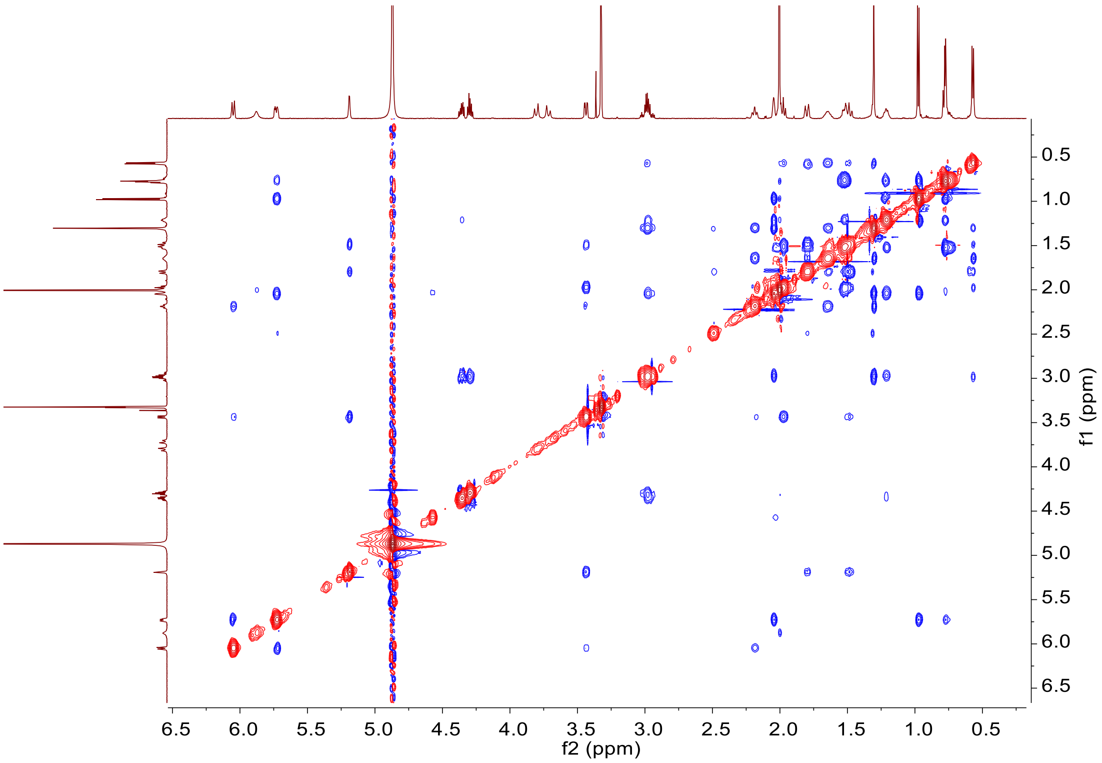

Figure S6g. NOESY spectrum of tandyukisin M (**3**)

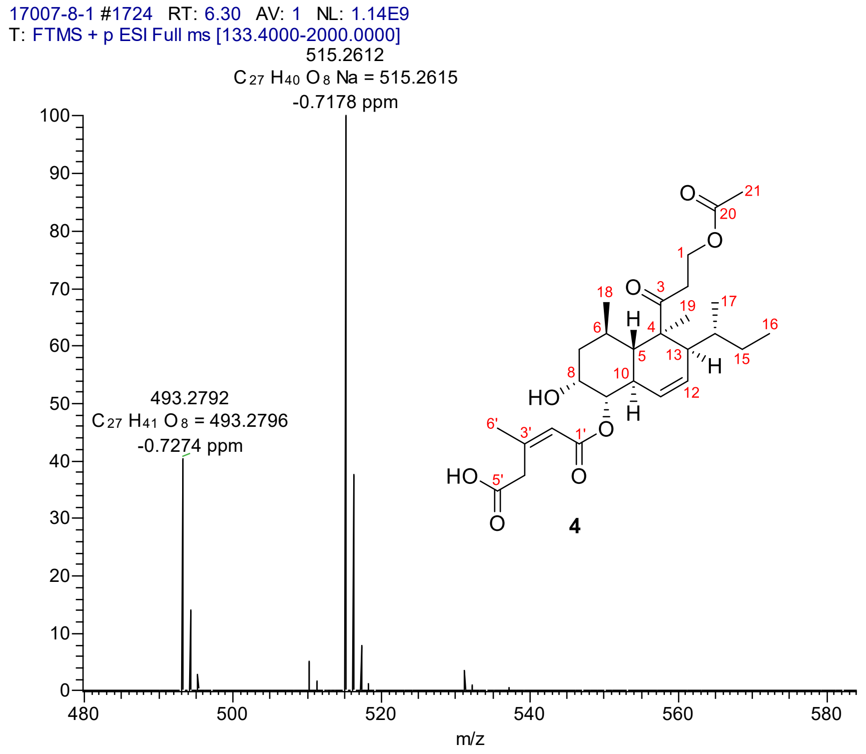

Figure S7a. HR-ESI (Pos)-MS spectrum of tandyukisin N (**4**)

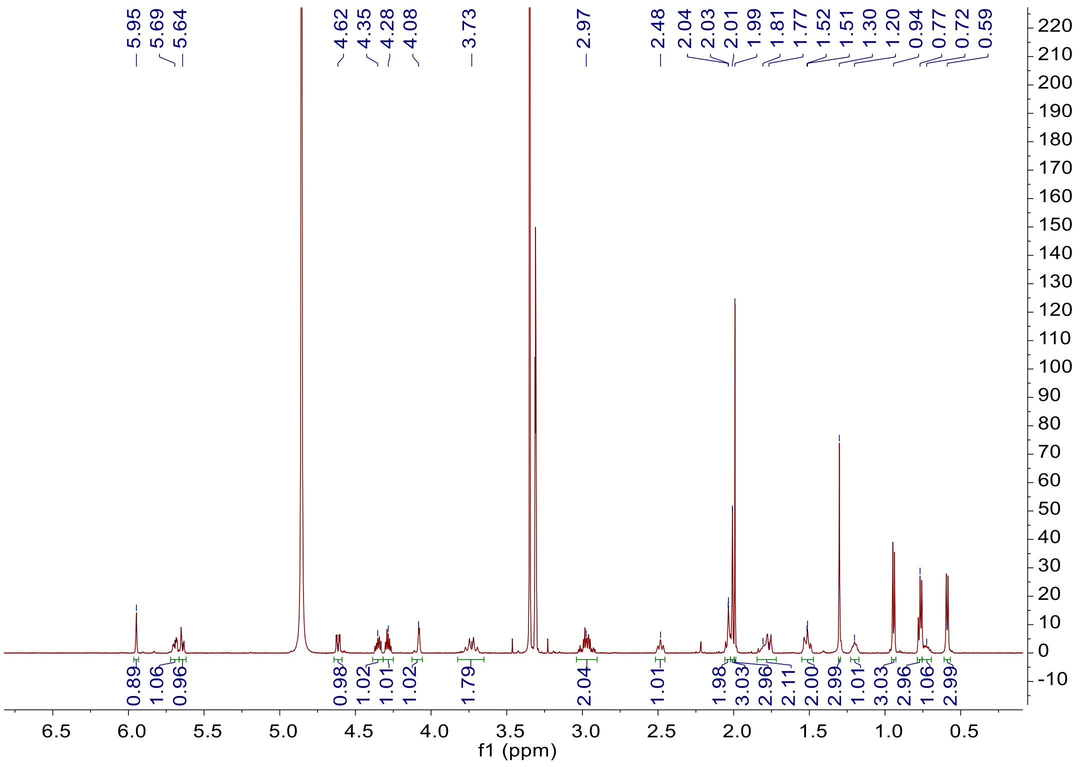

Figure S7b. ^{1}H NMR (600 MHz, Methanol-d_4) spectrum of tandyukisin N (**4**)

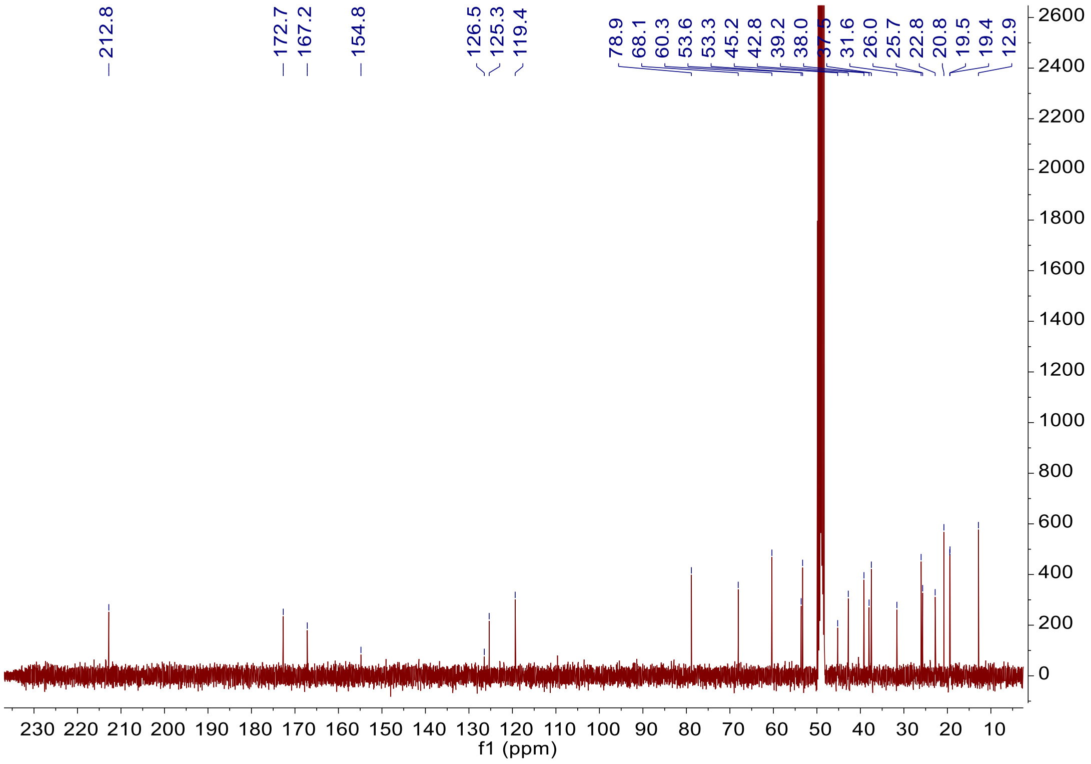

Figure S7c. ^{13}C NMR (150 MHz, Methanol-d_4) spectrum of tandyukisin N (**4**)

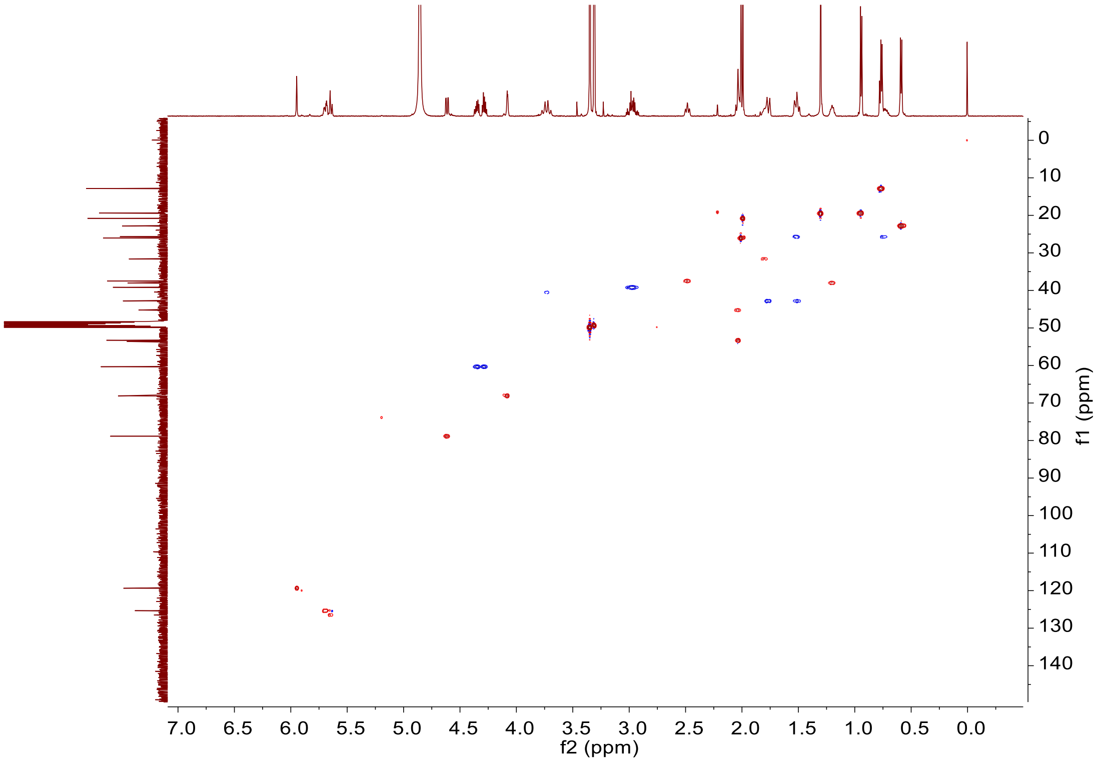

Figure S7d. HSQC spectrum of tandyukisin N (**4**)

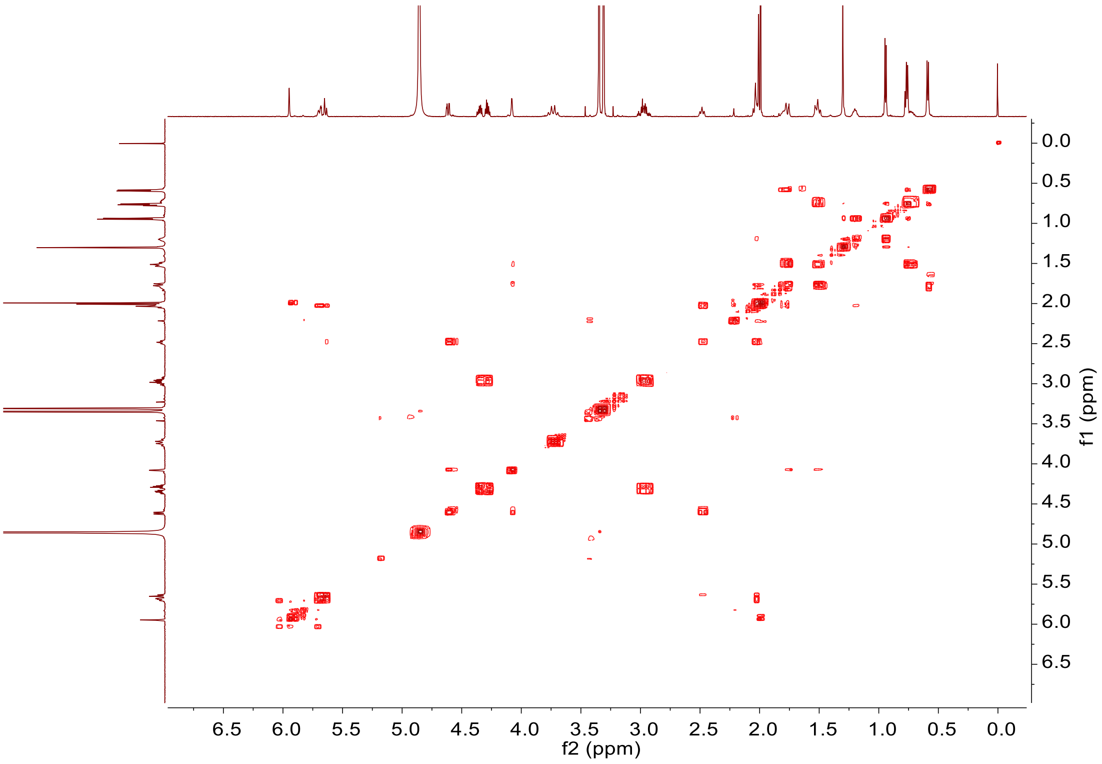

Figure S7e. ^{1}H-^{1}H COSY spectrum of tandyukisin N (**4**)

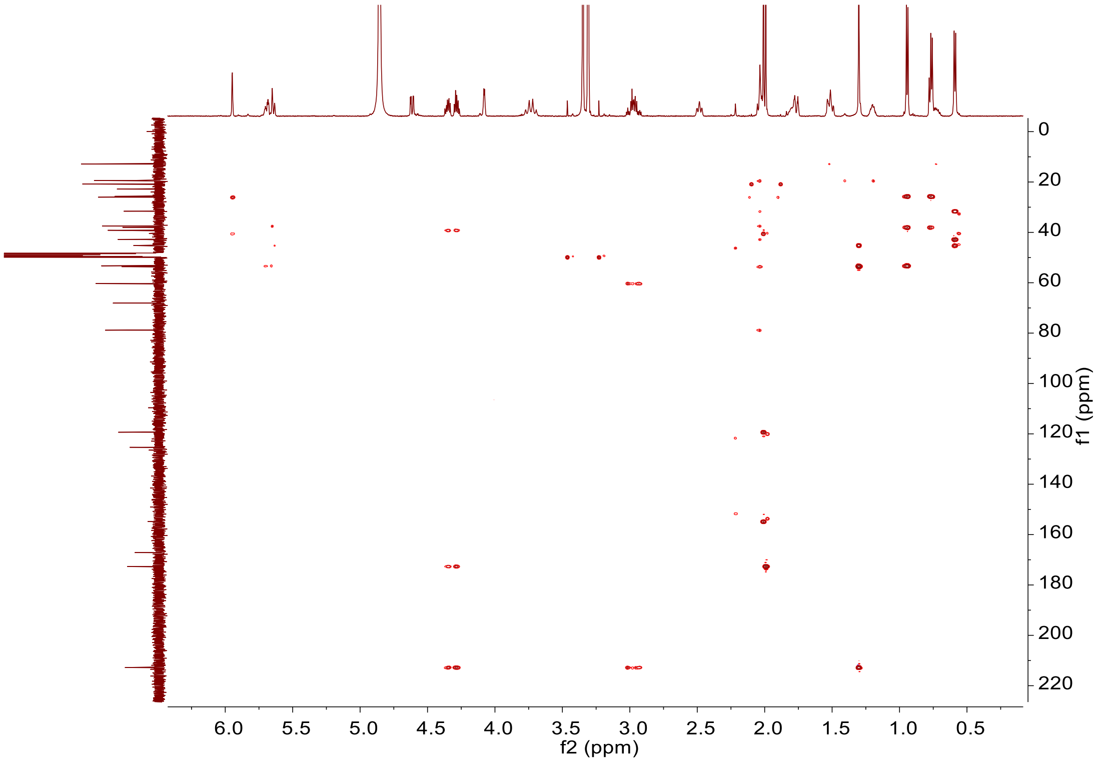

Figure S7f. HMBC spectrum of tandyukisin N (**4**)

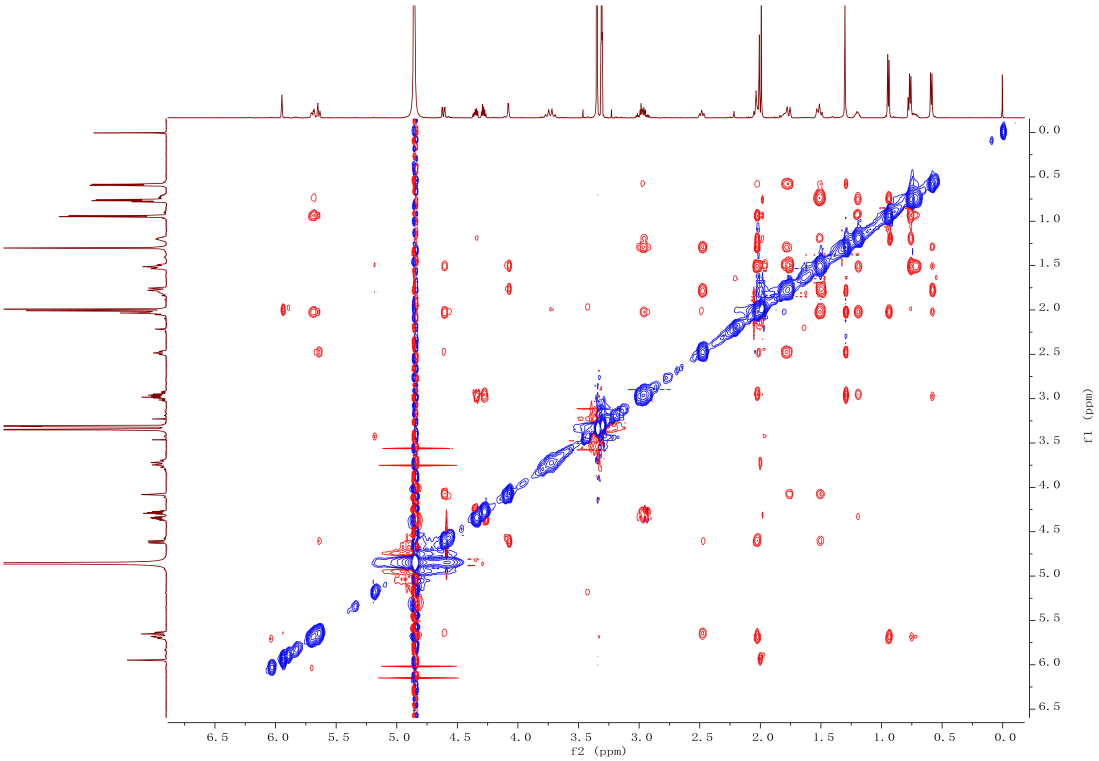

Figure S7g. NOESY spectrum of tandyukisin N (**4**)

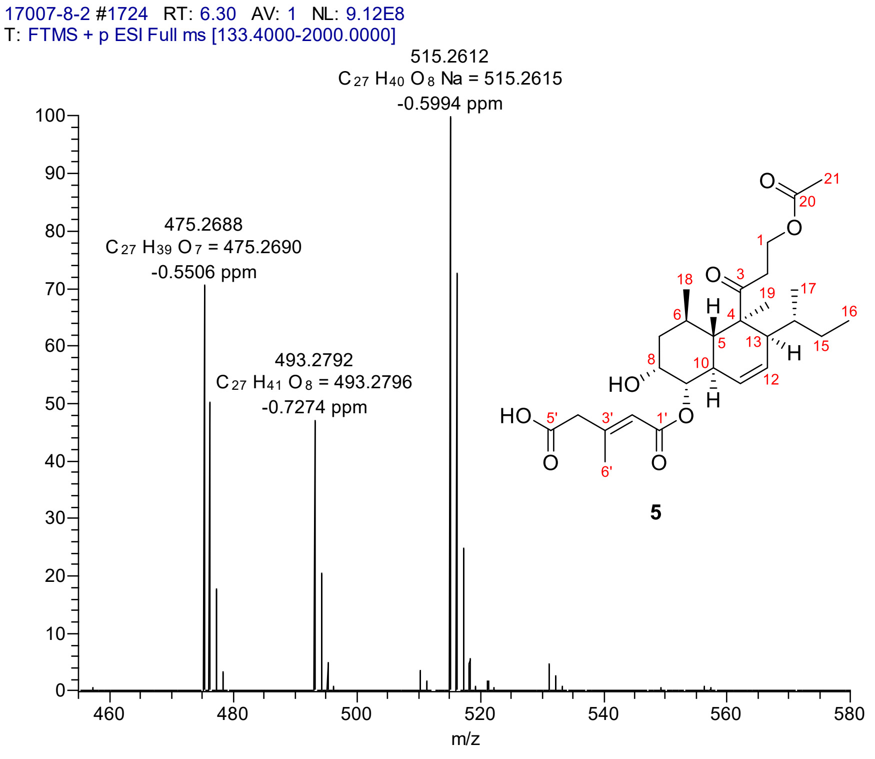

Figure S8a. HR-ESI (Pos)-MS spectrum of tandyukisin O (**5**)

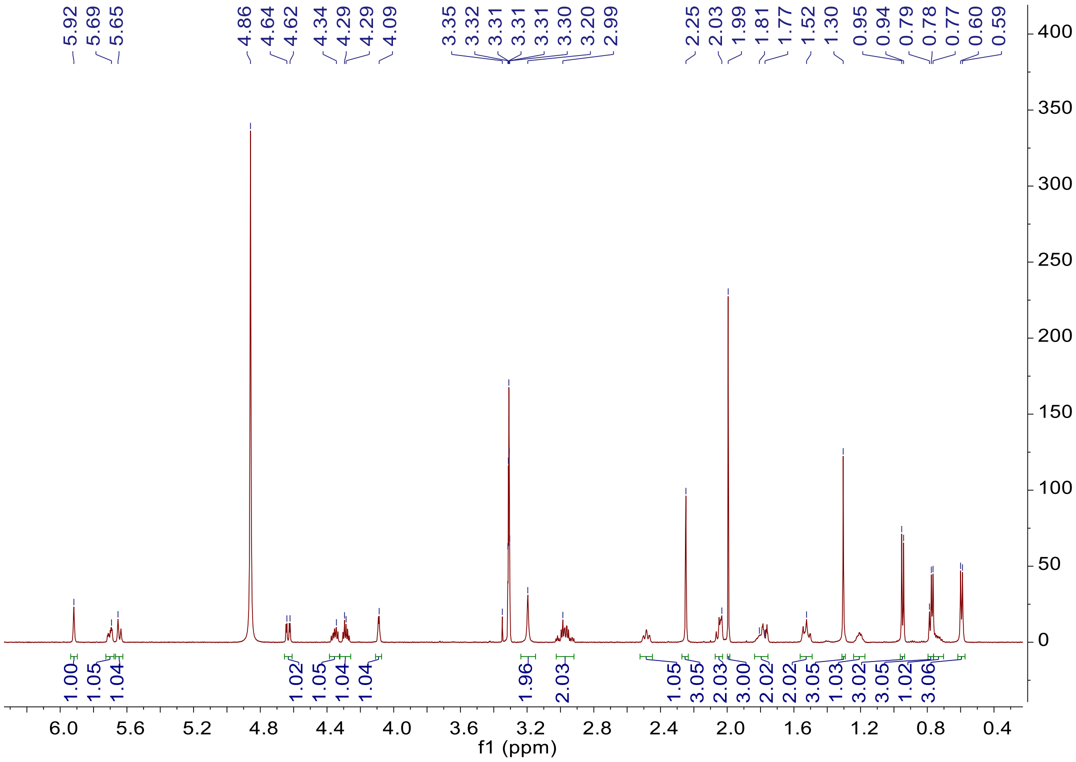

Figure S8b. ^{1}H NMR (600 MHz, Methanol-d_4) spectrum of tandyukisin O (**5**)

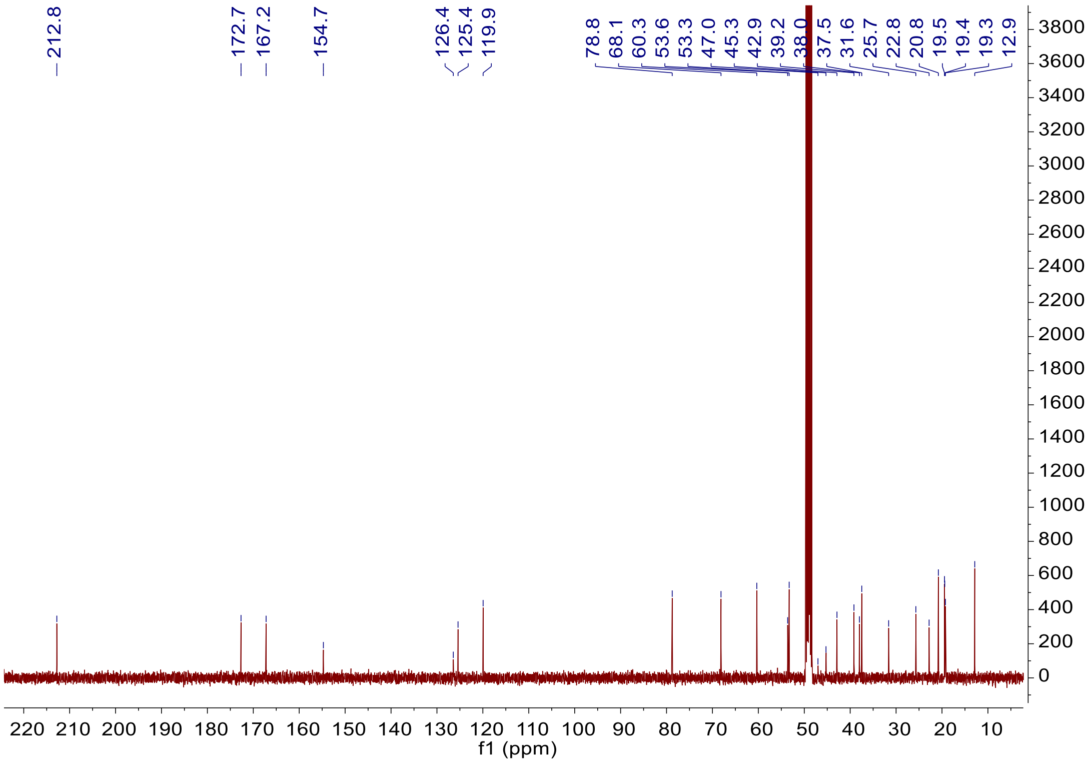

Figure S8c. ^{13}C NMR (150 MHz, Methanol-d_4) spectrum of tandyukisin O (**5**)

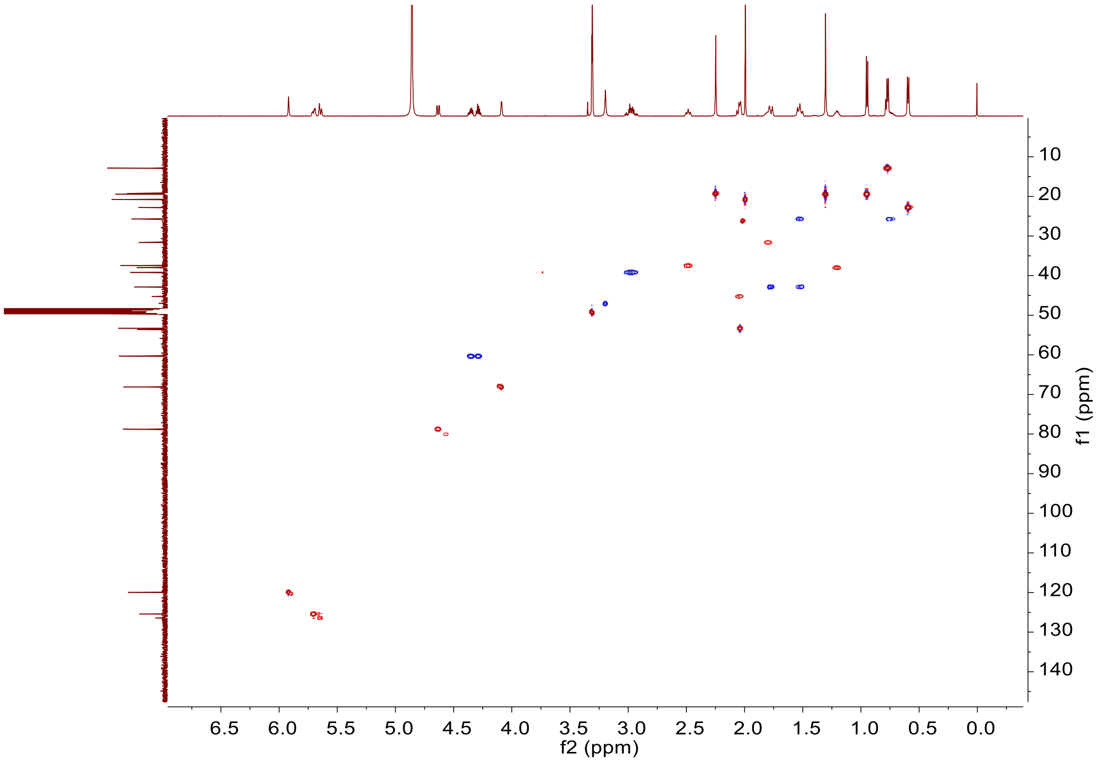

Figure S8d. HSQC spectrum of tandyukisin O (**5**)

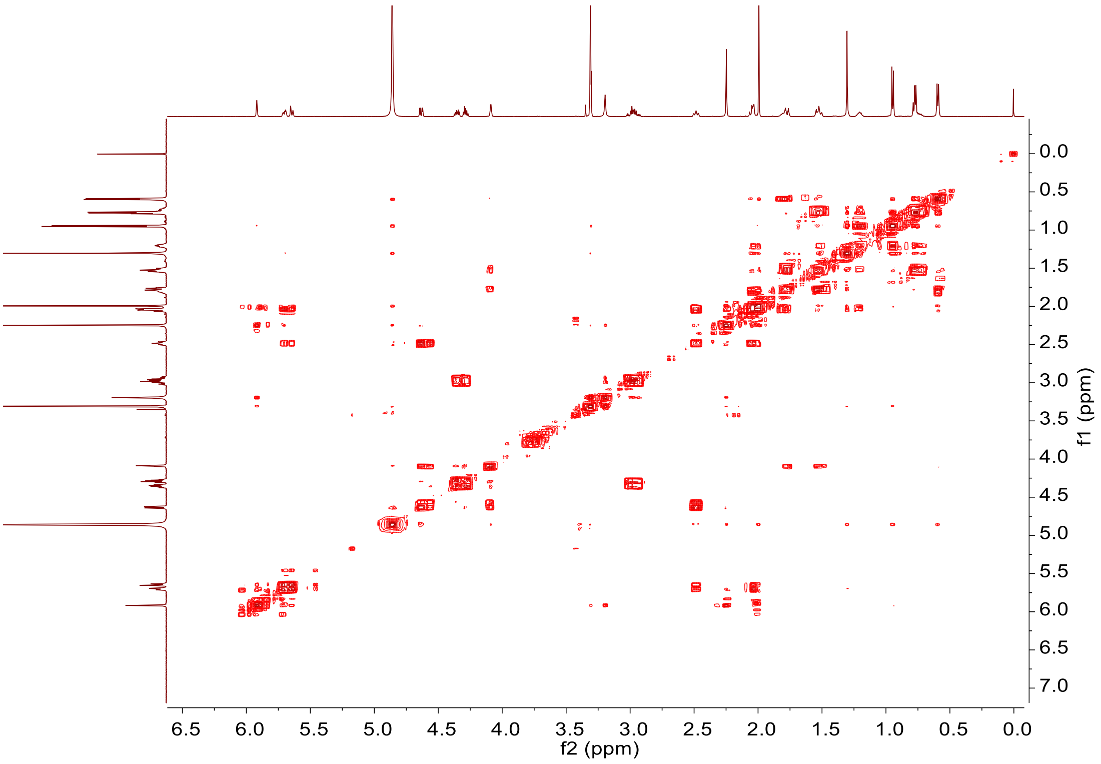

Figure S8e. ^{1}H-^{1}H COSY spectrum of tandyukisin O (**5**)

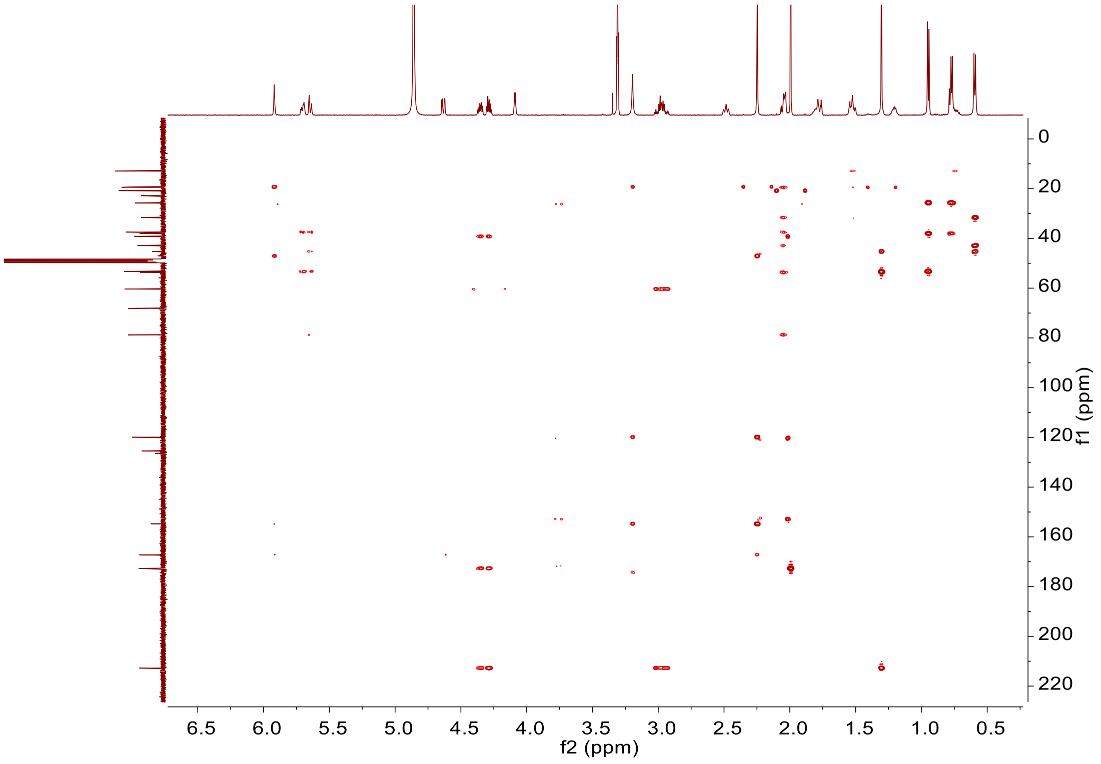

Figure S8f. HMBC spectrum of tandyukisin O (**5**)

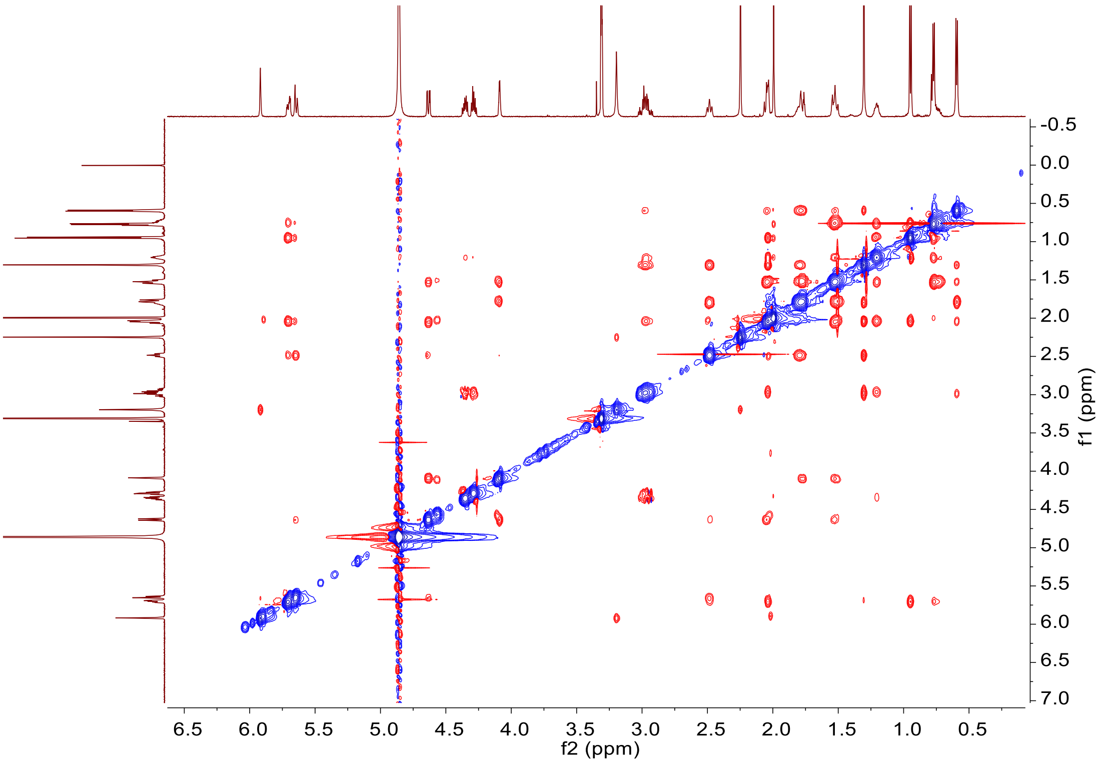

Figure S8g. NOESY spectrum of tandyukisin O (**5**)

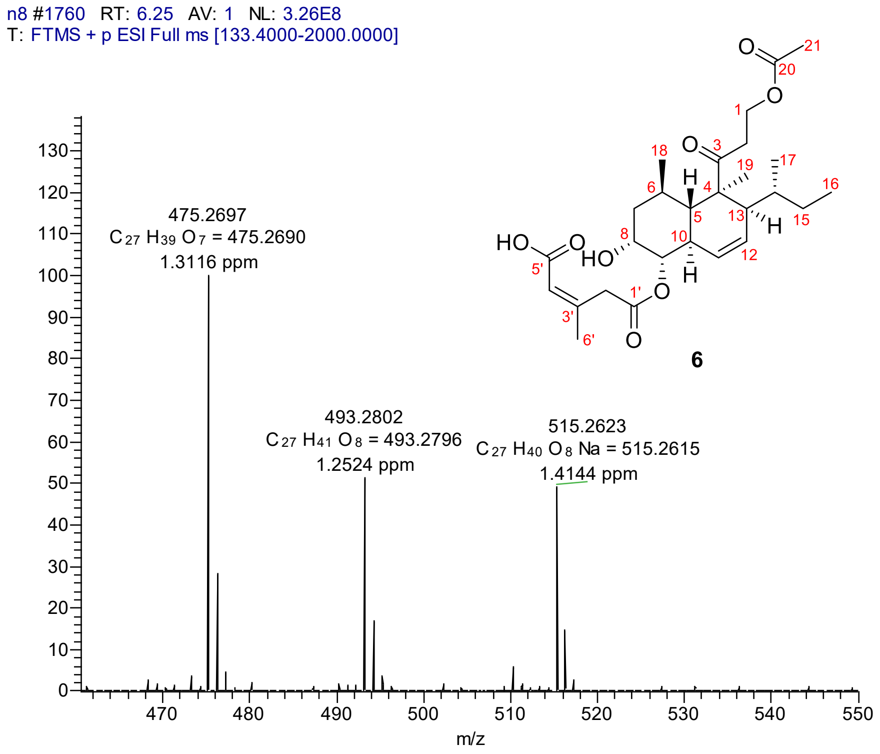

Figure S9a. HR-ESI (Pos)-MS spectrum of tandyukisin P (**6**)

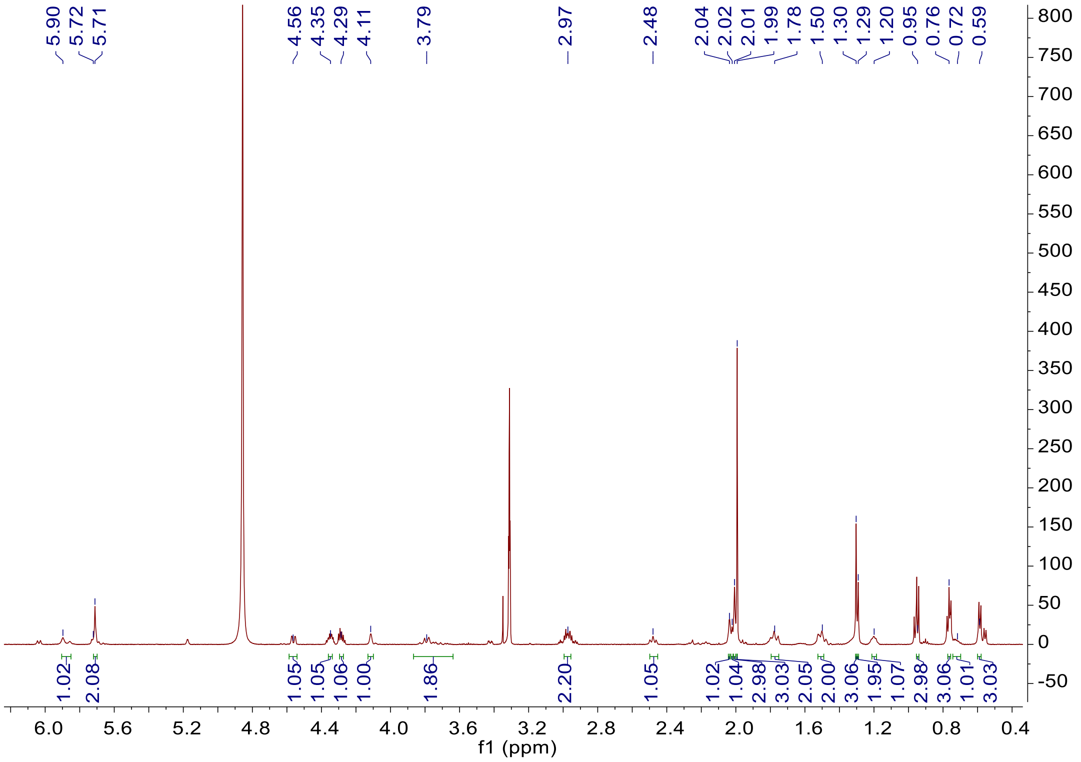

Figure S9b. ^{1}H NMR (600 MHz, Methanol-d_4) spectrum of tandyukisin P (**6**)

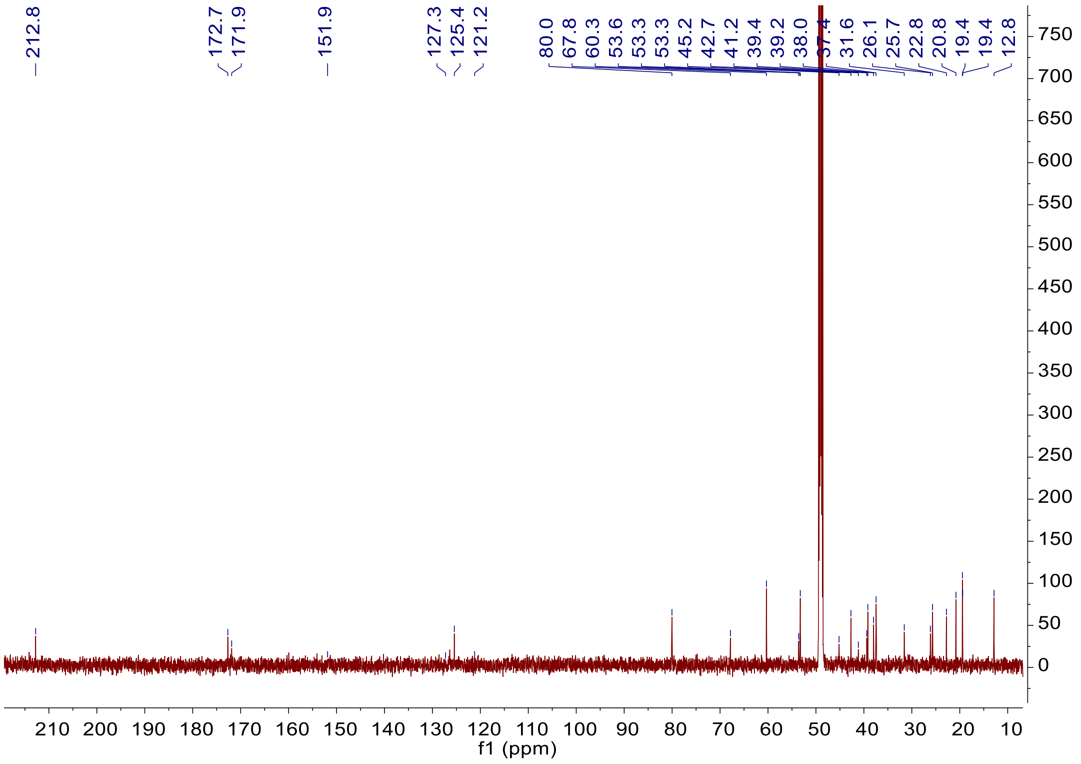

Figure S9c. ^{13}C NMR (150 MHz, Methanol-d_4) spectrum of tandyukisin P (**6**)

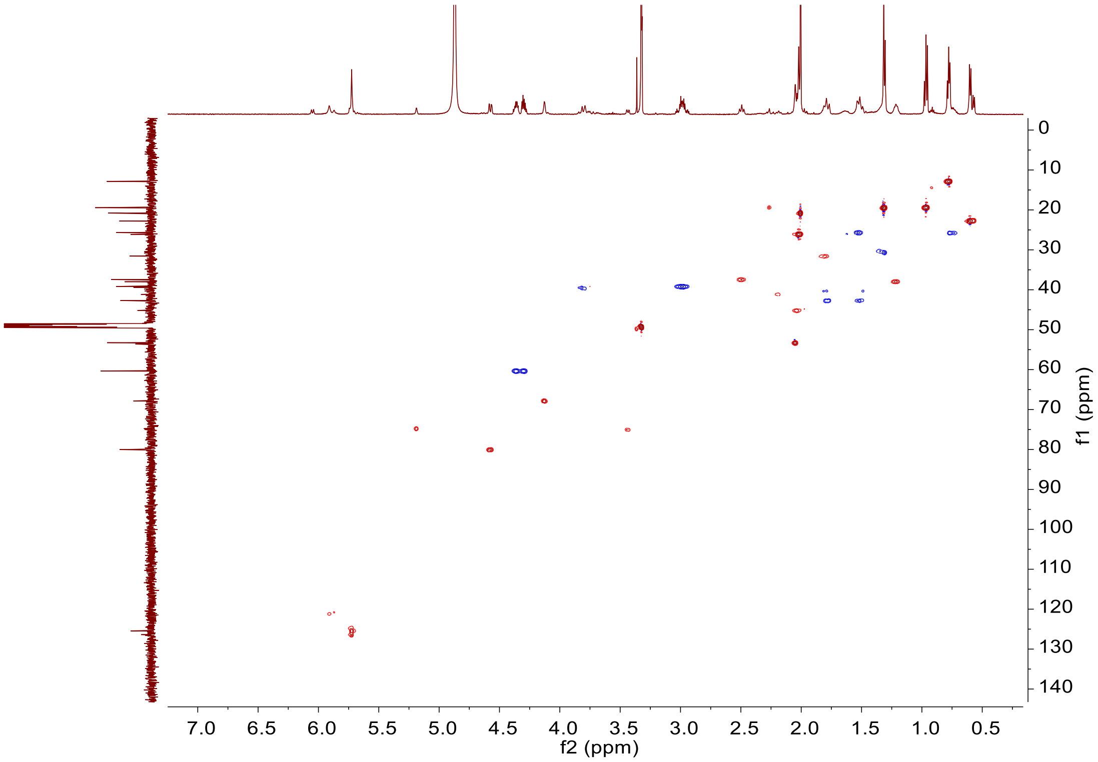

Figure S9d. HSQC spectrum of tandyukisin P (**6**)

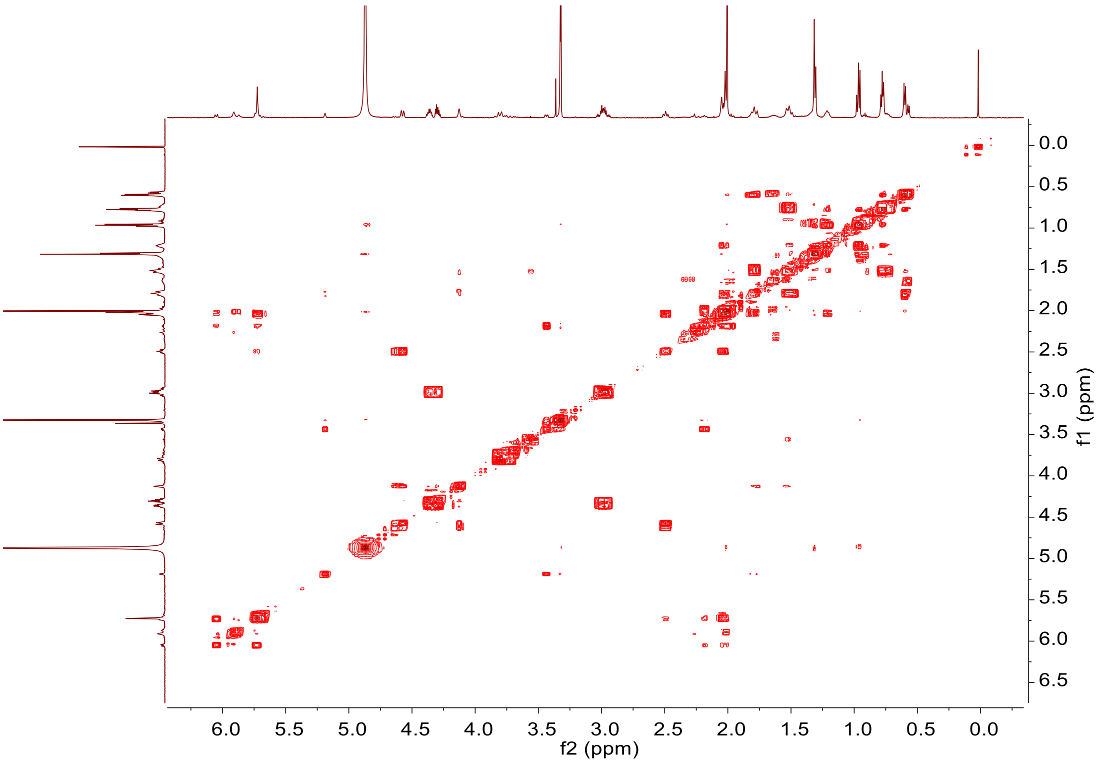

Figure S9e. ^{1}H-^{1}H COSY spectrum of tandyukisin P (**6**)

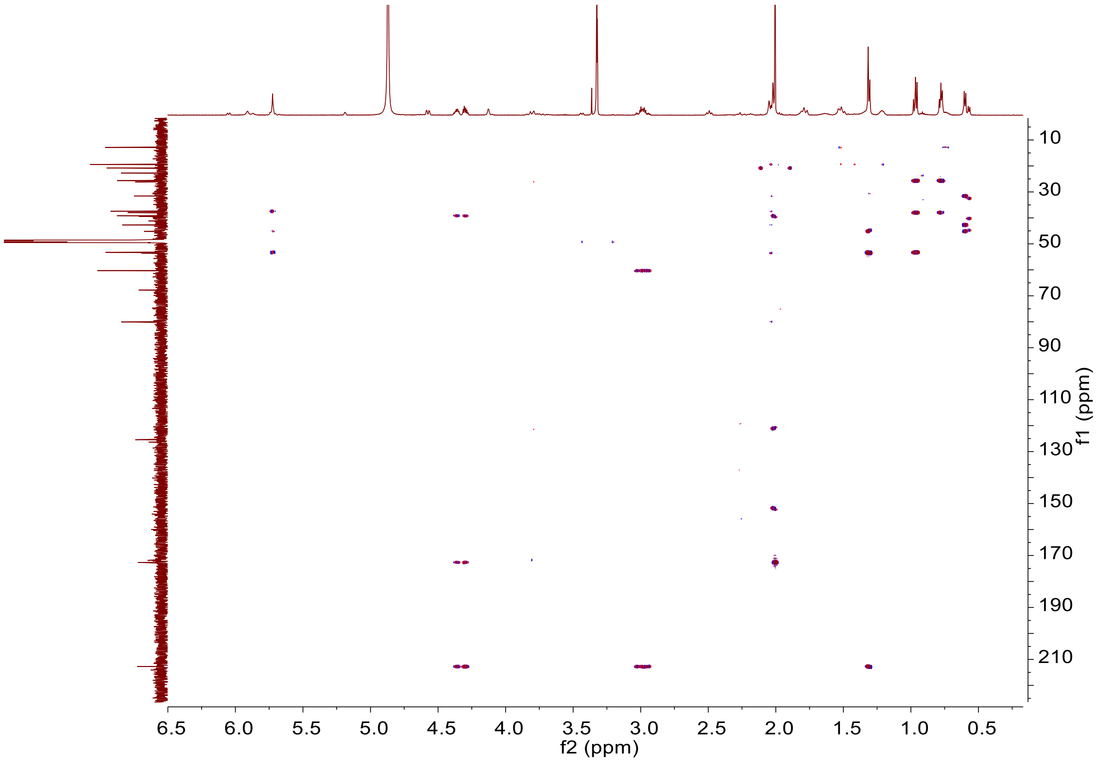

Figure S9f. HMBC spectrum of tandyukisin P (**6**)

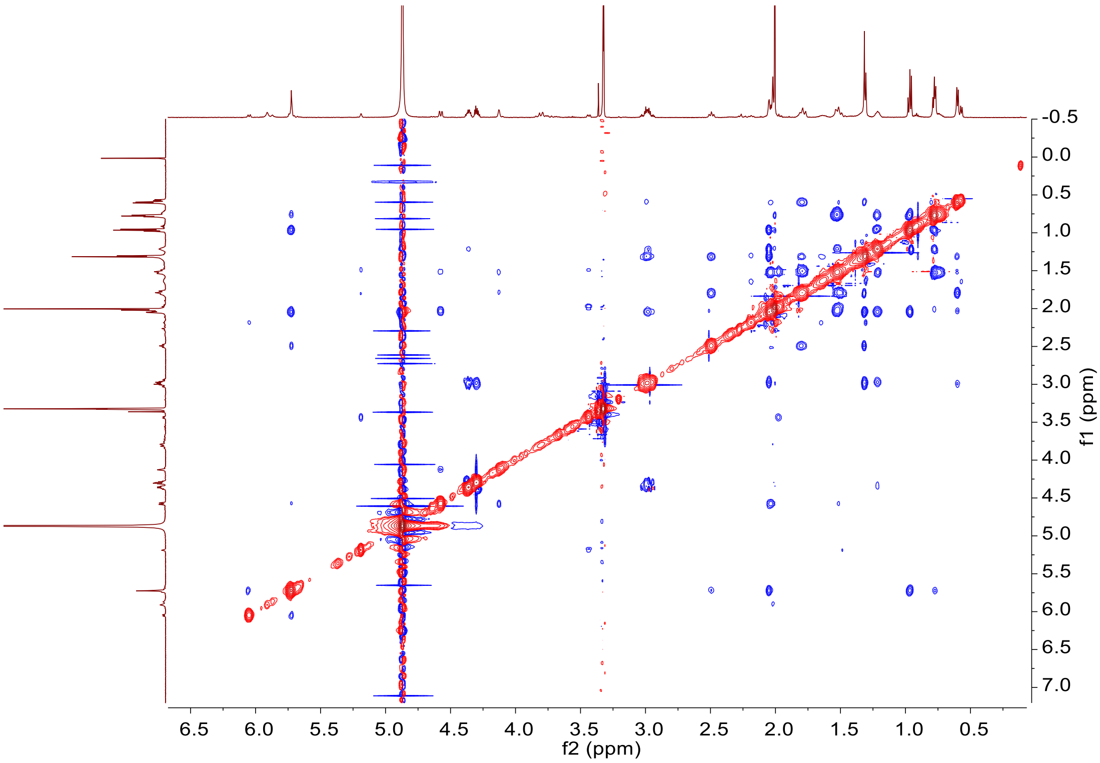

Figure S9g. NOESY spectrum of tandyukisin P (**6**)

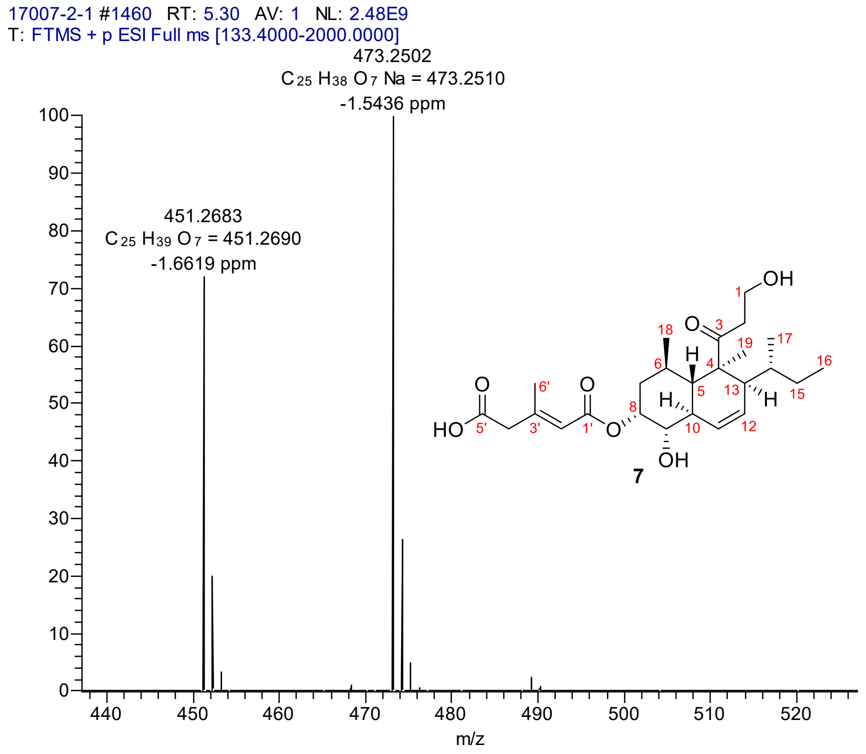

Figure S10a. HR-ESI (Pos)-MS spectrum of trichoharzin (**7**)

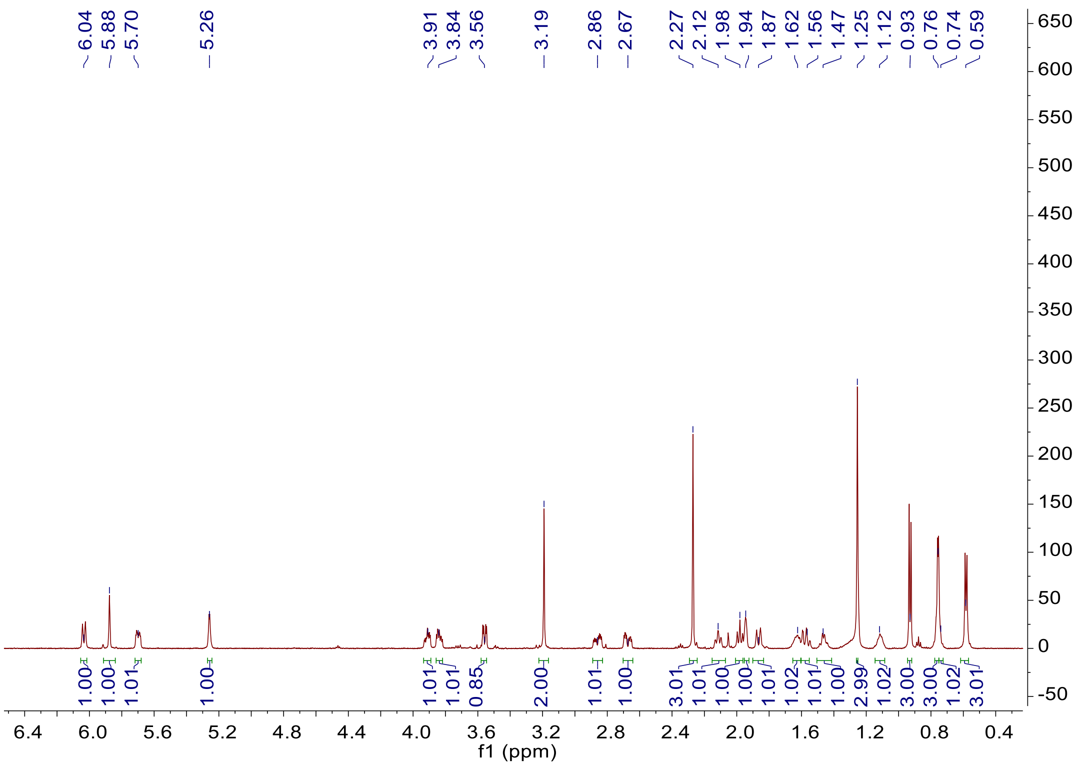

Figure S10b. ^{1}H NMR (600 MHz, $CDCl_3$) spectrum of trichoharzin (**7**)

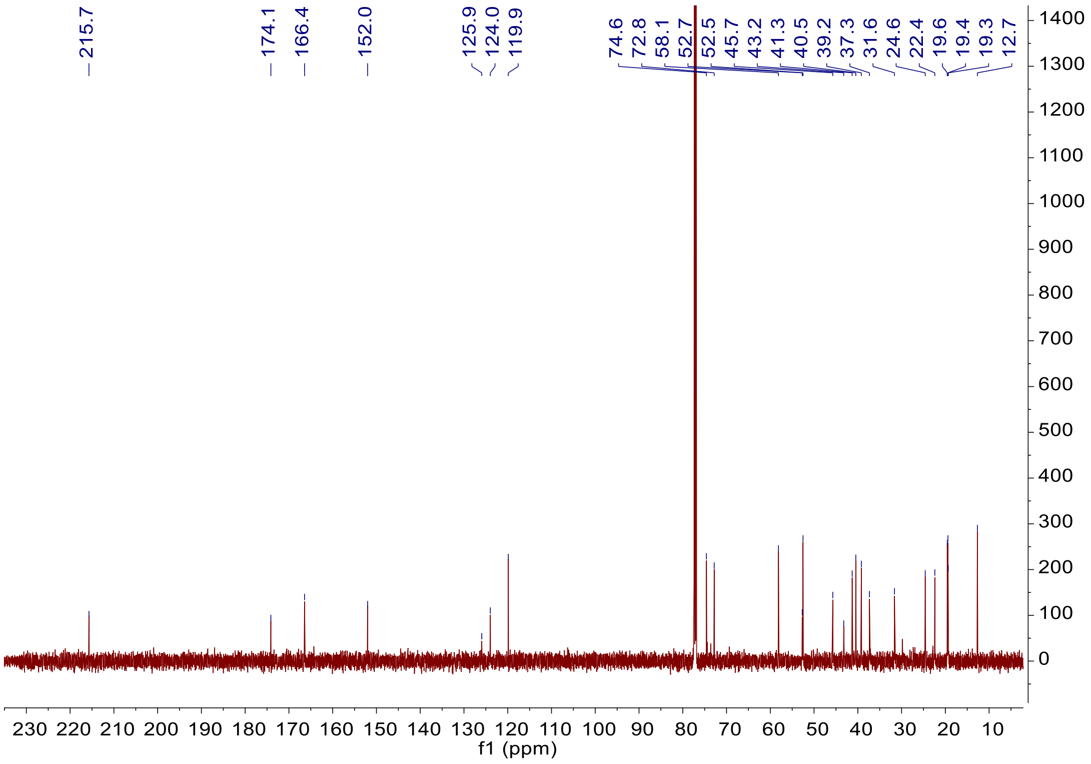

Figure S10c. ^{13}C NMR (150 MHz, $CDCl_3$) spectrum of trichoharzin (**7**)

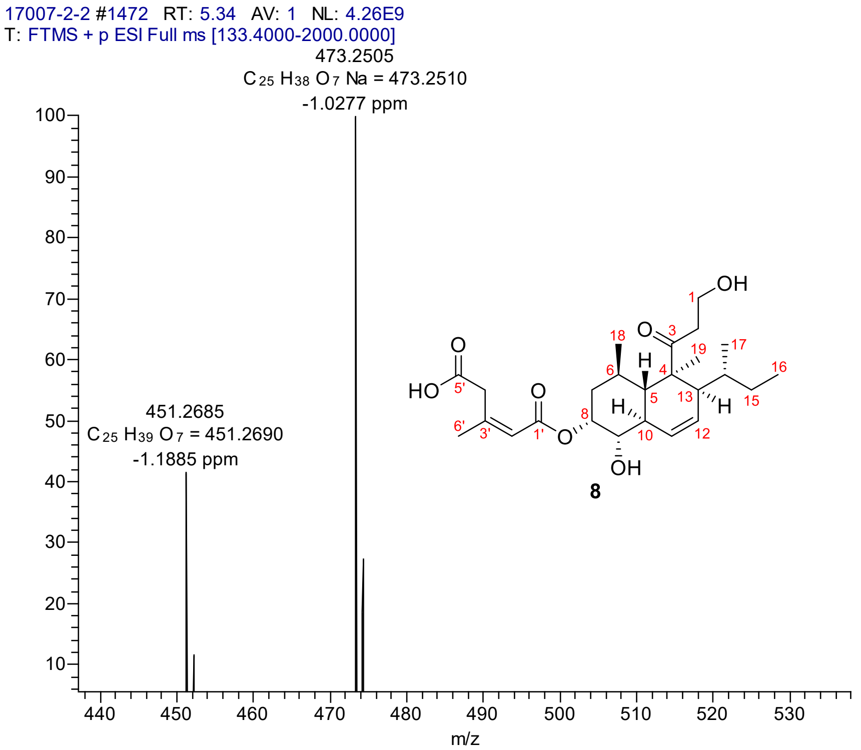

Figure S11a. HR-ESI (Pos)-MS spectrum of tandyukisin J (**8**)

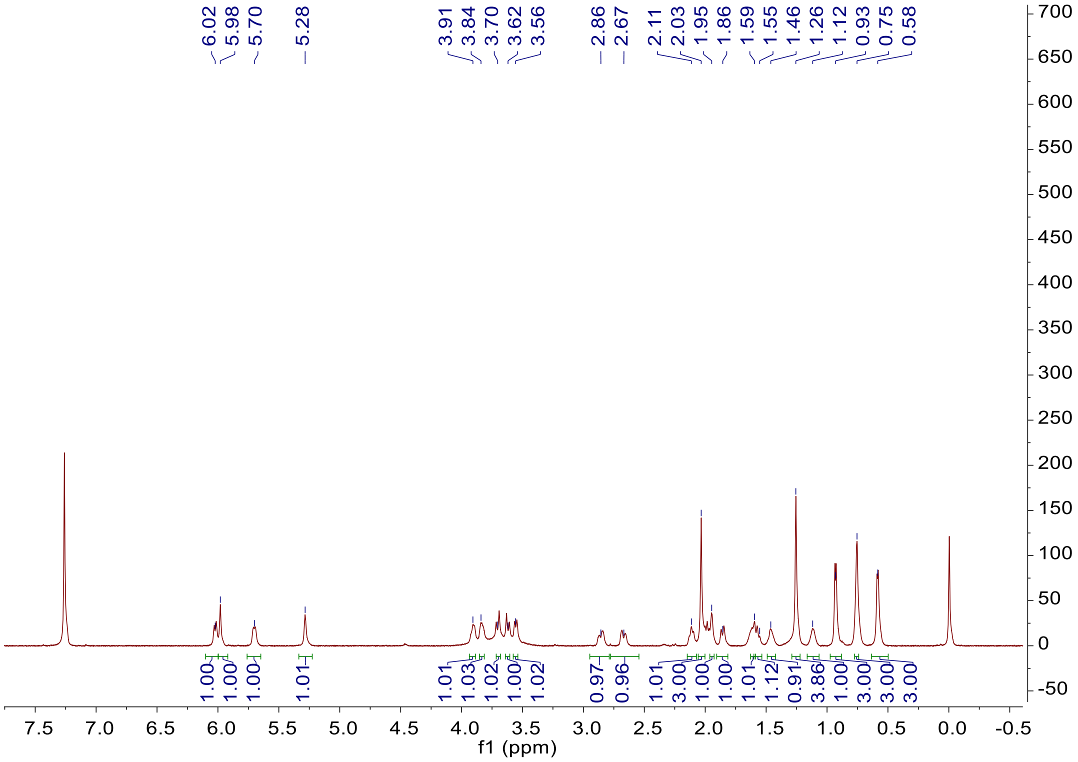

Figure S11b. ^{1}H NMR (600 MHz, $CDCl_3$) spectrum of tandyukisin J (**8**)

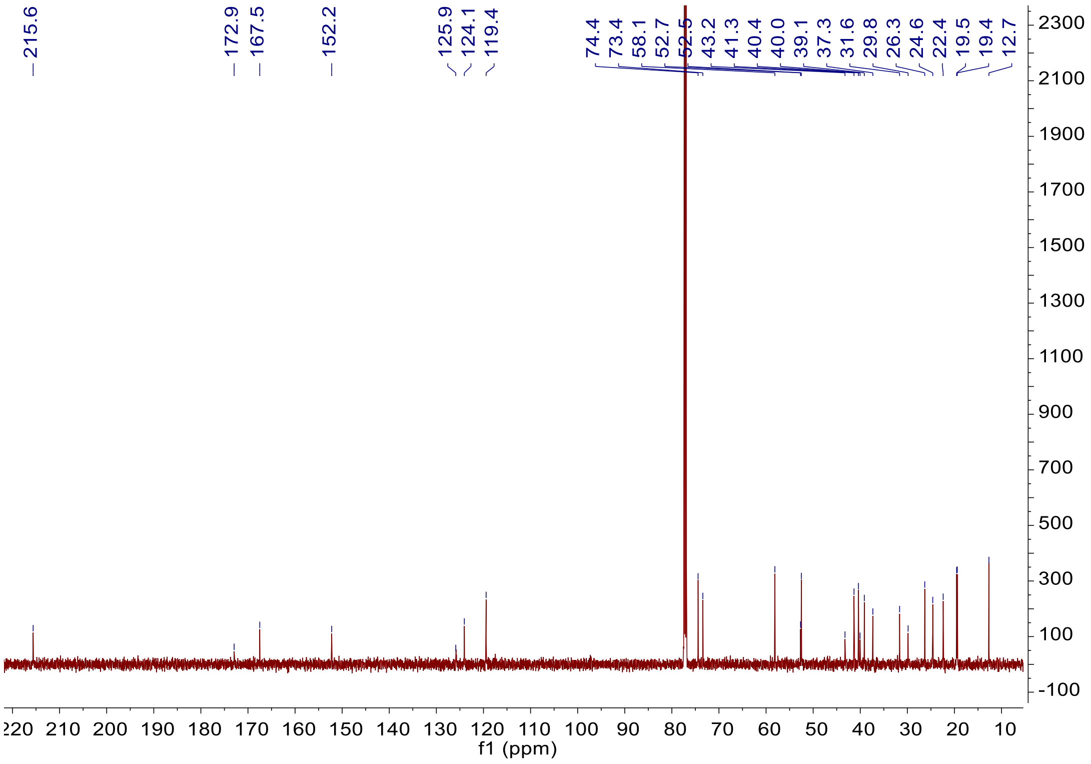

Figure S11c. ^{13}C NMR (150 MHz, $CDCl_3$) spectrum of tandyukisin J (**8**)

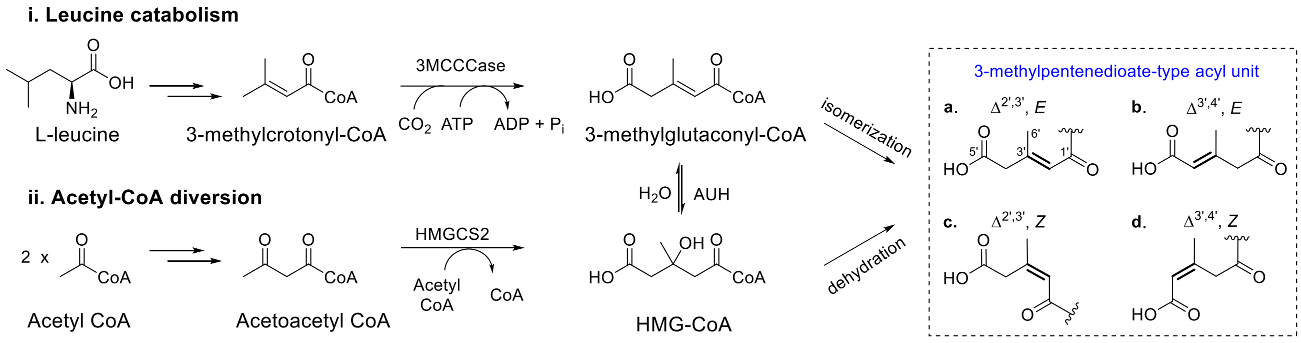

Figure S12. Putative origins of the 3-methylpentenedioate-type acyl unit. (i) Leucine catabolism-derived pathway. (ii) Acetyl-CoA-derived pathway.

Supplementary Tables

Table S1. Annotations of the 23 nodes in the decalin cluster I from the *T. longicollum* 17007 featured-based molecular networking

Node (Observed precursor *m/z*)	t_R	Molecular formula $[M + H]^+$	Calc. *m/z*	$^a\Delta$ (ppm)	Δ*m/z* from seed Trichoharzin (**7**)	Annotation
433.2592	11.38	$C_{25}H_{37}O_6$	433.259	1.696	-18.0109	-H_2O from **7**
433.2593	12.29	$C_{25}H_{37}O_6$	433.259	1.927	-18.0108	-H_2O from **7**
449.2897	16.78	-	-	-	-	-
451.2682	12.29	$C_{25}H_{39}O_7$	451.2696	1.839	-0.0019	isomer of **7**
451.2701	5.33	$C_{25}H_{39}O_7$	451.2696	2.371	0.0000	trichoharzin (**7**)
451.2701	11.90	$C_{25}H_{39}O_7$	451.2696	2.371	0.0000	isomer of **7**
451.2702	6.32	$C_{25}H_{39}O_7$	451.2696	2.593	0.0001	isomer of **7**
451.2702	11.35	$C_{25}H_{39}O_7$	451.2696	2.593	0.0001	isomer of **7**
468.2968	6.08	$C_{25}H_{42}O_7N$	468.2961	2.607	17.0267	+H_3N from **7**
468.2968	5.31	$C_{25}H_{42}O_7N$	468.2961	2.607	17.0267	+H_3N from **7**
475.2697	16.58	$C_{27}H_{39}O_7$	475.2696	1.410	23.9996	+C_2 from **7**
475.2700	11.38	$C_{27}H_{39}O_7$	475.2696	2.041	23.9999	+C_2 from **7**
475.2700	13.04	$C_{27}H_{39}O_7$	475.2696	2.041	23.9999	+C_2 from **7**
475.2701	11.90	$C_{27}H_{39}O_7$	475.2696	2.251	24.0000	+C_2 from **7**
475.2701	12.39	$C_{27}H_{39}O_7$	475.2696	2.251	24.0000	+C_2 from **7**
493.2807	11.90	$C_{27}H_{41}O_8$	493.2801	2.241	42.0106	+C_2H_2O from **7**
493.2807	11.36	$C_{27}H_{41}O_8$	493.2801	2.241	42.0106	+C_2H_2O from **7**
493.2807	13.07	$C_{27}H_{41}O_8$	493.2801	2.241	42.0106	+C_2H_2O from **7**
493.2808	12.39	$C_{27}H_{41}O_8$	493.2801	2.444	42.0107	+C_2H_2O from **7**
510.3064	11.36	$C_{27}H_{41}O_8N$	510.3067	0.502	59.0363	+C_2H_5ON from **7**
510.3064	11.92	$C_{27}H_{41}O_8N$	510.3067	0.502	59.0363	+C_2H_5ON from **7**
510.3065	12.46	$C_{27}H_{41}O_8N$	510.3067	0.698	59.0364	+C_2H_5ON from **7**
610.3218	12.39	-	-	-	-	-

$^a\Delta$ = (Observed *m/z* – Calc. *m/z*)/Calc. *m/z* × 10^6; "-" means less possible to be decalin anologs.

Table S2. Comparative ^{13}C NMR features and side-chain assignments of reported tandyukisin-type decalin polyketides

Compound	δ_{C8}	δ_{C9}	$\Delta\delta_{C8–C9}$	Acylation site	$\delta_{C1'}$	$\delta_{C5'}$	$\lvert\Delta\delta_{C1'–C5'}\rvert$	$\delta_{C6'}$	Side-chain C=C	NMR solvent
**Tandyukisin A**	72.1	74.0	1.9	C8-*O*	166.2	170.2	4.0	19.0	$\Delta^{2',3'}$ (*E*)	$CDCl_3$
**Trichoharzin**	72.7	74.4	1.7	C8-*O*	166.2	174.0	7.8	19.2	$\Delta^{2',3'}$ (*E*)	$CDCl_3$
**Tandyukisin B**	67.6	77.4	9.8	C9-*O*	164.9	170.2	5.3	19.2	$\Delta^{2',3'}$ (*E*)	$CDCl_3$
**Tandyukisin C**	66.7	78.8	12.1	C9-*O*	170.0	168.9	1.1	27.4	$\Delta^{3',4'}$ (*Z*)	$CDCl_3$
**Tandyukisin D**	73.3	74.2	0.9	C8-*O*	168.9	169.8	0.9	26.9	$\Delta^{3',4'}$ (*Z*)	$CDCl_3$
**Tandyukisin E**	67.7	77.0	9.3	C9-*O*	165.0	169.6	4.6	19.2	$\Delta^{2',3'}$ (*E*)	$CDCl_3$
**Tandyukisin F**	72.6	74.4	1.8	C8-*O*	166.3	169.7	3.4	19.2	$\Delta^{2',3'}$ (*E*)	$CDCl_3$
**Tandyukisin G**	74.0	74.3	0.3	C8-*O*	170.2	170.4	0.2	19.4	$\Delta^{3',4'}$ (*E*)	$CDCl_3$
**Tandyukisin H**	67.6	79.1	11.5	C9-*O*	169.0	170.3	1.3	19.5	$\Delta^{3',4'}$ (*E*)	$CDCl_3$
**Tandyukisin I**	67.7	77.5	9.8	C9-*O*	165.1	174.3	9.2	19.5	$\Delta^{2',3'}$ (*E*)	$CDCl_3$
**Tandyukisin J**[1]	73.6	74.4	0.8	C8-*O*	167.5	172.8	5.3	26.2	$\Delta^{2',3'}$ (*Z*)	$CDCl_3$
**Tandyukisin J**[2]	67.5	78.2	10.7	C9-*O*	165.8	173.8	8.0	26.2	$\Delta^{2',3'}$ (*Z*)	$CDCl_3$

$\Delta\delta_{C8–C9}$ represents the difference between the ^{13}C NMR chemical shifts of C8 and C9; $\lvert\Delta\delta_{C1'–C5'}\rvert$ represents the absolute difference between the ^{13}C NMR chemical shifts of C1′ and C5′. [1]reported on *Mar. Drugs* 2023, 21(11), 566; [2]reported on *Nat. Prod. Res* 2026, 40, 4256–4263. All chemical shifts are expressed in ppm.

Table S3. ^{1}H and ^{13}C NMR Data of trichoharzin (**7**) and tandyukisin J (**8**) in $CDCl_3$

Pos.	**7**[a]		**8**[a]	
	δ_C, type	δ_H, mult (*J* in Hz)	δ_C, type	δ_H, mult (*J* in Hz)
1α	58.1, CH_2	3.84, m	58.1, CH_2	3.84, m
1β		3.91, m		3.91, m
2α	41.3, CH_2	2.67, ddd (18.9, 9.6, 2.6)	41.3, CH_2	2.67, brd
2β		2.86, ddd (18.9, 10.9, 3.5)		2.86, brd
3	215.7, C		215.6, C	
4	52.7, C		52.7, C	
5	43.2, CH	1.98, t (9.9)	43.2, CH	1.95, brs
6	31.6, CH	1.62, m	31.6, CH	1.59, m
7α	39.2, CH_2	1.87, dt (5.6)	39.1, CH_2	1.86, d (5.7)
7β		1.56, td (2.4)		1.55, overlapped
8	72.8, CH	5.26, q	73.4, CH	5.28, s
9	74.6, CH	3.56, dd (11.0, 3.4)	74.4, CH	3.56, d (11.0, 3.4)
10	40.5, CH	2.12, td (2.4)	40.4, CH	2.11, t
11	124.0, CH	6.04, d (10.5)	125.9, CH	6.02, d (10.6)
12	125.9, CH	5.70, m	124.1, CH	5.70, d (10.1)
13	52.5, CH	1.94, brd	52.5, CH	1.95, brs
14	37.3, CH	1.12, m	37.3, CH	1.12, brs
15α	24.6, CH_2	0.74, m	24.6, CH_2	0.75, overlapped
15β		1.47, m		1.46, brs
16	12.7, CH_3	0.76, d (3.5)	12.7, CH_3	0.75, s
17	19.3, CH_3	0.93, d (6.7)	19.3, CH_3	0.93, d (6.7)
18	22.4, CH_3	0.59, d (6.7)	22.4, CH_3	0.58, d (6.7)
19	19.6, CH_3	1.25, s	19.5, CH_3	1.26, s
1′	166.4, C		167.5, C	
2′	119.9, CH	5.88, s	119.4, CH	5.98, s
3′	152.0, C		152.2, C	
4′α	45.7, CH_2	3.19, s	40.0, CH_2	3.56, brd
4′β				3.70, brd
5′	174.1, C		172.9, C	
6′	19.4, CH_3	2.27, s	26.3, CH_3	2.03, s

Table S4. *In vitro* bioactivity evaluation of compounds **1**–**8**

**Organism**	**Minimum inhibitory concentration (μg/mL) for pathogens and IC_{50} (μM) for cell lines**								
	**1**	**2**	**3**	**4**	**5**	**6**	**7**	**8**	**Positive control**
M. tuberculosis **H37Ra**	12.5	> 50	> 50	> 50	> 50	12.5	> 50	> 50	0.03[a]
**A549**	> 50	> 50	> 50	> 50	> 50	> 50	> 50	> 50	0.879 ± 0.094[b]
**MKN-45**	> 50	> 50	> 50	> 50	> 50	> 50	> 50	> 50	0.048 ± 0.002[b]
**HCT 116**	> 50	> 50	> 50	> 50	> 50	> 50	> 50	> 50	1.102 ± 0.100[b]
**HeLa**	> 50	> 50	> 50	> 50	> 50	> 50	> 50	> 50	0.103 ± 0.004[b]
**K-562**	> 50	> 50	> 50	> 50	> 50	> 50	> 50	> 50	0.742 ± 0.009[b]
**786-O**	> 50	> 50	> 50	> 50	> 50	> 50	> 50	> 50	0.138 ± 0.012[b]
**TE-1**	> 50	> 50	> 50	> 50	> 50	> 50	> 50	> 50	0.254 ± 0.018[b]
**5637**	> 50	> 50	> 50	> 50	> 50	> 50	> 50	> 50	0.065 ± 0.006[b]
**GBC-SD**	> 50	> 50	> 50	> 50	> 50	> 50	> 50	> 50	0.872 ± 0.017[b]
**L-02**	> 50	> 50	> 50	> 50	> 50	> 50	> 50	> 50	0.226 ± 0.008[b]
**HL-60**	> 50	> 50	> 50	> 50	> 50	> 50	> 50	> 50	0.103 ± 0.004[b]

[a]Isoniazid, [b]Doxorubicin hydrochloride

Table S5. Deduced functions of ORFs in tandyukisins (*tan*) BGC from *T. longicollum* 17007 based on top BLASTP matches

ORF	Size (aa)	Proposed function	Origin	Accession no.	Identity (%)	Similarity (%)
tanA	574	Hexose transporter 2	*Kluyveromyces lactis*	P53387.1	39	54
tanB	834	Trehalose-phosphatase	*Saccharomyces cerevisiae* S288C	P31688.3	40	55
tanC	146	Elongation factor Ts	*Prosthecochloris aestuarii* DSM 271	B4S429.1	11	17
tanD	714	Quinic acid utilization activator	*Aspergillus nidulans* FGSC A4	P10563.2	16	24
tanE	361	Medium chain reductase	*Acremonium* sp.	A0A8F4NVX8.1	33	46
tanF	300	Oxidoreductase	*Penicillium oxalicum*	A0A1W5T323.1	27	51
tanG	335	NADPH-dependent FMN reductase	*Sinorhizobium meliloti* 1021	Q92R45.1	40	50
tanH	295	Arsenite methyltransferase	*Methanosarcina acetivorans* C2A	Q8TJK1.1	35	48
tanI	243	Pleckstrin homology domain-containing family H member 1	*Mus musculus*	Q80TI1.2	4	6
tanJ	416	Probable NADH dehydrogenase	*Dictyostelium discoideum*	Q55CD9.2	31	50
tanK	2917	Reducing polyketide synthase	*Penicillium decumbens*	A0A1V6PAF7.1	45	61
tanL	490	Cytochrome P450 monooxygenase	*Neocamarosporium betae*	Q6F5E4.1	39	58
tanM	384	Trans-enoyl reductase	*Penicillium decumbens*	A0A1V6PAP3.1	48	64
tanN	331	Short-chain dehydrogenase/reductase	*Neocamarosporium betae*	A0A0C6DRT7.1	42	57
tanO	592	FAD-linked oxidoreductase	*Penicillium decumbens*	A0A1V6PBT1.1	45	61
tanP	748	Enoyl-CoA isomerase/CoA-acyltransferase	*Penicillium crustosum*	A0A481WNM8.1	56	68
tanQ	215	LysM domain-containing protein ARB_03438	*Trichophyton benhamiae* CBS 112371	D4B4P8.1	21	28
